# The Transcriptomic and Phenotypic Response of the Melanized Yeast *Exophiala dermatitidis* to Ionizing Particle Exposure

**DOI:** 10.3389/fmicb.2020.609996

**Published:** 2021-01-12

**Authors:** Zachary Schultzhaus, Amy Chen, Igor Shuryak, Zheng Wang

**Affiliations:** ^1^Center for Biomolecular Science and Engineering, United States Naval Research Laboratory, Washington, DC, United States; ^2^Virginia Tech Carilion School of Medicine, Roanoke, VA, United States; ^3^Center for Radiological Research, Columbia University Irving Medical Center, New York, NY, United States

**Keywords:** melanin, ionizing particle, transcriptomic, radiobiology, microbiology, fungi, yeast

## Abstract

Fungi can tolerate extremely high doses of ionizing radiation compared with most other eukaryotes, a phenomenon encompassing both the recovery from acute exposure and the growth of melanized fungi in chronically contaminated environments such as nuclear disaster sites. This observation has led to the use of fungi in radiobiology studies, with the goal of finding novel resistance mechanisms. However, it is still not entirely clear what underlies this phenomenon, as genetic studies have not pinpointed unique responses to ionizing radiation in the most resistant fungi. Additionally, little work has been done examining how fungi (other than budding yeast) respond to irradiation by ionizing particles (e.g., protons, α-particles), although particle irradiation may cause distinct cellular damage, and is more relevant for human risks. To address this paucity of data, in this study we have characterized the phenotypic and transcriptomic response of the highly radioresistant yeast *Exophiala dermatitidis* to irradiation by three separate ionizing radiation sources: protons, deuterons, and α-particles. The experiment was performed with both melanized and non-melanized strains of *E. dermatitidis*, to determine the effect of this pigment on the response. No significant difference in survival was observed between these strains under any condition, suggesting that melanin does not impart protection to acute irradiation to these particles. The transcriptomic response during recovery to particle exposure was similar to that observed after γ-irradiation, with DNA repair and replication genes upregulated, and genes involved in translation and ribosomal biogenesis being heavily repressed, indicating an attenuation of cell growth. However, a comparison of global gene expression showed clear clustering of particle and γ-radiation groups. The response elicited by particle irradiation was, in total, more complex. Compared to the γ-associated response, particle irradiation resulted in greater changes in gene expression, a more diverse set of differentially expressed genes, and a significant induction of gene categories such as autophagy and protein catabolism. Additionally, analysis of individual particle responses resulted in identification of the first unique expression signatures and individual genes for each particle type that could be used as radionuclide discrimination markers.

## Introduction

The impact of ionizing radiation (IR) on biological systems has been a focus of research for decades, and it remains a relevant and fruitful area of research for several reasons. First, the effects of IR are similar in nearly every type of cell: reactive oxygen species (ROS) produced through the radiolysis of water result in protein oxidation and DNA damage, particularly double strand breaks (DSBs), which are fatal to cells if misrepaired (Ward, 1988; Riley, 1994; Battista et al., 1999; United Nations Scientific Committee on the Effects of Atomic Radiation, 2000). Distantly related organisms, therefore, essentially have to solve the same problem when recovering from IR exposure, imparting value on comparative studies. This damage is also similar to that encountered in cancer and age-related diseases, giving radiobiological studies broad application. Second, IR exposure is an ever-present threat, with risks in the clinic (e.g., for diagnostics and cancer treatment), in combat zones (e.g., nuclear warfare), sites of nuclear disasters (e.g., Chernobyl, Fukushima), and for extended missions in deep space. Finally, despite the massive radiobiological literature that has accumulated over the years, treatments for IR exposure are elusive, with virtually no prophylactic compounds available and little recourse for acute exposure (DiCarlo et al., 2017).

Research continues, therefore, to detail the shared and unique IR resistance strategies throughout life. Two broad veins of research have emerged from this work. The first focuses on the importance of DNA repair enzymes. In bacteria, this led to the discovery of the *rec* genes which facilitate homologous recombination (HR; McEntee et al., 1979; Roberts et al., 1979; Sargentini and Smith, 1986; Clark, 1991; Gruenig et al., 2011). A RecA homolog is known in eukaryotes [Rad51 (Shinohara et al., 1992)], though further studies in budding yeast and other models have described a much more complex DNA repair repertoire, including non-homologous end-joining (NHEJ) pathways that are much less common in prokaryotes (Critchlow and Jackson, 1998). In a parallel vein of research, intracellular Mn^2+^ complexes and other small metabolites have been shown to protect proteins from IR-induced oxidation and correlate with IR resistance in many organisms (Daly et al., 2004; Sharma et al., 2017; Doble and Miklos, 2018).

More recently, the observation that dozens of mold species could be isolated in and around the damaged nuclear reactor at Chernobyl (Zhdanova et al., 2000) has sparked interest in fungi outside of *Saccharomyces cerevisiae* for radiobiological studies. These organisms are indeed good models for this work – they have small, easily manipulated genomes and can be grown and irradiated easily (Loftus et al., 2005; Dadachova et al., 2007; Zakrzewska et al., 2011; Chen et al., 2014; Jung et al., 2016). Significant findings from studies in these organisms include the stimulation of growth of some fungi by low dose IR (Dadachova et al., 2007, 2008; Dadachova and Casadevall, 2008); the possibility that melanin in the cell walls of certain fungi may protect against IR damage (Dadachova et al., 2008; Pacelli et al., 2017a); the high IR resistance of organisms such as *Ustilago maydis* (Holliday, 1975; Lee and Yarranton, 1982; Holloman et al., 2007; Milisavljevic et al., 2018), *Cryptococcus neoformans* (Shuryak et al., 2014; Jung et al., 2016; Pacelli et al., 2017a; Schultzhaus et al., 2019), *Cryomyces antarcticus* (Pacelli et al., 2017b), and *Exophiala dermatitidis* (Romsdahl et al., 2020; Schultzhaus Z. et al., 2020; Schultzhaus Z. S. et al., 2020); and the discovery of novel IR resistance-associated proteins through transcriptomics and targeted mutagenesis (Jung et al., 2016; Schultzhaus et al., 2019; Schultzhaus Z. et al., 2020).

The vast majority of studies looking at the response to IR in fungi, however, have been done using either X-rays or γ-rays. These types of IR are not entirely indicative of most situations that are encountered in the clinic or in the field. Radiation treatments, nuclear accidents, and environments with high background IR (e.g., outer space), almost never contain a spectrum of pure γ- or X-rays. Rather, they involve ionizing particles of higher Relative Biological Effectiveness (RBE) and Linear Energy Transfer (LET) (Cucinotta and Durante, 2006). RBE refers to the ability of a type of IR to damage cells relative to a similar dose of γ-rays, while LET refers to energy transfer to a medium an ionizing particle produces per unit of track length. The distinction in the biological effects between high and low LET IR sources may be great, as charged particles are known to cause distinct types of DNA damage (e.g., more complex and clustered lesions), and could interact with other cellular structures (e.g., cell membranes, proteins, organelles, and melanin) to a greater extent than IR from photons (Ward, 1988; Hada and Georgakilas, 2008). For example, carbon ion (high LET) induced specific mutations in the *S. cerevisiae* and the filamentous fungus *Neurospora crassa* when compared with lower LET irradiation sources [X-rays or γ-rays (Ma et al., 2018; Matuo et al., 2019)], and irradiation with high or low LET sources produce different cell killing rates based on strain melanization status in *C. neoformans* (Pacelli et al., 2017a).

We are interested in the factors that mediate the response to IR exposure in fungi, and have previously characterized how melanin affects the response of *E. dermatitidis* to γ-IR, because it is an intrinsically melanized fungus that is highly radioresistant (Robertson et al., 2012; Romsdahl et al., 2020; Schultzhaus Z. et al., 2020). Acute and chronic γ-IR exposures induced dramatic transcriptomic responses in *E. dermatitidis* that allowed us to identify previously uncharacterized proteins important for its resistance, but nothing is currently known about how these results relate to the effects that ionizing particles have on this organism. Therefore, in this study we have characterized the transcriptomic and phenotypic response of melanized and non-melanized *E. dermatitidis* strains to ionizing particles of various LET (protons, deuterons, and α-particles) to understand the effects these particles have on this organism and how these differ from the effects produced by γ-IR.

## Materials and Methods

### Growth of Fungal Cultures

*Exophiala dermatitidis* cultures (both melanized and non-melanized) were grown in yeast peptone dextrose (YPD) liquid or solid (adding 1.5% agar) medium at 30°C throughout the experiment. To prepare an individual biological replicate for irradiation, a single colony of the appropriate strain, stored on YPD medium, was inoculated into 2 mL YPD and grown in a shaking incubator set at 30°C and shaking at 200 RPM, for 2 days, after which the resulting culture was diluted 1:1000 in 40 mL of YPD and grown for an additional 2 days. At this point, the cell concentration of the resulting culture was measured using a Cellometer (Nexcelom Bioscience, MA, United States) set to measure clustered yeast cells. Cells were then centrifuged at 4000 × *g* for 5 m and re-suspended in YPD to a final concentration of 1 × 10^9^ cells/mL for radiation exposure. Three biological replicates for each strain were grown for irradiation in this experiment.

### Irradiation of *E. dermatitidis*

Ionizing particles, unlike other forms of IR such as γ-radiation, have limited penetration into materials, depending on the particle size and the composition of the materials (Fano, 1963). α-particles, for example, could potentially be blocked by only a few layers of *E. dermatitidis* cells. Therefore, to ensure complete and homogeneous cell exposure, cells were irradiated in chambers composed of a 10 μm thick Mylar sheet stretched across the bottom of a metal ring. A 50 μL drop of concentrated cell suspension (1 × 10^9^ cells/mL) was placed on the center of the sheet, and was carefully flattened under an 18 mm × 18 mm glass coverslip. Particle beams entered the chamber from below, passing first through the Mylar sheet and then through the cell layer.

Due to the length of sample processing and technical limitations of switching between particle sources, proton and α-particle irradiations were performed on 1 day, and their control samples were pooled, while growth and processing of cells for the deuteron irradiation was shifted by 1 day, and had its own set of controls. Total doses used were 125 Gy, 250 Gy, and 500 Gy for α-particle irradiations, 420 Gy, 840 Gy, and 1680 Gy for proton irradiations, and 250 Gy, 500 Gy, and 1000 Gy for deuteron irradiations. This corresponded to exposures to 500 Gy, 1000 Gy, and 2000 Gy irradiation by γ-rays, doses which we used previously to compare the IR resistance of melanized and non-melanized *E. dermatitidis* strains (Schultzhaus Z. et al., 2020). After exposure, cells were collected by carefully lifting each coverslip and removing the cells adhered to it, and to the Mylar sheet, through addition of 3 mL of sterile ddH_2_O and vigorous pipetting. To obtain enough cells for RNA extraction and plating, cells from three different dishes were pooled. The resulting cell suspensions, each totaling approximately 9 mL, were added to 50 mL tubes.

To determine the cell survival, 100 μL was collected from each cell suspension and diluted in an additional 900 μL of ddH_2_O, followed by counting on a Cellometer. Each suspension was then diluted further to appx. 5 × 10^3^ cells/mL, and 100 μL of this suspension was plated on each of two solid YPD plates, to obtain a total of six plates per condition and strain. CFUs were then counted after a 5-day incubation at 30°C, and from these measurements, survival after each exposure was determined.

### Modeling of Survival Data

The standard linear quadratic (LQ) model of radiation-induced cell death is commonly used in radiation biology and oncology. This formalism predicts the clonogenic surviving fraction (*S*) of cells exposed to an acute dose *D* by the following equation, where *PE* is the cell plating efficiency without radiation, α is the linear dose response component and α/β is the “alpha/beta ratio” which quantifies the dose response “curvature”:

(1) $$S=P\,E\times \text{exp}\,\left[-\alpha \times D-\alpha /\left(\frac{\alpha }{\beta }\right)\times D^{2}\right]$$

In this model, survival at relatively low doses (*D* < α/β) is dominated by parameter α, whereas survival at high doses (*D* > α/β) is dominated by parameter β. *E. dermatitidis* data over the studied dose range of 0–1.6 kGy did not exhibit any visible “downward curvature” on a logarithmic scale, suggesting that the contribution of the α/β parameter is negligible in this dose range. Indeed, preliminary fits of the model to these data showed that the quadratic term in Eq. (1) is not statistically distinguishable from zero. This finding allowed us to simplify the model by eliminating the parameter α/β and retaining only the α × *D* term.

Importantly, not all cells in an irradiated cell population may have the same radiosensitivity, for example due to differences between cells in cell cycle stage, age, nutrient status, epigenetics, oxygenation, *etc*. Models that take such heterogeneous radiosensitivity into account have been used to describe the survival of tumor cells during radiotherapy (e.g., Carlone et al., 2004; Shuryak et al., 2015). One simple example of such a model, which allows analytic calculations, employs the exponential distribution to describe the probability distribution of the linear dose response parameter among the cell population. This can be done as follows, where *P*(*u*) is the probability that the linear dose response parameter is equal to *u*, where the mean value of this parameter is α:

(2) $$P\,\left(u\right)=\left(\frac{1}{\alpha }\right)\times \text{exp}\,\left[-\frac{u}{\alpha }\right]$$

The mean of Eq. (2) is α, and the variance is α^2^.

Equations 1 and 2 can be combined to generate the following equation for the cell surviving fraction (*SF*) as function of radiation dose *D*:

(3) $$S\,F=\int_{0}^{\infty }P\,E\times \exp\left[-u\times D\right]\times P\,\left(u\right)\times d\,u=P\,E/\left(1+\alpha \times D\right)$$

This survival function (Eq. 3) was fitted to the data for *E. dermatitidis* WT and PKS strains using maximum likelihood. The log likelihood (*LL*) function was generated from the binomial distribution, using the observed number of colonies and the estimated number of plated cells for each experimental result as the inputs (“number of successes” and “number of trials,” respectively, in commonly used binomial terminology). Best-fit values for parameters *PE* and α were found by maximizing the *LL* using the sequential quadratic programming (SQP) algorithm in Maple 2019 software. Data for the WT and PKS strains were analyzed separately, but for each strain the data for different particle types were combined because of their similarity.

For each strain, fitting was performed 1000 times with randomly chosen initial parameter values, and the best result was recorded. This procedure was intended to maximize the probability of finding the global optimum rather than a local one. Model parameter uncertainties (95% confidence intervals, CIs) were estimated by generating 3000 synthetic data sets where the observed number of colonies and estimated number of plated cells for each experimental result were replaced by Poisson-distributed random numbers with the mean set to the corresponding value in the real data set. The model was fitted to each synthetic data set, and parameter values recorded. The 2.5th and 97.5th percentiles of the distribution of each parameter across synthetic data sets were used as estimates of 95% CI for this parameter.

### RNA Extraction and Sequencing

Additionally, control cultures, and cultures irradiated with the second highest doses (250 Gy α-particles, 840 Gy protons, and 500 Gy deuterons) were processed further for RNA extraction: 9 mL of 2 × concentrated YPD medium was added to each of the cell suspensions collected from irradiated dishes, and these tubes were incubated for 1 h at 30°C and shaking at 200 RPM to allow for recovery. After this final incubation, cell suspensions were collected by centrifugation (4000 × *g*, 5 m) and re-suspended in 1 mL of RNAlater (Thermo Fisher, MA, United States). RNAlater solutions were split into two tubes (500 μL each) and stored at RT for 2 days before pelleting through centrifugation (15000 × *g*, 1 m), removal of the supernatant, and storing them at −80°C until RNA extraction.

RNA was extracted from individual cell pellets with the RiboPure RNA Purification Kit for yeast (Thermo Fisher), and RNA quality was verified using a 2100 Bioanalyzer (Agilent, CA, United States) to confirm that RNA integrity numbers (RIN) were greater than 7.5. Two samples from each condition were then sent on dry ice and subjected to sequencing on an Illumina HiSeq4000 Sequencer by the Yale Center for Genome Analysis (YCGA, Yale University School of Medicine, West Haven, CT, United States). Verification with qRT-PCR and validation that two replicates was sufficient to identify many transcriptomic changes in response to IR were completed in a previous publication (Schultzhaus Z. et al., 2020). Data from this RNA-seq experiment is publicly available through the Gene Expression Omnibus using accession number GSE152116.

### RNA-Seq Data Analysis

To analyze gene expression changes in response to particle irradiation, raw FASTA files produced by the sequencing runs were downloaded from the YCGA. These files were subjected to alignment and quantification using Salmon 2.0 software (Patro et al., 2017), with the transcriptome and genome of *E. dermatitidis*, both assembled and characterized previously (Robertson et al., 2012; Chen et al., 2014; Schultzhaus Z. et al., 2020), used as a reference. Transcript abundance and quantification information provided through this analysis was analyzed using the DEseq2 package (Love et al., 2014) in RStudio (RStudio Team, 2015). Deuteron had an independent set of controls due to a different time of irradiation, as mentioned above, but was otherwise treated the same and it exhibited similar levels of correlation between biological replicates.

Further analysis to identify patterns within the expression of the RNAseq data were performed using several software packages. Venn diagrams were produced using Venny 2.1^1^ (Oliveros, 2007). Statistical analysis of enrichment of genes was performed using FungiFun 2^2^ (Priebe et al., 2011, 2014). Search settings in this case were adjusted to identify Gene Ontology terms under the category of Biological Process, with an FDR of <0.01.

EuKaryotic Orthologous Group (KOG) analysis of highly regulated genes from both particle and γ-regulated and was performed using information provided by previous publications and available at MycoCosm (Chen et al., 2014; Grigoriev et al., 2014). Genes were placed into the broadest KOG categories for presentation of the least convoluted depiction of the datasets.

Network analysis was performed to identify connected and similarly regulated genes using STRING software^3^ (Snel et al., 2000; Mering et al., 2003), with settings programmed to show only connections with the highest significance (confidence > 0.9). Data on the nodes identified through this search were downloaded, and the results presented in the manuscript were compiled to include the genes that had one or more connection to another upregulated gene with a confidence value of 0.999.

Motifs that were enriched among highly regulated genes were discovered using the MEME-suite^4^ (Bailey et al., 2009, Bailey et al., 2015) using the Multiple Em for Motif Elicitation (MEME) software for identification. Promoters for all genes regulated >5-fold and <5-fold were used as input. Shuffled input sequences, random promoter sequences, and random ORF sequences were used as controls to verify the identification of the enriched motif. The enrichment of the identified motif was then verified using Analysis of Motif Enrichment (AME), and the complete set of promoters was searched for this motif using the Find Individual Motif Occurrences (FIMO) program.

## Results

### Susceptibility of *E. dermatitidis* to Proton, Deuteron, and α-Particle Irradiation

**FIGURE 1:**
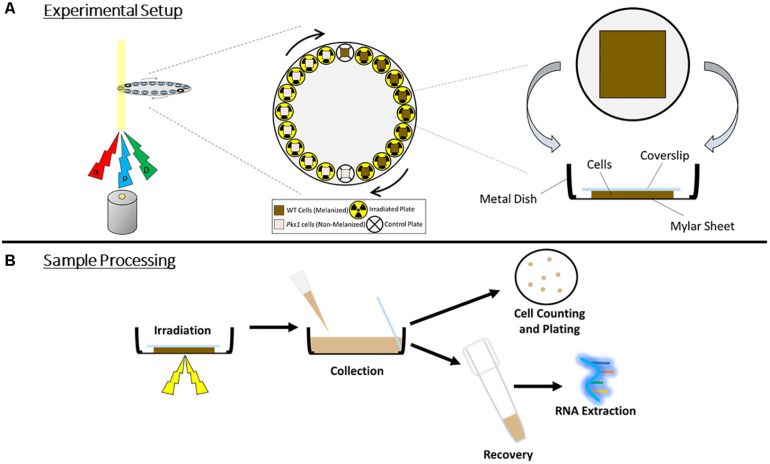
**(A)** Irradiation setup. Particles (α, alpha; p, proton; D, deuteron) were produced and passed through cell sample chambers placed along the circumference of a disk, which rotated automatically to provide each sample with an even exposure. For control samples, particle source was momentarily stopped as the cells passed through the beam. **(B)** Overview of sample processing during and post-irradiation, demonstrating the orientation of the cells and the coverslip within the chamber, and the harvesting of cells for survival measurements and RNA extraction.

We initiated this study by characterizing the resistance of *E. dermatitidis* melanized (WT) and non-melanized (Δ*pks*) strains to three ionizing particles with different RBE, LET, and mass [in ascending order: protons (p), deuterons (D), and α-particles (α) (Hall and Giaccia, 2006)]. To enable comparisons with γ-IR, we considered several factors (see Figure 1) (see section “Materials and Methods”), including: the lower penetration of these particles into materials; the RBE of each particle type in comparison with γ-IR (Fano, 1963; Nikjoo et al., 1999); and the shape of previously constructed survival curves exhibited by *E. dermatitidis* after irradiation. At doses much greater than 2000 Gray (Gy, J/kg), for example, survival decreases rapidly, and differences between strains become harder to interpret due to the low percentage of recovering colonies produced (Schultzhaus Z. et al., 2020). Effects (such as protection imparted by melanin) are therefore best detected at moderate exposures. Taking this into account, we chose to irradiate each strain with doses equivalent to 500, 1000, and 2000 Gy of γ-IR, similar to the doses we used to compare melanized and non-melanized strains in a previous publication (Schultzhaus Z. et al., 2020).

**FIGURE 2:**
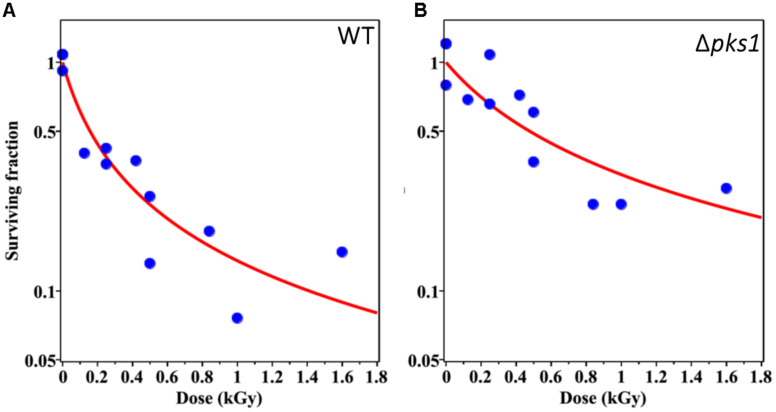
Survival of WT **(A)** and Δ*pks1* **(B)** cells after irradiation with ionizing particles of different LET. Blue circles represent experimentally determined data points from the survival assay and red lines indicate the most optimal (best-fit) survival curve for each strain, using maximum likelihood, when incorporating CFU data at each dose into the survival function (Eq. 3). Dose in kGy is provided on the *x*-axis.

After irradiation, a known quantity of cells from each sample was inoculated on separate plates. Survival was then modeled with the resulting measurements. The best-fit parameter values for the WT strain were: α = 6.38 (95% CI: 0.26, 16.51) kGy^–1^, *PE* (Plating Efficiency) = 0.60 (0.21, 0.99). Corresponding values for the Δ*pks* strain were: α = 2.10 (0.01, 10.04) kGy^–1^, *PE* = 0.32 (0.16, 0.80) (see the section “Materials and Methods” for explanation of modeling and Supplementary Tables 1, 2 for plating and modeling data). The fitted dose response curves and data points are shown in Figure 2. Notably, these differences between strains in terms of parameter values did not reach statistical significance (95% CIs overlapped). However, these results suggest some evidence of heterogeneous radiosensitivity in the irradiated fungal population. The Δ*pks* strain appears to have a smaller α parameter (see the section “Materials and Methods” for description of this parameter) than the WT strain, suggesting that Δ*pks* has lower radiosensitivity. PE, though, was also smaller for Δ*pks* than WT, which may suggest that only some selected “healthy” sub-population of Δ*pks* cells was able to grow even without radiation, and possibly this selection was correlated with improved radioresistance as well. Indeed, Δ*pks* cells have been shown to clump together, possibly due to a decreased ability to completely cleave apart after cell division, which can affect plating efficiency (Feng et al., 2001; Schultzhaus Z. et al., 2020). In all, though, there was no clear protection, or difference at all, in WT cells compared to those without melanin.

### The Transcriptomic Response of *E. dermatitidis* to Particle Irradiation

The effect of exposure on cell survival was roughly equivalent between these particles and γ-IR for similar doses (Schultzhaus Z. et al., 2020), but with knowledge of their varying LET characteristics, we hypothesized that differences between these conditions could be accessed by looking at the transcriptomic response for each source. Therefore, next, we performed RNA-seq on cultures recovering from the second highest dose (equivalent to 1000 Gy of γ-IR) for each particle, corresponding to 30–50% cell survival depending on the type of IR. Before collecting RNA we also incubated cells in fresh medium for 1 h to allow for them to mount a transcriptomic response (see “Materials and Methods”).

**FIGURE 3:**
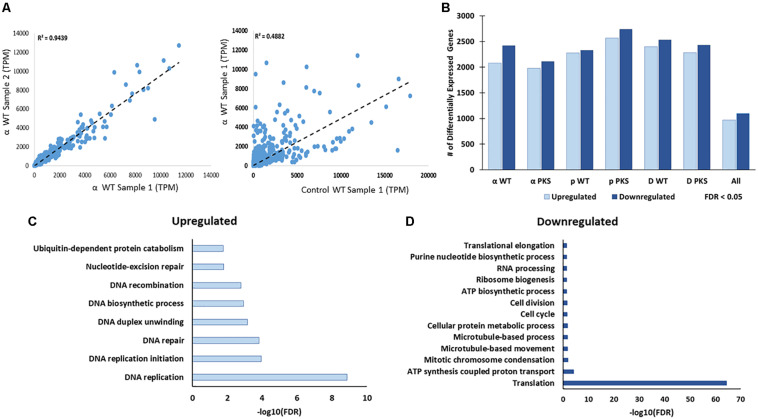
**(A)** Correlation between Transcripts Per Million (TPM) counts between biological replicate samples within the same condition, between different strains in the same condition, or within the same strain across different conditions. Here, and below, the strains included are the melanized WT and the non-melanized PKS strains, and the condition being exposure to the second-highest dose of particle IR, as detailed in the Section “Materials and Methods” (250 Gy α-particles, 840 Gy protons, and 500 Gy deuterons). **(B)** The number of genes differentially expressed, with a significance cutoff of FDR < 0.05, for each condition, as well as shared in all conditions. **(C)** Gene Ontology-Biological Process categories enriched with the shared, upregulated and **(D)** downregulated gene sets. FDR values provided by FungiFun 2.0. Higher absolute values of –log10(FDR) demonstrate greater significance.

Each particle irradiation induced a strong and consistent gene expression pattern in both strains (Supplementary Tables 3, 4). For example, *R*^2^ values from comparative analysis of transcripts per million counts ranged from 0.84 to 0.95 between biological replicate samples as well as WT and Δ*pks1* cultures under the same condition (IR exposure or control), while the irradiated transcriptome was vastly different from that of controls (Figure 3A, Supplementary Table 3, and Supplementary Figure 1). Initial analysis of differential gene expression with an FDR < 0.05 demonstrated that a range of 4088 (2110 up, 1978 down in the Δ*pks1* deuteron-irradiated culture) to 5305 (2739 up, 2566 down transcripts in the Δ*pks1* proton-irradiated culture) out of the 9268 transcripts predicted to be encoded by the *E. dermatitidis* genome (Chen et al., 2014) were differentially expressed. Overall, 1095 (upregulated) and 969 (downregulated) genes were seen in every sample at this cutoff level, representing 22.2% of the predicted protein-coding genes in this organism (Figure 3B and Supplementary Tables 3, 4).

We next looked within this shared set of genes for Gene Ontology (GO) categories that were significantly enriched (see the section “Materials and Methods”). This resulted in the following observation (Figures 3C,D): genes involved in DNA repair (e.g., DNA replication, DNA recombination, and nucleotide-excision repair) and genes involved in protein catabolism (e.g., ubiquitination) were overrepresented among the upregulated genes (Figure 3C). Approximately one third of the genes in the upregulated group (369/1095, 33.7%), moreover, were predicted to encode proteins with no known function.

Among the downregulated genes (Figure 3D), biological processes that were significantly enriched were involved in cell growth, including categories such as intracellular trafficking (microtubule-based movement), ATP synthase-coupled proton transport, and translation (ribosomal biogenesis) were particularly enriched. Once again, one third of the genes (340/969, 35.1%) encoded hypothetical proteins (Supplementary Tables 3, 4). This is approximately half as numerous as the proportion of unannotated genes within the entire genome (5842/9578, 60.1%), which suggests that this set is enriched with genes involved in well-characterized processes. However, hundreds of genes still remain to be characterized in this organism before a full picture of this response is known. Taken together, these results suggested that *E. dermatitidis* cells respond to charged particle irradiation by activating the DNA repair machinery and removing damaged proteins, as well as inhibiting cell growth.

### Comparison of the Transcriptomic Responses of *E. dermatitidis* to Particle and γ-Irradiation

**FIGURE 4:**
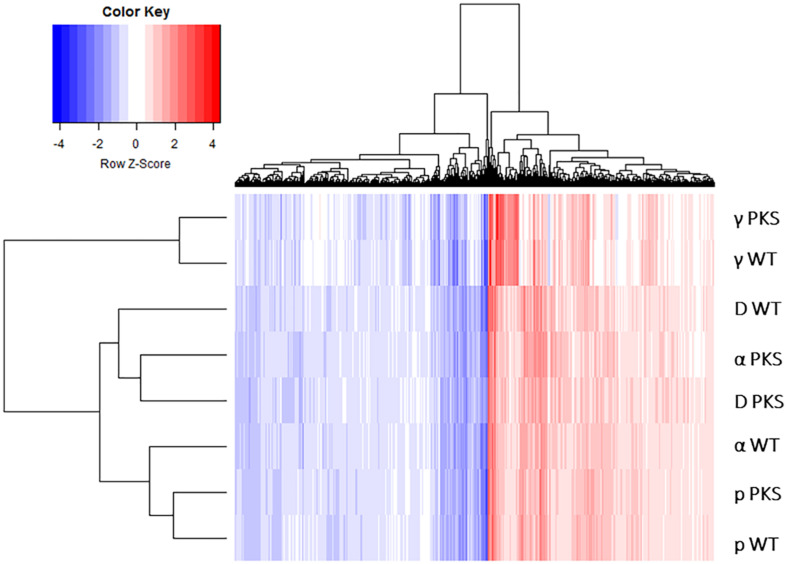
Heatmap depicting the log_2_-fold change values of genes within the six particle and the two γ-IR-responding datasets. Genes evaluated were those that were found to be differentially expressed (with an FDR value < 0.05) in all of the six particle datasets.

This response was similar to the γ-radiation response (Schultzhaus Z. et al., 2020), something which could be seen at a larger level by plotting the log_2_fold-change values of genes significantly regulated by all IR sources on a heatmap (Figure 4). However, the heatmap produced by this analysis showed that samples also clearly clustered by IR type (particle and γ-ray), demonstrating that there were notable differences between the two responses that needed further analysis to uncover.

**FIGURE 5:**
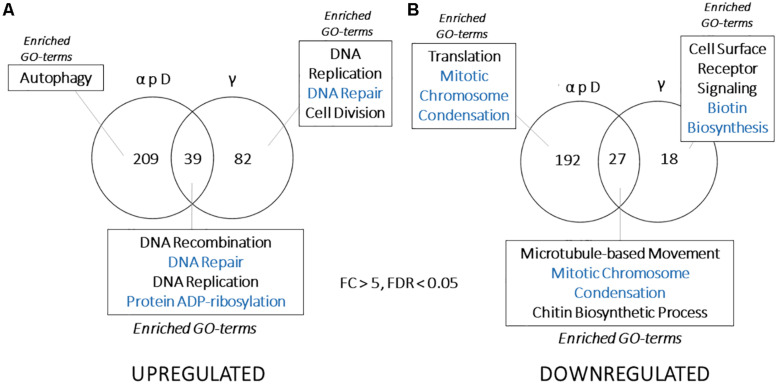
**(A)** Venn diagram showing overlap between genes that are highly upregulated or **(B)** downregulated (FDR < 0.05, >5 or <–5-fold change in expression) in particle or γ-induced datasets. Categories presented for each section include Gene Ontology-Biological Process categories enriched (FDR < 0.01, from FungiFun 2.0) for each group of genes.

Therefore, we focused on getting more information about the genes that were differentially expressed in each group, particularly those that did not have informative annotations, and compiled lists of genes fitting this description that were also differentially regulated more than five-fold (FDR < 0.05) in either γ-radiation or particle – datasets for comparison (Schultzhaus Z. et al., 2020). Notably, we observed less overlap among these two sets than we expected, with only approximately 12% of the genes shared overall (Figures 5A,B). We also were surprised to see that, even after compiling the results from six separate experiments (WT and Δ*pks* cells for three particle exposures), there were many more genes that passed the >5-fold change cutoff in the particle dataset compared with the γ-irradiated dataset (*N* = 467 vs. 166, respectively), indicating that particle irradiation invoked a more dramatic response in the transcriptome.

The lack of overlap between these gene sets caused us to reanalyze these data for patterns of enrichment that were specific to each group. This revealed some interesting features. First, DNA repair genes were still enriched among the shared, upregulated set, but DNA replication and repair genes were also enriched within the γ-specific, upregulated group. These proteins included Rad54B, the NHEJ protein Ku70, DNA damage-binding protein CMR1, as well as DNA polymerase α and ε, replication fork protection complex subunit Swi1/Tof1, and the DNA replication regulator SLD2 (Figure 5A). Proteins only upregulated by particle irradiation, on the other hand, included autophagy-related proteins (HMPREF1120_02756/ATG8, HMPREF1120_07182/ATG3, and the cysteine protease HMPREF1120_06308/ATG4) and specialized genes potentially involved in sensing and signaling, such as bacteriorhodopsin (HMPREF1120_00264) and a Sir2-like histone deacetylase (HMPREF1120_05820).

Additionally, the group of shared, downregulated genes was small (*N* = 27), and was enriched for proteins involved in cell growth, cell cycle, and intracellular trafficking, but not enriched for those associated with translation and ribosomal structure (Figure 5B). An even smaller set of genes (*N* = 18) were uniquely downregulated by γ-radiation. On the other hand, translation was highly enriched among the downregulated genes specific to the particle-irradiated gene set. This is notable for two reasons - the antagonistic relationship between protein synthesis and autophagy (Beau et al., 2008; Kraft et al., 2008; Wyttenbach et al., 2008; Hands et al., 2009), and the observation that proteins involved with translation were downregulated by γ-radiation, just not to the extent to pass the cutoff here. Particle irradiation, therefore, induces a similar, but far stronger repression of translation-associated genes than γ-radiation (Schultzhaus Z. et al., 2020).

**FIGURE 6:**
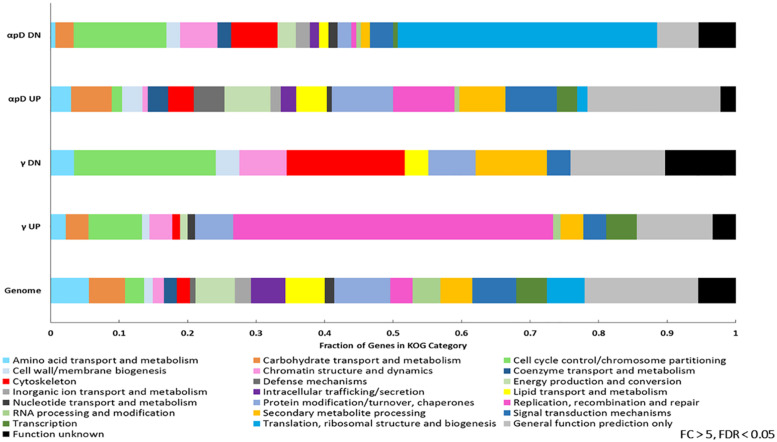
KOG categories identified among the genes differentially expressed >5 or <–5-fold in the particle (α-, proton, or deuteron) or γ-induced datasets, as well as the whole *E. dermatitidis* genome.

After this analysis many genes still remained without a predicted function, so we further examined each set with information from the KOG database. Grouping genes into 22 of the KOG categories represented in a substantially decreased the number for which no information was known (from 188/467 to 46/467 for particle irradiation, and from 52/166 to 20/166 for γ-irradiation), and also allowed us to look at the functional composition of the entire *E. dermatitidis* genome. Figure 6 shows these results. It is immediately apparent that particle-regulated genes embody a greater functional diversity compared to γ-regulated genes. Upregulated genes from the particle irradiation sets include a high proportion annotated as involved in defense mechanisms (e.g., multidrug transporters and general stress-response proteins) while the downregulated gene set from this experiment had a large proportion involved in amino acid transport and chromatin structure compared with the whole genome. The upregulated genes in the γ-IR experiment, however, were dominated by those involved in Replication, Recombination, and Repair, while among downregulated genes there appeared to be an enrichment of those involved in secondary metabolism (Figure 6).

In all, although the transcriptomic response to IR broadly involves upregulation of DNA repair and attenuation of growth and protein production, there are some differences observed in the response to particle irradiation, including an upregulation of genes regulating cell death and a stronger downregulation of translation-associated genes.

### Genes Most Sensitive to Particle Irradiation Exposure

One goal of this study is to identify genetic loci that robustly respond to IR, which could be used to develop strains that can sense IR and produce various outputs tied to gene expression. With this in mind, we searched for the most promising loci using the shared particle gene through MEME analysis, which searches for conserved promoter sequences in gene sets; STRING analysis, which identifies connections between genes within datasets based on published genetic studies; and identification of the genes that showed the strongest change in expression during IR recovery. The output of MEME and STRING analyses are presented in Supplementary Tables 5–8 and Supplementary Figure 2. Briefly, MEME analysis identified a possible 9-nucleotide sequence marking IR-regulated genes, although it was clear that other unidentified elements also exist (Supplementary Tables 5, 6). STRING analysis further confirmed the centrality of DNA replication among the upregulated genes, but also identified certain genes that were central to this pathway, such as the Minichromosome Maintenance Protein complex (Supplementary Figure 2 and Supplementary Tables 7, 8).

Next, to identify the most prominent among the differentially expressed genes, we averaged expression data across all strains and particle samples. We identified 19 genes that were differentially expressed with a log_2_FC > 5, representing a >32-fold change in expression during recovery from IR exposure (Table 1). The most upregulated gene (log_2_FC = 6.44 ± 0.77) among all samples was HMPREF1120_01375. It is annotated as a triacylglycerol (TAG) lipase and contains acetyl esterase and lipase domains, potentially associating it with fatty acid catabolism in autophagy (Cingolani and Czaja, 2016; Jaishy and Abel, 2016). Other upregulated genes in this list include HMPREF1120_08154, a homolog of *Saccharomyces cerevisiae* YTA12, a component of the mitochondrial inner membrane mAAA-protease that could be involved in clearing damaged proteins; HMPREF1120_02698, a homolog of *S. cerevisiae* UBP15, which is involved in S-phase entry (Ostapenko et al., 2015); and HMPREF1120_02127, a homolog of *S. cerevisiae* gene YGL039W, a protein involved in reducing aldehydes, which are present in cells undergoing oxidative stress (Singh et al., 2013). Interestingly, HMPREF1120_08448, HMPREF1120_00007, and HMPREF1120_06618 did not contain any predicted functional domains, and we were unable to find any orthologs of these genes in genomes from species outside of the *Exophiala* genus.

**TABLE 1:** Genes that were differentially expressed to a level >30-fold or <−30-fold in all particle conditions, including information about their function, conservation, and the average induction or repression levels.

All sources	Gene	Uniprot annotation	Conserved domains	Avg. log_2_FC	SD
Up	HMPREF1120_01375 HMPREF1120_08154	Triacylglycerol lipase AAA family ATPase	Acetyl esterase/lipase SpoVK/Ycf46/Vps4 family (cell wall/membrane biogenesis, cell cycle control)	6.44 6.20	0.77 0.73
	HMPREF1120_02698	Ubiquitin thioloesterase	Ubiquitin carboxyl-terminal hydrolase; zinc finger; PIP-kinase	5.84	0.68
	HMPREF1120_08138	Uncharacterized protein	Ankyrin repeat domains	5.84	0.85
	HMPREF1120_08448	Uncharacterized protein	–	5.60	1.84
	HMPREF1120_08742	Dimethylaniline monooxygenase (*N*-oxide forming)	Flavin-containing monooxygenase	5.39	0.64
	HMPREF1120_00007	Uncharacterized protein	–	5.28	0.93
	HMPREF1120_02578	Protein YOP1	TB2/DP1 (deleted in polyposis), HVA22 (abscisic acid-induced) protein family	5.22	0.68
	HMPREF1120_02127	Cinnamyl-alcohol dehydrogenase	Short-chain dehydrogenases/reductases	5.04	0.91
	HMPREF1120_06618	Uncharacterized protein	–	5.02	0.68
Down	HMPREF1120_07157 HMPREF1120_06748	Structural maintenance of chromosomes protein Aurora kinase, other	SMC superfamily, chromosome segregation ATPase STKC Aurora kinase superfamily (chromosome segregation)	−5.05 −5.18	0.88 0.78
	HMPREF1120_01579	Condensin complex subunit 3	Chromosome condensation complex	−5.32	1.26
	HMPREF1120_07981	Chitin synthase 1	Chitin synthase: classes I, II, and III	−5.34	0.86
	HMPREF1120_04574	Uncharacterized protein	–	−5.64	1.02
	HMPREF1120_03688	Uncharacterized protein	Chromosome segregation subunit of the Nuf2-Ndc80 complex	−5.72	0.78
	HMPREF1120_03067	Uncharacterized protein	–	−5.77	0.65
	HMPREF1120_08344	Uncharacterized protein	–	−5.78	0.73
	HMPREF1120_04843	Endoglucanase	Cellulase, glycosyl hydrolase, glucanase	−5.85	1.13
					

Within the group of shared downregulated genes, three genes had no conserved domains or informative orthologs in other species (HMPREF1120_04574, HMPREF1120_03067, HMPREF1120_08344). Of the other six, however, four had functions related to known IR responses, being involved in chromosome dynamics in some way, such as the orthologs of SMC1 and SPC25 (chromosome segregation), the aurora kinase IPL1, and the condensin YCG1. Additionally, the chitin synthase CHS1 and an endoglucanase were the remaining two genes that were on this list of genes repressed during IR exposure recovery. These could be associated with the cessation of growth that we have observed taking place in *E. dermatitidis* cells while damage is repaired or cleared (Schultzhaus Z. et al., 2020). Importantly, 16/19 of these genes were regulated similarly in our γ-regulated dataset, so further studies of IR sensitive promoters may be able to focus on the expression of these few genes in different environments.

### Particle-Specific Transcriptomic Responses

**FIGURE 7:**
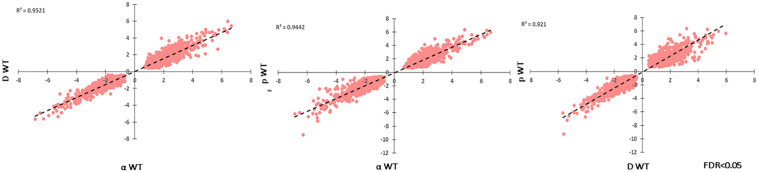
Correlation of log_2_FC-values for each significantly regulated gene present in all WT, particle-irradiated gene sets, demonstrating high concordance in the response to the three sources.

**FIGURE 8:**
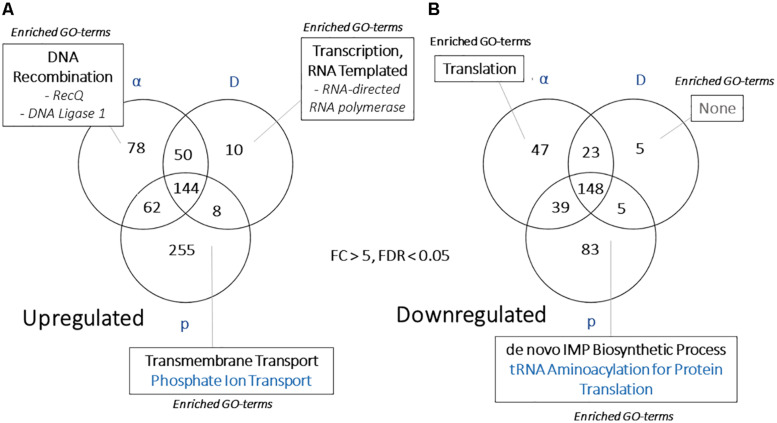
**(A)** Venn diagram demonstrating the overlap in highly regulated [>5 or **(B)** <–5-fold] genes in each WT, particle-responsive dataset. Categories presented for each section include Gene Ontology-Biological Process categories enriched (FDR < 0.01, from FungiFun 2.0) for each group of genes.

Another goal of this study was to identify unique responses to different ionizing particles, so next we looked for patterns specific to certain irradiation datasets. Such loci were not immediately apparent, as the datasets were similar. This is demonstrated by comparing FC values for each significantly regulated gene (FDR < 0.05) shared among the particle groups, which produced *R*^2^ values >0.92 for all pairwise comparisons (Figure 7). Moreover, no genes were observed to be upregulated under one condition and downregulated in another. When these gene groups were minimized to facilitate closer analysis by looking only at genes differentially expressed in each condition with a FC > 5, however, several genes were identified in only one set, and some of these unique gene sets were significantly enriched for certain biological processes (Figures 8A,B). For α-particle irradiated samples, genes involved in DNA repair, including DNA Ligase 1 and the helicase RecQ, were upregulated, while genes predicted to be involved in translation were specifically enriched within the α-downregulated genes. Genes upregulated only after proton irradiation were enriched in transmembrane transport, including 13 Major Facilitator Superfamily domain-containing proteins, while genes involved in IMP biosynthesis (purine metabolism) and tRNA aminoacylation were overrepresented among downregulated genes. It is worth noting that protons and α-particles represent the two extremes of particle size, which may account for the higher number of uniquely regulated genes in these two datasets. Finally, within the deuteron set, genes involved in transcriptional regulation were enriched among the upregulated transcripts, including RNA-dependent RNA polymerase, which is thought to be involved in RNA silencing.

To search for more subtle patterns within this data, we again performed STRING analysis to identify pathways that were specific to a certain particle using genes with highly confident links to at least two other genes (probability > 0.9) among all significantly upregulated genes (FDR < 0.05) for each condition (Supplementary Tables 9, 10). This provided a detailed list of DNA repair and protein catabolism components induced during the response to particle exposure, including the polymerases involved in DNA replication (α, δ, and ε), the proteins involved in regulating the cell cycle (Cdc6, 45, and 48), and those involved in DNA repair (for HR – Rad50, 51, 52, MRE11, and Rhp54; for UV excision repair – Rad32; for mismatch repair – MLH1/MSH3/PMS2; and translesion synthesis – Rev1). It also revealed proteins involved in transcriptional regulation, including two histone acetyltransferase-associated categories, the histone chaperone ASF1, and a Jumonji domain-containing protein, which are involved in transcriptional repression through modulating histone methylation (Smith et al., 2006). Finally, it identified other distinct pathways not directly related to DNA or protein damage, including the Myosin ATPase, involved in intracellular trafficking, and nitrogen (glutamine synthase and glutamate dehydrogenase) and carbon (phosphoenolpyruvate carboxykinase) metabolism gene nodes.

Supplementary Tables 11, 12 present the genes represented as highly confident nodes present in only one or two conditions. This analysis provided the most detailed picture of the different ways each source affects *E. dermatitidis*. For example, 18 different categories had two or more genes that were related with high confidence between the α- and deuteron-irradiated samples, which included two unique categories of DNA-associated proteins: the RecQ repair protein and the cohesin complex. In the proton and deuteron datasets, moreover, DNA topoisomerase (DNA replication), the FACT complex (chromatin structure), the RNA helicase (splicing), and Tyrosinase (melanin production) nodes were present only in deuteron and proton datasets. Even more nodes were discovered that were unique to individual irradiation sources. α-particle samples had subunits of the actin-related protein 2/3 complex [which is involved in endocytosis and morphogenesis (Commer and Shaw, 2020)] and synaptobrevin, which is involved in intercellular trafficking. Similarly, the deuteron set contained proteins linked to fimbrin, which is involved in actin cytoskeleton regulation, endocytosis, and morphogenesis (Upadhyay and Shaw, 2008), and the MYST family of histone acetyltransferases. The proton data set had the most unique categories in this analysis (*N* = 30), and included interesting links such as cystathionine synthase and glutaredoxin genes, which may be involved in the response to increased ROS levels, and several genes involved in mRNA splicing, including the splicing factors *slt11* and *ini1*. This may warrant further exploration of alternative splicing and isoform analysis of *E. dermatitidis* cells recovering from specific sources of IR.

Lastly, we attempted to discover distinct genes that responded to one specific source, which was more challenging. In all, only 15 genes were upregulated >10-fold in response to one type of particle. Of these genes, moreover, only three had predicted functions (Table 2). Proton irradiation again had a larger set of genes than α-particle or deuteron irradiation, of which one interesting member was the receptor for Mating Pheromone A, but the overall pattern still held with this sample, with 9/11 genes having no identifiable functional domains. This lack of highly regulated loci unique to any given particle, particularly in the context of the vast transcriptomic changes taking place during the IR exposure response, reiterates that there is substantial overlap in the type of cellular damage that occurs after acute exposure to each of these IR sources, and that much more detailed work is necessary to tease the different effects apart.

**TABLE 2:** Genes specifically expressed in response to unique particle irradiation sources, including information on function, conservation, and expression.

**IR source**	**Gene**	**Uniprot annotation**	**Conserved domains**	**Log_2_FC**	**SD**	**FDR**
α	HMPREF1120_06214	Uncharacterized protein	–	4.30	1.2	0.013
	HMPREF1120_04615	Uncharacterized protein	Broad specificity phosphatase PhoE	3.36	1.24	0.004
	HMPREF1120_06237	Uncharacterized protein	–	3.44	0.776	0.05
D	HMPREF1120_01524	Uncharacterized protein	–	3.42	1.30	0.007
p	HMPREF1120_03390	Uncharacterized protein	–	5.46	0.117	0.027
	HMPREF1120_05909	Uncharacterized protein		4.88	0.797	0.044
	HMPREF1120_07028	Uncharacterized protein	–	3.71	0.4	1.14e-06
	HMPREF1120_06554	Uncharacterized protein	–	3.62	0.369	3.33e-06
	HMPREF1120_01962	Uncharacterized protein	–	4.42	0.93	1.26e-04
	HMPREF1120_08985	Gluconate 5-dehydrogenase	Short-chain dehydrogenases/reductases	3.42	0.128	0.006
	HMPREF1120_01617	Uncharacterized protein	–	3.38	0.348	6.97e-08
	HMPREF1120_03162	Uncharacterized protein	–	3.32	1.45	0.013
	HMPREF1120_04175	Uncharacterized protein	–	3.45	0.365	1.68e-04
	HMPREF1120_05389	Uncharacterized protein	–	4.34	2.04	0.009
	HMPREF1120_08176	Pheromone A receptor protein	STE3 superfamily/mating pheromone A receptor	3.4	0.991	4.66e-10

### Effect of Melanin on the Response to Charged Particle Radiation Exposure

**FIGURE 9:**
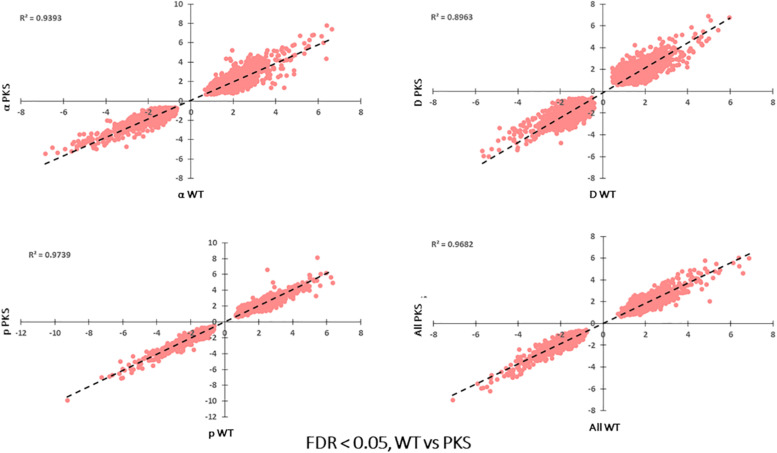
Correlation of log_2_FC-values for each significantly regulated gene present WT vs. Δ*pks1*, particle-irradiated gene sets, demonstrating high concordance between the two strains in the response to IR.

**FIGURE 10:**
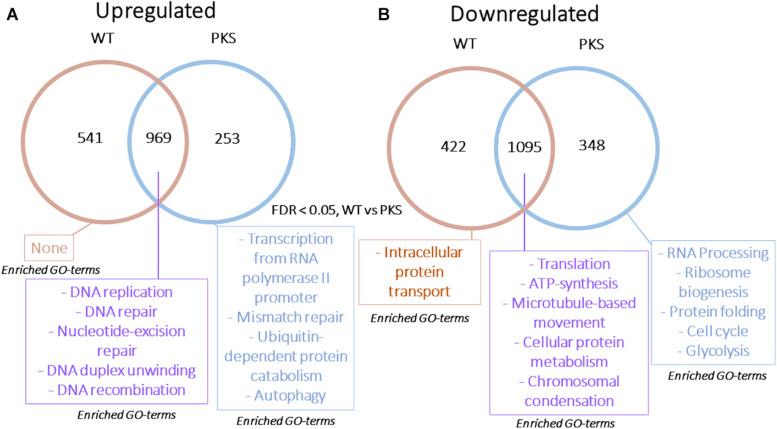
**(A)** Venn diagram demonstrating the overlap in upregulated or **(B)** downregulated genes in WT and Δ*pks1* particle-responsive datasets. Categories presented for each section include Gene Ontology-Biological Process categories enriched (FDR < 0.01, from FungiFun 2.0) for each group of genes.

The final analyses we performed were a comparison of the responses of melanized and non-melanized cultures to these particle sources. Because the particle-associated transcriptome was distinct from our previous γ-radiation study, and particles are less penetrating, we were prepared to see some difference between the two strains. However, in agreement with the survival data we collected, the responses had substantial overlap, with comparative plots of Log FC between each sample exhibiting *R*^2^ values of ∼0.9 and greater with no genes significantly regulated in opposite directions between any samples (Figure 9), and no differences in highly confident STRING nodes (data not shown). However, we did observe some broad differences between the two responses. Specifically, non-melanized cultures had more enriched biological processes compared to the melanized gene set, even though the former contained a smaller group of genes (Figure 10). Enriched categories from this analysis included transcription, mismatch repair, protein catabolism, and autophagy in the upregulated, non-melanized set, while additional genes involved in translation (protein folding, ribosomal biogenesis, RNA processing) and growth and energy production (cell cycle, glycolysis) were enriched among the downregulated genes unique to the non-melanized strain. Interestingly, we have observed several other times that the regulation of translation is affected by melanization status, with ribosomal genes preferentially downregulated, under normal conditions, in melanized strains (Robertson et al., 2012; Schultzhaus Z. et al., 2020). These enrichment analyses provide some further credence to the idea that a lack of melanin mediates a complex response in *E. dermatitidis*, but the response is subtle and ancillary to IR recovery.

## Discussion

In this study, we obtained a detailed characterization of the *E. dermatitidis* IR response by performing transcriptomic analysis of melanized and non-melanized cultures after exposure to three different types of ionizing particles. Previously, such an approach has been somewhat contentious. The importance of repair gene induction for cell recovery after irradiation, for example has been challenged (Dornfeld and Livingston, 1991; Birrell et al., 2002). However, advances in bioinformatic analysis and transcriptome profiling have demonstrated that such studies actually can at least provide great information about what IR does to cells. For example, a microarray experiment in *C. neoformans* allowed investigators to determine that ergosterol levels affected IR sensitivity, and while it also lead them to a supposed “dead end” in looking at the importance of catalase genes, the extensive upregulation of redox genes indicated that the cells were responding to oxidative stress (Jung et al., 2016). In our case, we saw the upregulation of nearly all types of DNA repair pathways. Not all of these pathways will be essential for resistance to acute IR exposure if they are deleted, as is the case for the NHEJ pathway in *E. dermatitidis*, which is dispensable for γ-ray resistance (Schultzhaus Z. et al., 2020), but the expression patterns of these genes still allows for an understanding of what is occurring in this organism as a result of the exposure, which is the first step toward augmenting resistance, a central goal of radiobiology.

In our prior study on the transcriptomic response of *E. dermatitidis* to γ-radiation, we came to three basic conclusions (Schultzhaus Z. et al., 2020). First, there is little difference in how melanized and non-melanized *E. dermatitidis* strains respond to IR. Each exhibited a similar survival rate, and each demonstrated an almost complete overlap in their gene expression levels during recovery. Second, recovery from γ-radiation in *E. dermatitidis* is dominated by the induction of DNA repair pathways and the repression of translation, the latter of which is likely indicative of cell cycle arrest that is coupled to DNA damage sensing (Weinert and Hartwell, 1988). Third, that species-specific and poorly characterized proteins also have the potential to play a large part in recovery from IR exposure. Almost half of the transcripts responding to IR, in fact, were annotated as hypothetical proteins, and many of the most highly induced transcripts encoded proteins not conserved outside of the *Exophiala*. One of these was in fact found to be essential for IR resistance (Schultzhaus Z. et al., 2020).

We expand upon each of these conclusions with the present study. We reasoned that irradiation by particles, rather than photons, could interact with melanin in a different way, due to the more compact tracks that these particle make through cells (Dadachova et al., 2008; Schweitzer et al., 2009; Upadhyay et al., 2016). However, we once again observed no significant change in survival or gene expression after exposure to any particle source in the absence of melanin. This further supports the conclusion that IR exposure induces a strong and consistent response from the transcriptome that is independent of the presence of melanin, and that melanin does not play a large role in the resistance of this organism to acute IR exposure under the conditions of extremely high, rapid doses that we have used in this study.

It is important to note, however, that many aspects of cellular melanization, including extent, localization, and chemical nature may be affected by the environment, and in turn this may affect how impactful melanin is in mediating the IR recovery response. Using cells with greater amounts of melanin, or changing the irradiation dose rate, could unveil the protective effect seen in some species (Pacelli et al., 2017a). Moreover, there is an intimate relationship between the components of the cell wall (e.g., carbohydrates such as chitin and glucan, as well as lipids) and melanin, which uses the cell wall as a scaffolding to establish a higher-order structure (Bowman and Free, 2006; Camacho et al., 2017; Chrissian et al., 2020a,b) with emergent properties such as increased free-radical scavenging (Dadachova et al., 2008). This makes the presence of genes involved in cell wall biosynthesis (chitin synthase 1 and glucanase), as well as the TAG lipase especially conspicuous as members of the most differentially expressed transcripts. It is certainly possible that changes inadvertently occur to the protective, strengthening melanin shell around the cell due to this gene regulation after exposure, and this is something that should be explored carefully in the future. There are also some physiological aspects of melanization that need to be studied: we observed a strong transcriptomic response to melanization in *C. neoformans* (Schultzhaus et al., 2019), and here nearly 1000 unique proteins were differentially expressed only in melanized cells in response to particle exposure. These genes represent candidates to concentrate on to determine how melanin interacts with IR, but the observation that they are not enriched for any particular biological function underscores the complex effects melanin has in fungi (Poyntner et al., 2018), something that will take substantial effort to understand.

While the role melanin plays in the *E. dermatitidis* IR response is still obscure, the differences in how photonic and particle irradiation affect this organism have become clearer with this study. The observations we made after breaking down the “hierarchy” of differentially expressed genes in the two groups demonstrates the value of comparative transcriptomic experiments. For example, although all of the canonical processes involved in DNA repair (e.g., DNA synthesis and HR) were observed to be part of the recovery to both types of IR source, the response to particle irradiation involved a more diverse set of genes and an increased magnitude of differential gene expression- 467 were genes differentially expressed with a >5-fold change in every particle-irradiated sample, while this number was only 166 for γ-radiation. Beside being larger in number, the particle-associated genes encompassed a greater diversity of KOG groups, including upregulated genes associated with defense mechanisms (e.g., transporters), lipid and coenzyme transport and metabolism, and energy production and conversion, while there was a conspicuously strong enrichment of ribosomal subunits among the genes that were downregulated after particle exposure. This latter observation was interesting to us, because although we also saw downregulation of translation-associated genes in cultures responding to γ-radiation (Schultzhaus Z. et al., 2020), these were entirely excluded from the list of genes down-regulated five-fold or more. It is possible, then, that the damage that occurs in cells irradiated by ionizing particles requires a stronger cessation of protein synthesis, which may in turn result in massive changes in the proteomic response to these sources, something which we will focus on in follow-up studies.

Another insight we gleaned from the current dataset are the possible roles of autophagy and protein catabolism in recovery from particle irradiation. While DNA is considered to be the prime target for IR-induced damage, and genome instability the major cause of IR-induced cell death, protein oxidation is a known effect of IR-induced free-radical production [for detailed discussions on this topic, see Cabiscol et al. (2000), Braunstein et al. (2009), Daly (2012), Höhn et al. (2013), Radman (2016), Romsdahl et al. (2020), Schultzhaus Z. S. et al. (2020)]. Effects of IR on lipids, including changes in the levels of phospholipids and sterols in the plasma membrane, disruption of regulated membrane transport, and general lipid peroxidation are also known, though to a lesser extent (Stark, 1991; Kiang et al., 2012; Laiakis et al., 2014). Ergosterol biosynthetic genes are involved in IR resistance in *C. neoformans* (Jung et al., 2016, 2017), for example, and membrane permeability appears to be affected in the presence of low dose γ-radiation in *E. dermatitidis* (Robertson et al., 2012). Whether cells are able to deal with this additional, non-genetic damage, or if it also has the potential to render cells incapable of further growth, is unclear. Some studies have demonstrated that proteins involved in the oxidative stress response, such as catalases and superoxide dismutases, play only a minor role in IR resistance, likely because they themselves are damaged rapidly by the oxidative damage that occurs upon exposure (Daly, 2009, 2012; Sharma et al., 2017; Shuryak et al., 2017). In this case, proteins are either protected by the current redox state of the cytoplasm upon irradiation, or they are all damaged and the cell dies. On the other hand, autophagy, which allows cells to turnover damaged macromolecules, may affect IR resistance in eukaryotic cells (Lomonaco et al., 2009; Chaachouay et al., 2011; Höhn et al., 2013). In *S. cerevisiae*, a specific pathway of autophagy was recently found to contribute to DNA repair, and one of the genes involved in that study (ATG8) was also shown to be highly induced here (Eapen et al., 2017). The gene most highly induced by all particle exposures, moreover, was a TAG lipase. In fungi, these enzymes are generally secreted, and are important for modification and breakdown of extracellular lipids during colonization and infection (Gaillardin, 2010; Singh and Mukhopadhyay, 2012; Park et al., 2013). However, the TAG lipase induced here (HMPREF1120_01375) does not possess a signal peptide, so it may be involved in breaking down or otherwise processing intracellular lipid stores that are damaged during irradiation, a process that occurs during autophagy (Christian et al., 2013; van Zutphen et al., 2014). Few studies have looked at autophagy in fungi other than *S. cerevisiae*, but the process is well-characterized in humans, so this particle-associated response should be investigated in further studies.

The accumulation of more RNAseq datasets is, therefore, a useful and simple method for furthering our understanding of radiobiology, especially in organisms with small genomes such as fungi. We anticipate that next, it will begin to help with teasing out differences in the IR response between organisms that vary innate radiation resistance. This has been done, to some extent, in bacteria, where both the extremotolerant *D. radiodurans* and the susceptible *Shewanella oneidensis* have had their radiation-responsive transcriptomes profiled. In this case, distinct mechanisms for dealing with oxidative stress in these organisms were observed (Qiu et al., 2006). Less comparative analysis has been done in fungi, but a recent study has reported on the transcriptional response to both heavy-ion (carbon) beam and X-ray irradiation in *S. cerevisiae* (Guo et al., 2020), which provides a good initial step, as *S. cerevisiae* is somewhat more susceptible than *E. dermatitidis* to IR. In that study, DNA repair pathways once again dominated the upregulated gene set, and those involved in translation were highly enriched among the downregulated genes. Unlike the data presented above, however, genes involved in the response to oxidative stress were also induced, while autophagy and protein ubiquitination-associated genes were not. The overall number of differentially regulated genes in response to high LET IR was also much smaller for *S. cerevisiae* than here for *E. dermatitidis* (*N* = 773 vs. >4000 for each condition in the current study). These changes could be affected by the specific conditions used in each study, but that does not disallow them from pointing toward either a more robust or poignant response underlying the higher resistance in *E. dermatitidis*, and at least should direct further studies focused on understanding IR damage in these organisms using more standardized methods.

## Conclusion

In this study, we have concluded that the transcriptome-wide response to particle irradiation is unique from that of γ-irradiation in the radioresistant yeast *E. dermatitidis*. Specific changes included an induction of autophagy-related genes, a more prominent inhibition of translation, and the identification of hundreds of potentially novel proteins involved in the response to particle exposure. Melanin was found to have little effect on this response, and in fact, the non-melanized mutant was somewhat more resistant to certain doses of radiation exposure. Finally, some loci were identified that consistently respond to IR, which could be used for the future development of IR sensing constructs and strains. These results add to the understanding of how high and low LET radiation affects eukaryotic cells differently and provides a guideline for future transcriptomic and genetic studies on the IR response in fungi.

## Data Availability Statement

The datasets presented in this study can be found in online repositories. The names of the repository/repositories and accession number(s) can be found below: https://www.ncbi.nlm.nih.gov/genbank/, GSE152116.

## Author Contributions

ZS designed and performed the experiments and wrote the manuscript. AC designed and performed the experiments. IS assisted with experiments and analysis of data. ZW designed the experiments, obtained funding, and edited the manuscript. All authors contributed to the article and approved the submitted version.

## Conflict of Interest

The authors declare that the research was conducted in the absence of any commercial or financial relationships that could be construed as a potential conflict of interest.

## Footnotes

1 https://bioinfogp.cnb.csic.es/tools/venny/

2 https://elbe.hki-jena.de/fungifun/

3 https://string-db.org/

4 https://meme-suite.org

## Supplementary Material

Supplementary Figure 1 **(A)** TPM correlation plots within deuteron, **(B)** proton, and **(C)**α-particle irradiated cultures, within biological replicates and between samples.

Supplementary Figure 2 STRING network output showing highly confident connections between upregulated genes within shared, upregulated particle genes.

Supplementary Table 1 Colony forming unit and plating efficiency measurements for melanized (WT) and non-melanized (PKS) strains upon irradiation by each particle type.

Supplementary Table 2 Best-fit models for each strain detailing parameters for particle irradiation survival curves.

Supplementary Table 3 Complete output of Salmon and DESeq2 for the particle RNA-seq experiment. Each sheet is labeled with the comparison presented, and within each sheet is the differential expression analysis output, followed by the transcript quantification and length information for both biological replicates of that condition.

Supplementary Table 4 Genes differentially expressed after irradiation by all particles with fold change values >5 or <−5. Averages of all conditions are also shown, along with the standard deviation.

Supplementary Table 5 Raw output from MEME analysis of particle-regulated promoters (>5 or <−5-fold) that identified enriched motif associated with particle-responsive genes.

Supplementary Table 6 Sheet 1: FIMO analysis output from the MEME-Suite, which shows genes with the identified motif present in their promoters throughout the whole *E. dermatitidis* genome. Sheet 2: AME analysis which demonstrates the p-value and E-value for the enrichment of this motif within the highly regulated (>5 or <−5-fold), particle-responsive gene set.

Supplementary Table 7 STRING output of highly confident connections between genes that were upregulated among all particle sets, including genes making up specific nodes, confidence values for those nodes, and annotations for each gene.

Supplementary Table 8 The most highly connected, confident nodes identified in the particle-upregulated STRING analysis.

Supplementary Table 9 STRING output of highly confident connections between genes that were upregulated within each particle dataset, including genes making up specific nodes, confidence values for those nodes, and annotations for each gene.

Supplementary Table 10 STRING output of highly confident node connection annotations present in all WT particle irradiation-responsive datasets.

Supplementary Table 11 STRING output of highly confident node connection annotations present in only two WT particle irradiation-responsive datasets.

Supplementary Table 12 STRING output of highly confident node connection annotations present in only one WT particle irradiation-responsive dataset.
