# Supplementary material for: The Transcriptomic and Phenotypic Response of the Melanized Yeast Exophiala dermatitidis to Ionizing Particle Exposure
Source: Front Microbiol. 2021 Jan 12;11:609996. doi: 10.3389/fmicb.2020.609996 (PMC7835796; doi:10.3389/fmicb.2020.609996)
Supplement: Supplementary Figure 1 — (A) TPM correlation plots within deuteron, (B) proton, and (C)α-particle irradiated cultures, within biological replicates and between samples. [file Data_Sheet_1.docx]

**Table of Contents**

**Data Item Page**

Supplemental Table 1 2

Supplemental Table 2 3

Supplemental Figure 1 3

Supplemental Table 3 Separate File

Supplemental Table 4 Separate File

Supplemental Table 5 Separate File

Supplemental Table 6 Separate File

Supplemental Figure 2 5

Supplemental Table 7 Separate File

Supplemental Table 8 6

Supplemental Table 9 Separate File

Supplemental Table 10 7

Supplemental Table 11 7

Supplemental Table 12 7

|  | **Cell type** | **Dose (kGy)** | **Total expected colonies** | **Total observed colonies** | **Plating efficiency** |
| --- | --- | --- | --- | --- | --- |
| *α* | WT | 0.000 | 1350 | 911 | 0.675 |
|  |  | 0.125 | 2700 | 674 | 0.250 |
|  |  | 0.250 | 2700 | 604 | 0.224 |
|  |  | 0.500 | 2700 | 436 | 0.161 |
|  | PKS | 0.000 | 1350 | 492 | 0.364 |
|  |  | 0.125 | 2700 | 560 | 0.207 |
|  |  | 0.250 | 2700 | 881 | 0.326 |
|  |  | 0.500 | 2700 | 493 | 0.183 |
| *Proton* | WT | 0.000 | 1350 | 911 | 0.675 |
|  |  | 0.420 | 2700 | 625 | 0.231 |
|  |  | 0.840 | 2700 | 307 | 0.114 |
|  |  | 1.600 | 2700 | 249 | 0.092 |
|  | PKS | 0.000 | 1350 | 492 | 0.364 |
|  |  | 0.420 | 2700 | 586 | 0.217 |
|  |  | 0.840 | 2700 | 195 | 0.072 |
|  |  | 1.600 | 2700 | 229 | 0.085 |
| *Deuteron* | WT | 0.000 | 1350 | 773 | 0.573 |
|  |  | 0.250 | 2700 | 708 | 0.262 |
|  |  | 0.500 | 2700 | 222 | 0.082 |
|  |  | 1.000 | 2700 | 128 | 0.047 |
|  | PKS | 0.000 | 1350 | 324 | 0.240 |
|  |  | 0.250 | 2700 | 536 | 0.199 |
|  |  | 0.500 | 2700 | 299 | 0.111 |
|  |  | 1.000 | 2700 | 195 | 0.072 |

**Supplemental Table 1:** Colony forming unit and plating efficiency measurements for melanized (WT and non-melanized (PKS) strains upon irradiation by each particle type.

| **Cell type, radiation** | **α_min_** | **95% CIs (kGy^-1^)** | | **α_max_** | **95% CIs (kGy^-1^)** | | **δ** | **95% CIs (kGy^-1^)** | | **RBE** | **95% CIs** | |
| --- | --- | --- | --- | --- | --- | --- | --- | --- | --- | --- | --- | --- |
| WT, particle | **1.23** | 1.11 | 1.32 | **4.25** | 3.60 | 5.23 | **2.28** | 1.73 | 3.01 | **2.03** | 1.88 | 111.49 |
| PKS, particle | **1.08** | 1.01 | 1.16 | **50.00^*^** | 32.28 | 50.00 | **22.01** | 19.17 | 23.59 | **1.30** | 1.21 | 1.39 |

**Supplemental Table 2:** Best-fit models for each strain detailing parameters for particle irradiation survival curves.

**A**


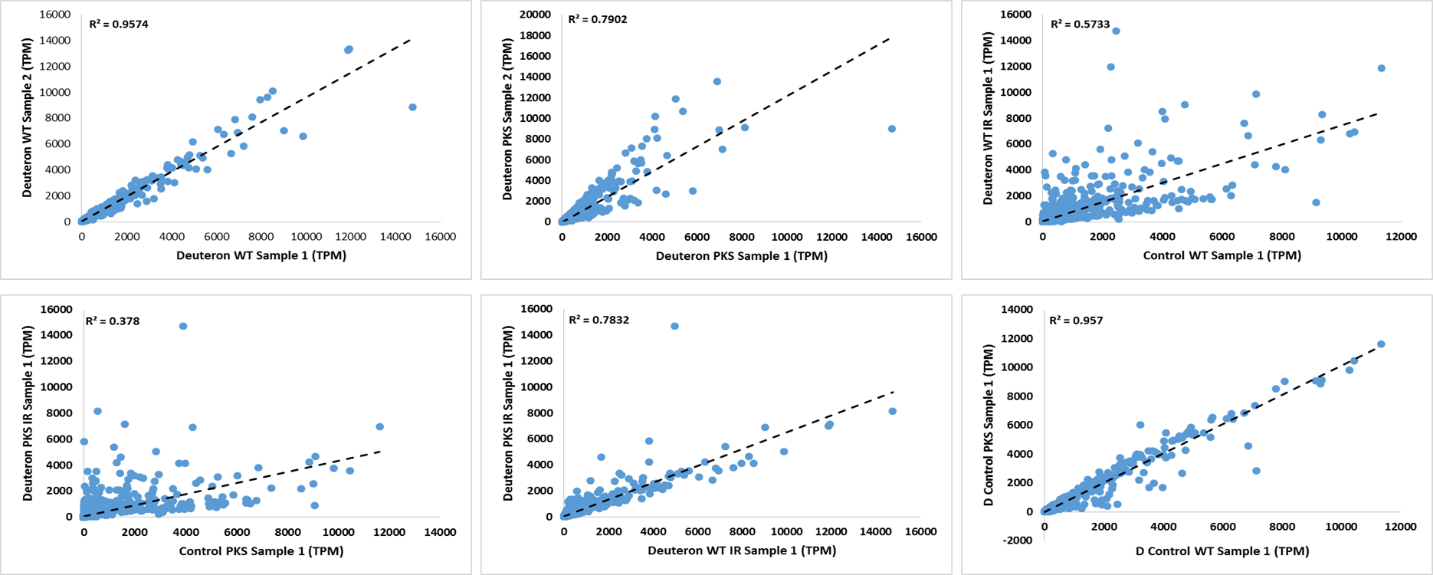


**B**


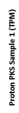

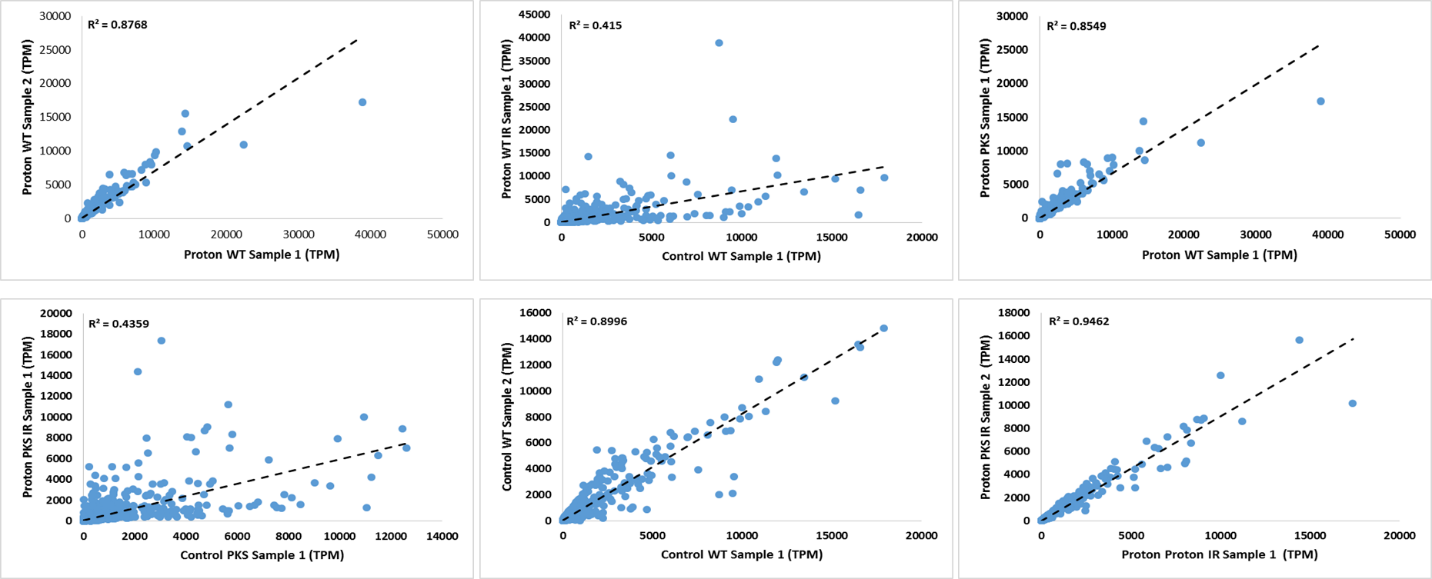


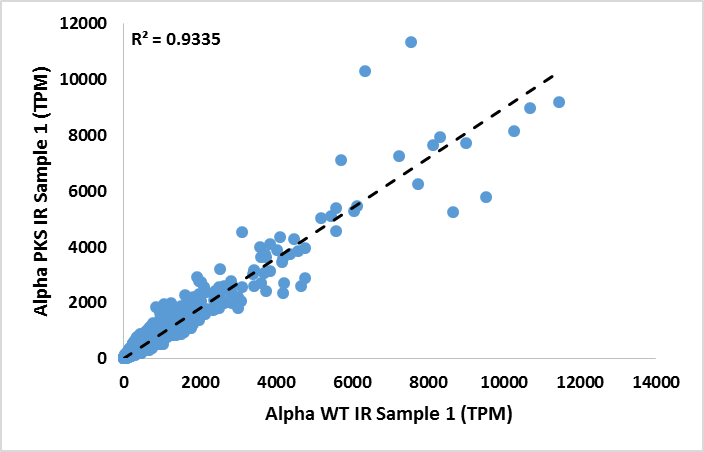

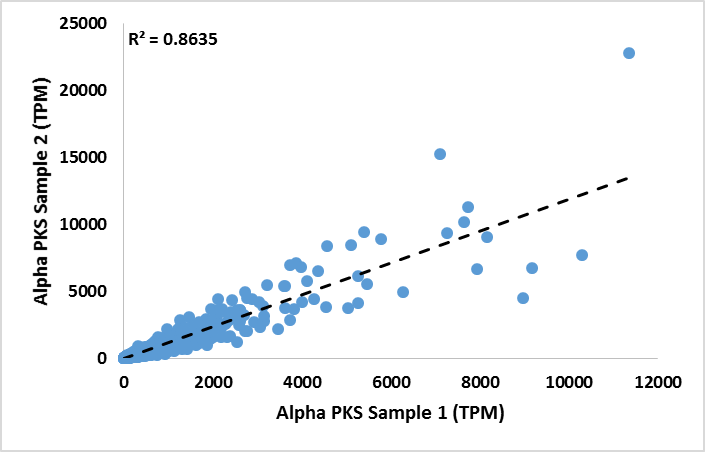


**C**


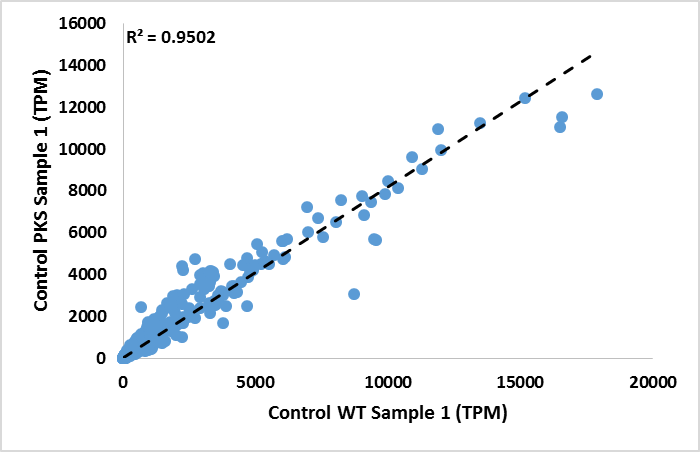

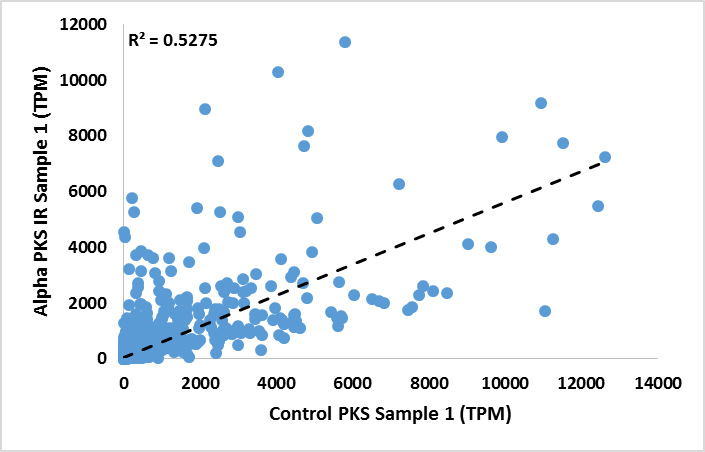


**Supplemental Figure 1. A.** TPM correlation plots within Deuteron, **B.** Proton, and **C.** α-particle irradiated cultures, within biological replicates and between samples.


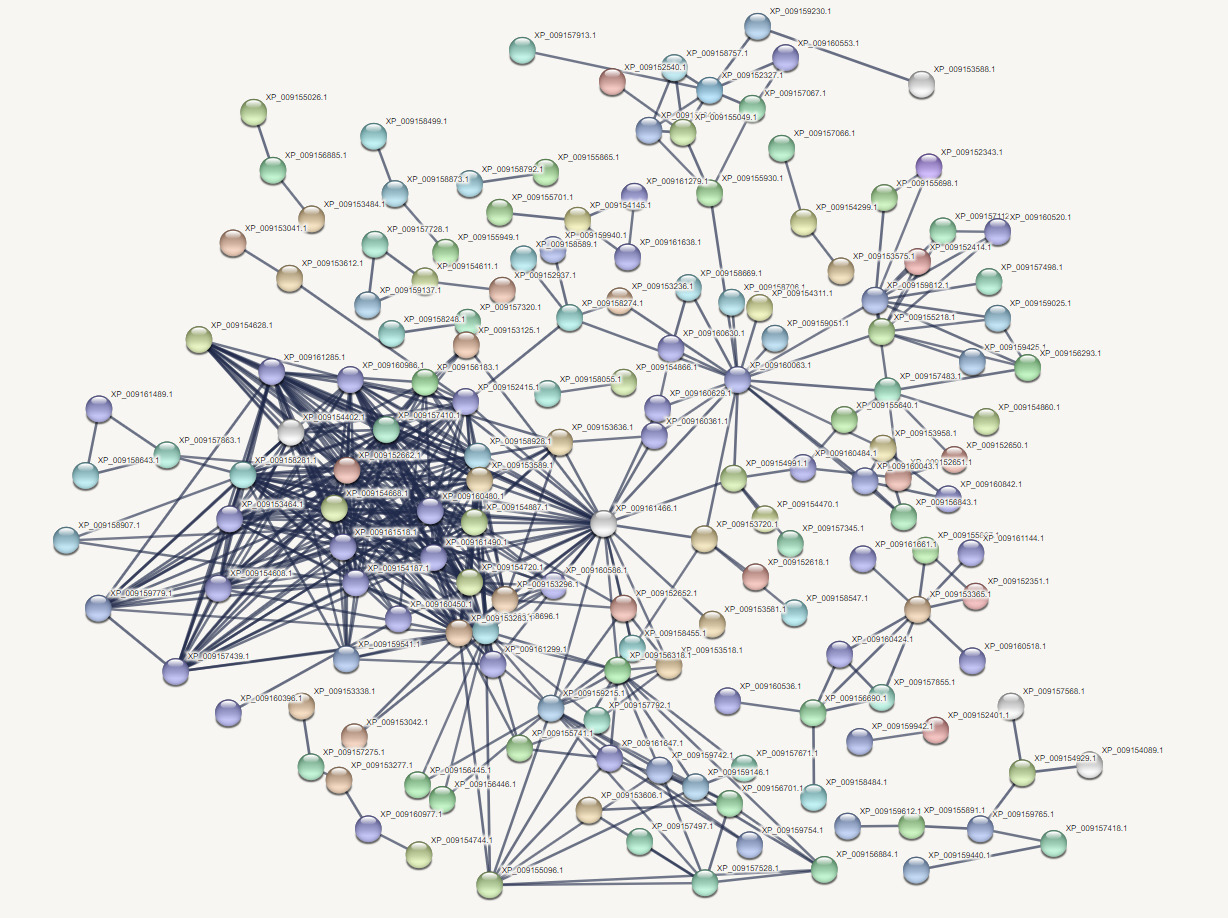


**Supplemental Figure 2.**

STRING network output showing highly confident connections between upregulated genes within the particle dataset.

| **Identified Nodes in Upregulated Gene Set** | | |
| --- | --- | --- |
| **Gene** | **Uniprot Annotation** | **Links** |
| HMPREF1120_08945 | Proliferating cell nuclear antigen | 34 |
| HMPREF1120_06247 | Replication protein A subunit | 29 |
| HMPREF1120_05844 | Minichromosome maintenance protein 2 | 29 |
| HMPREF1120_00416 | Minichromosome maintenance protein 7 | 27 |
| HMPREF1120_01029 | Replication factor A2 | 27 |
| HMPREF1120_08997 | DNA helicase | 26 |
| HMPREF1120_05011 | Minichromosome maintenance protein 6 | 25 |
| HMPREF1120_08969 | DNA polymerase epsilon subunit 2 | 23 |
| HMPREF1120_08768 | Minichromosome maintenance protein 3 | 23 |
| HMPREF1120_00177 | Cell division control protein 6 | 23 |
| HMPREF1120_07994 | DNA polymerase alpha subunit A | 20 |
| HMPREF1120_08481 | DNA primase large subunit | 20 |
| HMPREF1120_01328 | DNA polymerase alpha subunit B | 19 |
| HMPREF1120_06477 | DNA primase small subunit | 18 |
| HMPREF1120_01910 | Cell division control protein 45 | 18 |
| HMPREF1120_02379 | Minichromosome maintenance protein 5 | 18 |
| HMPREF1120_01204 | Origin recognition complex subunit 4 | 16 |
| HMPREF1120_01042 | DNA polymerase delta subunit 1 | 15 |
| HMPREF1120_07587 | Polyubiquitin | 15 |

**Supplemental Table 8.** The most highly connected, confident nodes identified in the particle-upregulated STRING analysis.


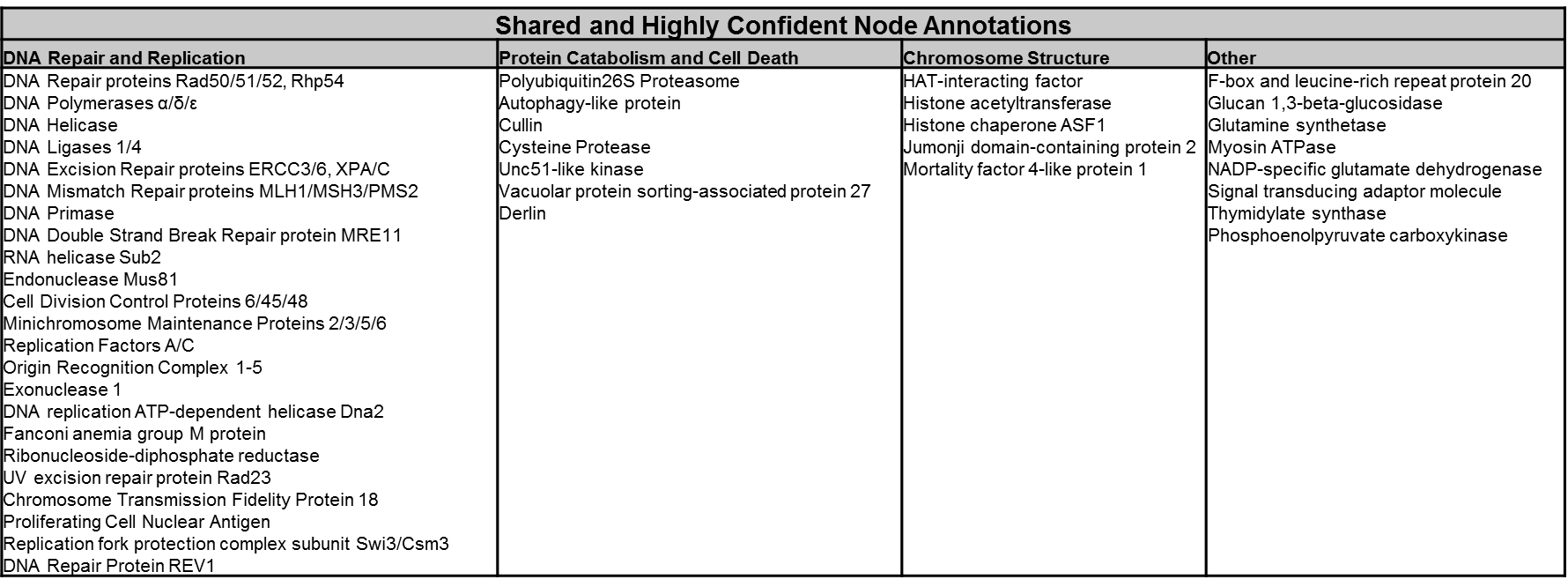
**Supplemental Table 10.** STRING output of highly confident node connection annotations present in all WT particle irradiation-responsive datasets.


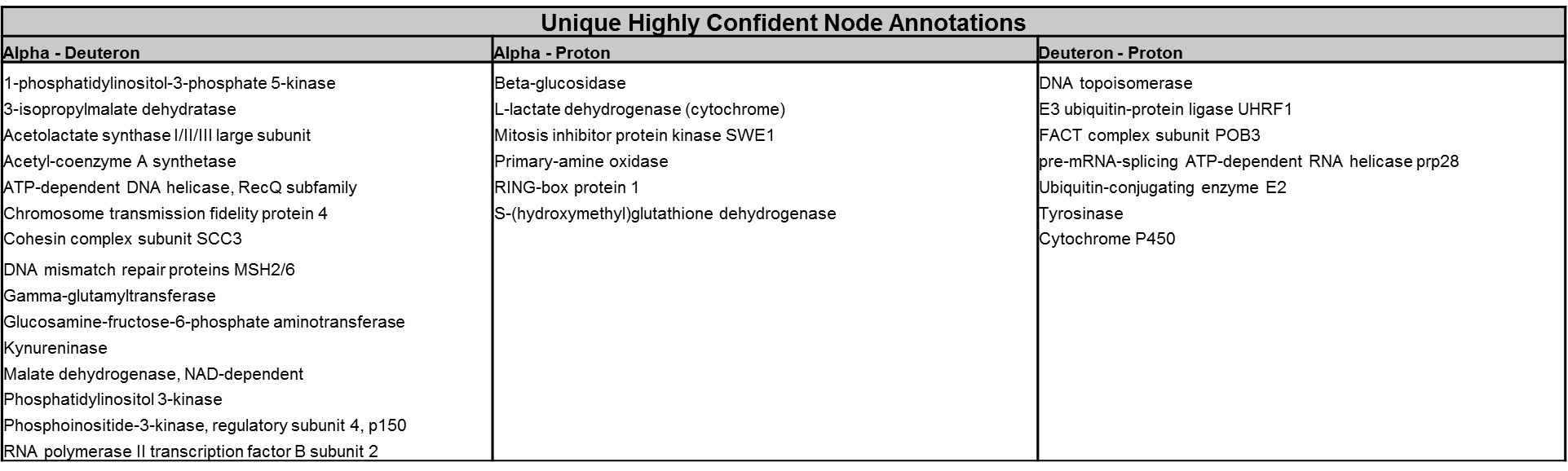
**Supplemental Table 11.** STRING output of highly confident node connection annotations present in only two WT particle irradiation-responsive datasets.
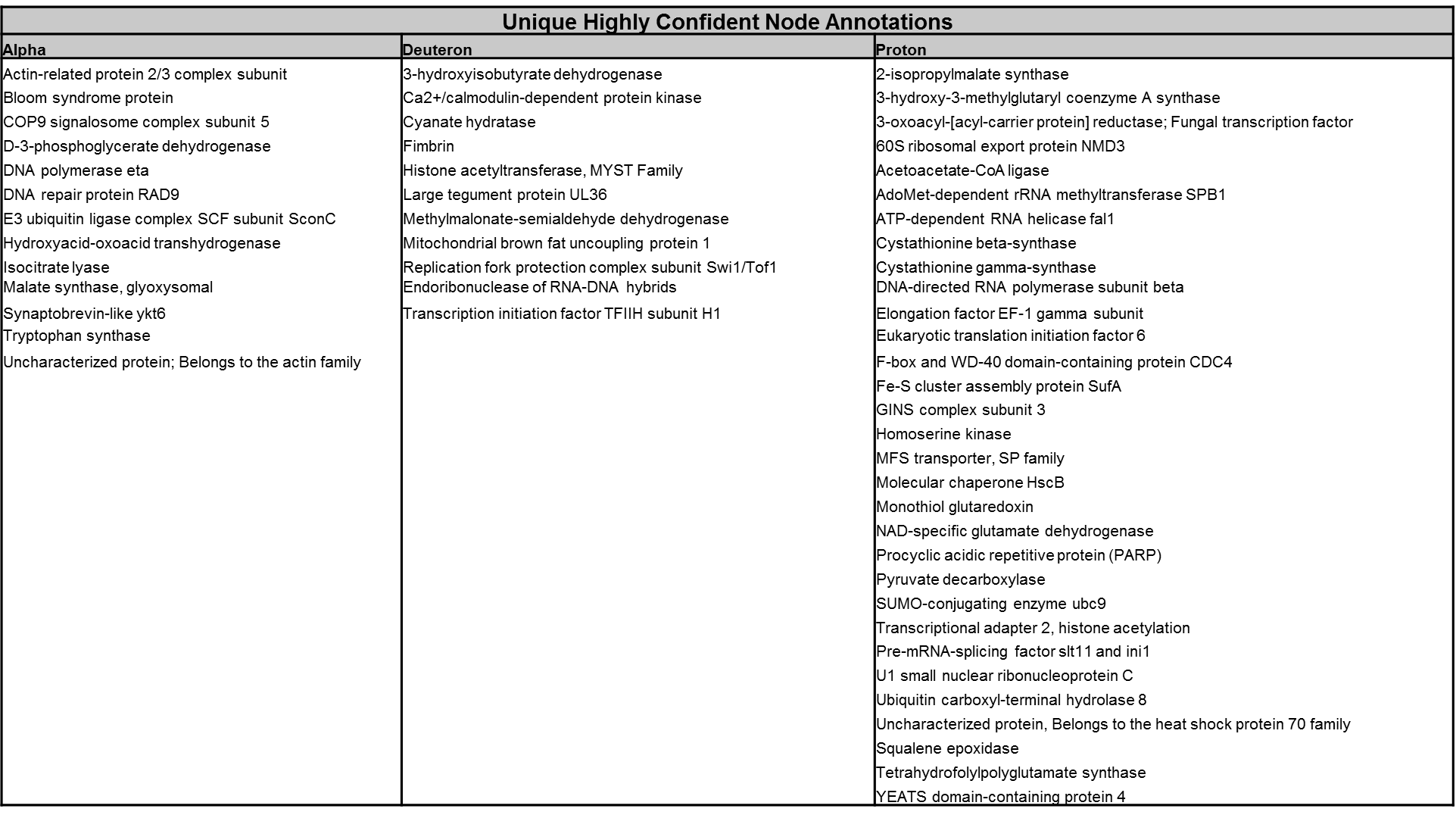
**Supplemental Table 12.** STRING output of highly confident node connection annotations present in only one WT particle irradiation-responsive dataset.
