# Supplementary material for: The Transcriptomic and Phenotypic Response of the Melanized Yeast Exophiala dermatitidis to Ionizing Particle Exposure
Source: Front Microbiol. 2021 Jan 12;11:609996. doi: 10.3389/fmicb.2020.609996 (PMC7835796; doi:10.3389/fmicb.2020.609996)
Supplement: Supplementary Table 5 — Raw output from MEME analysis of particle-regulated promoters (>5 or <−5-fold) that identified enriched motif associated with particle-responsive genes. [file Table_5.pdf]

****
MEME - Motif discovery tool
****
MEME version 5.1.1 (Release date: Wed Jan 29 15:00:42 2020 -0800)

For further information on how to interpret please access http://alternate.meme-suite.org/.
To get a copy of the MEME software please access http://meme-suite.org.

****

****
REFERENCE
****
If you use this program in your research, please cite:

Timothy L. Bailey and Charles Elkan,
"Fitting a mixture model by expectation maximization to discover
motifs in biopolymers", Proceedings of the Second International
Conference on Intelligent Systems for Molecular Biology, pp. 28-36,
AAAI Press, Menlo Park, California, 1994.
****

****
TRAINING SET
****
PRIMARY SEQUENCES= All_radiation_promoters_greater_than_five.txt
CONTROL SEQUENCES= --none--
ALPHABET= ACGT

Sequence name	Weight	Length	Sequence name	Weight	Length
-------------	------	------	-------------	------	------
HMPREF1120_00418	1.0000	1000	HMPREF1120_02408	1.0000	1000
HMPREF1120_01131	1.0000	1000	HMPREF1120_08232	1.0000	1000
HMPREF1120_01106	1.0000	1000	HMPREF1120_00342	1.0000	1000
HMPREF1120_05503	1.0000	1000	HMPREF1120_06548	1.0000	1000
HMPREF1120_06442	1.0000	1000	HMPREF1120_03392	1.0000	1000
HMPREF1120_00104	1.0000	1000	HMPREF1120_06522	1.0000	1000
HMPREF1120_02117	1.0000	1000	HMPREF1120_00243	1.0000	1000
HMPREF1120_07631	1.0000	1000	HMPREF1120_04377	1.0000	1000
HMPREF1120_07528	1.0000	1000	HMPREF1120_00627	1.0000	1000
HMPREF1120_06863	1.0000	1000	HMPREF1120_08881	1.0000	1000
HMPREF1120_04665	1.0000	1000	HMPREF1120_08459	1.0000	1000
HMPREF1120_03061	1.0000	1000	HMPREF1120_07509	1.0000	1000
HMPREF1120_06129	1.0000	1000	HMPREF1120_05730	1.0000	1000
HMPREF1120_01971	1.0000	1000	HMPREF1120_05552	1.0000	1000
HMPREF1120_02631	1.0000	1000	HMPREF1120_06634	1.0000	1000
HMPREF1120_03639	1.0000	1000	HMPREF1120_05086	1.0000	1000
HMPREF1120_03771	1.0000	1000	HMPREF1120_01653	1.0000	1000
HMPREF1120_02703	1.0000	1000	HMPREF1120_03835	1.0000	1000
HMPREF1120_02790	1.0000	1000	HMPREF1120_01095	1.0000	1000
HMPREF1120_03541	1.0000	1000	HMPREF1120_02172	1.0000	1000
HMPREF1120_00689	1.0000	1000	HMPREF1120_03048	1.0000	1000
HMPREF1120_03880	1.0000	1000	HMPREF1120_06931	1.0000	1000
HMPREF1120_08377	1.0000	1000	HMPREF1120_00312	1.0000	1000
HMPREF1120_04902	1.0000	1000	HMPREF1120_06823	1.0000	1000
HMPREF1120_06746	1.0000	1000	HMPREF1120_02714	1.0000	1000
HMPREF1120_08389	1.0000	1000	HMPREF1120_02139	1.0000	1000
HMPREF1120_05939	1.0000	1000	HMPREF1120_07217	1.0000	1000
HMPREF1120_07962	1.0000	1000	HMPREF1120_01351	1.0000	1000
HMPREF1120_01636	1.0000	1000	HMPREF1120_05323	1.0000	1000
HMPREF1120_04126	1.0000	1000	HMPREF1120_07074	1.0000	1000
HMPREF1120_05589	1.0000	1000	HMPREF1120_08630	1.0000	1000
HMPREF1120_04575	1.0000	1000	HMPREF1120_06595	1.0000	1000
HMPREF1120_04938	1.0000	1000	HMPREF1120_05123	1.0000	1000
HMPREF1120_06825	1.0000	1000	HMPREF1120_04438	1.0000	1000
HMPREF1120_03953	1.0000	1000	HMPREF1120_02531	1.0000	1000
HMPREF1120_05950	1.0000	1000	HMPREF1120_01967	1.0000	1000

HMPREF1120_05644	1.0000	1000	HMPREF1120_01546	1.0000	1000
HMPREF1120_00797	1.0000	1000	HMPREF1120_08663	1.0000	1000
HMPREF1120_05462	1.0000	1000	HMPREF1120_07533	1.0000	1000
HMPREF1120_03675	1.0000	1000	HMPREF1120_06581	1.0000	1000
HMPREF1120_02779	1.0000	1000	HMPREF1120_04883	1.0000	1000
HMPREF1120_04910	1.0000	1000	HMPREF1120_07397	1.0000	1000
HMPREF1120_02089	1.0000	1000	HMPREF1120_07479	1.0000	1000
HMPREF1120_02715	1.0000	1000	HMPREF1120_04920	1.0000	1000
HMPREF1120_08523	1.0000	1000	HMPREF1120_05339	1.0000	1000
HMPREF1120_06790	1.0000	1000	HMPREF1120_06758	1.0000	1000
HMPREF1120_07664	1.0000	1000	HMPREF1120_03399	1.0000	1000
HMPREF1120_02769	1.0000	1000	HMPREF1120_03104	1.0000	1000
HMPREF1120_05710	1.0000	1000	HMPREF1120_02138	1.0000	1000
HMPREF1120_05753	1.0000	1000	HMPREF1120_07854	1.0000	1000
HMPREF1120_04684	1.0000	1000	HMPREF1120_00585	1.0000	1000
HMPREF1120_06490	1.0000	1000	HMPREF1120_02566	1.0000	1000
HMPREF1120_07951	1.0000	1000	HMPREF1120_05725	1.0000	1000
HMPREF1120_07128	1.0000	1000	HMPREF1120_02118	1.0000	1000
HMPREF1120_05801	1.0000	1000	HMPREF1120_06188	1.0000	1000
HMPREF1120_03768	1.0000	1000	HMPREF1120_00286	1.0000	1000
HMPREF1120_02424	1.0000	1000	HMPREF1120_00652	1.0000	1000
HMPREF1120_08713	1.0000	1000	HMPREF1120_07632	1.0000	1000
HMPREF1120_00149	1.0000	1000	HMPREF1120_02287	1.0000	1000
HMPREF1120_04393	1.0000	1000	HMPREF1120_06862	1.0000	1000
HMPREF1120_06895	1.0000	1000	HMPREF1120_01920	1.0000	1000
HMPREF1120_06329	1.0000	1000	HMPREF1120_05252	1.0000	1000
HMPREF1120_02898	1.0000	1000	HMPREF1120_00045	1.0000	1000
HMPREF1120_05635	1.0000	1000	HMPREF1120_03442	1.0000	1000
HMPREF1120_01547	1.0000	1000	HMPREF1120_07039	1.0000	1000
HMPREF1120_02634	1.0000	1000	HMPREF1120_03876	1.0000	1000
HMPREF1120_02275	1.0000	1000	HMPREF1120_03066	1.0000	1000
HMPREF1120_02373	1.0000	1000	HMPREF1120_06864	1.0000	1000
HMPREF1120_02274	1.0000	1000	HMPREF1120_08525	1.0000	1000
HMPREF1120_07050	1.0000	1000	HMPREF1120_05273	1.0000	1000
HMPREF1120_07048	1.0000	1000	HMPREF1120_04516	1.0000	1000
HMPREF1120_07996	1.0000	1000	HMPREF1120_03503	1.0000	1000
HMPREF1120_02653	1.0000	1000	HMPREF1120_01129	1.0000	1000
HMPREF1120_01052	1.0000	1000	HMPREF1120_01536	1.0000	1000
HMPREF1120_06676	1.0000	1000	HMPREF1120_08308	1.0000	1000
HMPREF1120_04375	1.0000	1000	HMPREF1120_09262	1.0000	1000
HMPREF1120_00244	1.0000	1000	HMPREF1120_06537	1.0000	1000
HMPREF1120_00235	1.0000	1000	HMPREF1120_01435	1.0000	1000
HMPREF1120_01637	1.0000	1000	HMPREF1120_07508	1.0000	1000
HMPREF1120_04934	1.0000	1000	HMPREF1120_02072	1.0000	1000
HMPREF1120_02791	1.0000	1000	HMPREF1120_04834	1.0000	1000
HMPREF1120_01817	1.0000	1000	HMPREF1120_03202	1.0000	1000
HMPREF1120_07674	1.0000	1000	HMPREF1120_07633	1.0000	1000
HMPREF1120_01995	1.0000	1000	HMPREF1120_00159	1.0000	1000
HMPREF1120_08773	1.0000	1000	HMPREF1120_04488	1.0000	1000
HMPREF1120_03846	1.0000	1000	HMPREF1120_00041	1.0000	1000
HMPREF1120_05330	1.0000	1000	HMPREF1120_07767	1.0000	1000
HMPREF1120_01851	1.0000	1000	HMPREF1120_06902	1.0000	1000
HMPREF1120_09146	1.0000	1000	HMPREF1120_03095	1.0000	1000
HMPREF1120_07560	1.0000	1000	HMPREF1120_00546	1.0000	1000
HMPREF1120_04401	1.0000	1000	HMPREF1120_03941	1.0000	1000
HMPREF1120_05534	1.0000	1000	HMPREF1120_01968	1.0000	1000
HMPREF1120_05435	1.0000	1000	HMPREF1120_02359	1.0000	1000
HMPREF1120_04788	1.0000	1000	HMPREF1120_05477	1.0000	1000
HMPREF1120_06974	1.0000	1000	HMPREF1120_04831	1.0000	1000
HMPREF1120_02501	1.0000	1000	HMPREF1120_04925	1.0000	1000
HMPREF1120_02021	1.0000	1000	HMPREF1120_06363	1.0000	1000
HMPREF1120_07673	1.0000	1000	HMPREF1120_07049	1.0000	1000
HMPREF1120_07792	1.0000	1000	HMPREF1120_03151	1.0000	1000
HMPREF1120_08650	1.0000	1000	HMPREF1120_06770	1.0000	1000
HMPREF1120_07390	1.0000	1000	HMPREF1120_05935	1.0000	1000
HMPREF1120_01293	1.0000	1000	HMPREF1120_07658	1.0000	1000
HMPREF1120_06441	1.0000	1000	HMPREF1120_00898	1.0000	1000
HMPREF1120_08578	1.0000	1000	HMPREF1120_06580	1.0000	1000
HMPREF1120_07908	1.0000	1000	HMPREF1120_07157	1.0000	1000

HMPREF1120_06748	1.0000	1000	HMPREF1120_01579	1.0000	1000
HMPREF1120_07981	1.0000	1000	HMPREF1120_04574	1.0000	1000
HMPREF1120_03688	1.0000	1000	HMPREF1120_03067	1.0000	1000
HMPREF1120_08344	1.0000	1000	HMPREF1120_04843	1.0000	1000
HMPREF1120_06583	1.0000	1000	HMPREF1120_01375	1.0000	1000
HMPREF1120_08154	1.0000	1000	HMPREF1120_02698	1.0000	1000
HMPREF1120_08138	1.0000	1000	HMPREF1120_08448	1.0000	1000
HMPREF1120_08742	1.0000	1000	HMPREF1120_00007	1.0000	1000
HMPREF1120_02578	1.0000	1000	HMPREF1120_02127	1.0000	1000
HMPREF1120_06618	1.0000	1000	HMPREF1120_02904	1.0000	1000
HMPREF1120_00716	1.0000	1000	HMPREF1120_06341	1.0000	1000
HMPREF1120_04563	1.0000	1000	HMPREF1120_06646	1.0000	1000
HMPREF1120_02776	1.0000	1000	HMPREF1120_06679	1.0000	1000
HMPREF1120_05155	1.0000	1000	HMPREF1120_01512	1.0000	858
HMPREF1120_01172	1.0000	1000	HMPREF1120_09135	1.0000	1000
HMPREF1120_08781	1.0000	1000	HMPREF1120_01519	1.0000	1000
HMPREF1120_00622	1.0000	1000	HMPREF1120_03297	1.0000	1000
HMPREF1120_08211	1.0000	1000	HMPREF1120_05905	1.0000	1000
HMPREF1120_03649	1.0000	1000	HMPREF1120_00621	1.0000	1000
HMPREF1120_01082	1.0000	1000	HMPREF1120_08679	1.0000	1000
HMPREF1120_06393	1.0000	1000	HMPREF1120_03733	1.0000	1000
HMPREF1120_00978	1.0000	1000	HMPREF1120_00006	1.0000	1000
HMPREF1120_06138	1.0000	1000	HMPREF1120_04842	1.0000	1000
HMPREF1120_02509	1.0000	1000	HMPREF1120_08480	1.0000	1000
HMPREF1120_00717	1.0000	1000	HMPREF1120_07062	1.0000	1000
HMPREF1120_08684	1.0000	1000	HMPREF1120_05439	1.0000	1000
HMPREF1120_07638	1.0000	1000	HMPREF1120_00405	1.0000	1000
HMPREF1120_08447	1.0000	1000	HMPREF1120_01764	1.0000	1000
HMPREF1120_04550	1.0000	1000	HMPREF1120_02510	1.0000	1000
HMPREF1120_04120	1.0000	1000	HMPREF1120_03732	1.0000	1000
HMPREF1120_02286	1.0000	1000	HMPREF1120_03724	1.0000	1000
HMPREF1120_06645	1.0000	1000	HMPREF1120_04812	1.0000	1000
HMPREF1120_05987	1.0000	1000	HMPREF1120_07793	1.0000	1000
HMPREF1120_00375	1.0000	1000	HMPREF1120_01149	1.0000	1000
HMPREF1120_02526	1.0000	1000	HMPREF1120_02305	1.0000	1000
HMPREF1120_06671	1.0000	1000	HMPREF1120_05694	1.0000	1000
HMPREF1120_09019	1.0000	1000	HMPREF1120_06616	1.0000	1000
HMPREF1120_05142	1.0000	1000	HMPREF1120_07623	1.0000	1000
HMPREF1120_09134	1.0000	1000	HMPREF1120_05354	1.0000	1000
HMPREF1120_01508	1.0000	1000	HMPREF1120_00838	1.0000	1000
HMPREF1120_07321	1.0000	1000	HMPREF1120_02454	1.0000	1000
HMPREF1120_06644	1.0000	1000	HMPREF1120_08477	1.0000	1000
HMPREF1120_05234	1.0000	1000	HMPREF1120_03386	1.0000	1000
HMPREF1120_05842	1.0000	1000	HMPREF1120_00264	1.0000	1000
HMPREF1120_06777	1.0000	1000	HMPREF1120_04224	1.0000	1000
HMPREF1120_01423	1.0000	1000	HMPREF1120_06279	1.0000	1000
HMPREF1120_05665	1.0000	1000	HMPREF1120_08115	1.0000	1000
HMPREF1120_05585	1.0000	1000	HMPREF1120_02014	1.0000	1000
HMPREF1120_03609	1.0000	1000	HMPREF1120_00924	1.0000	1000
HMPREF1120_01403	1.0000	1000	HMPREF1120_01833	1.0000	1000
HMPREF1120_07608	1.0000	1000	HMPREF1120_01522	1.0000	1000
HMPREF1120_06047	1.0000	1000	HMPREF1120_01923	1.0000	1000
HMPREF1120_05100	1.0000	1000	HMPREF1120_09238	1.0000	1000
HMPREF1120_00374	1.0000	1000	HMPREF1120_06528	1.0000	1000
HMPREF1120_07676	1.0000	1000	HMPREF1120_00038	1.0000	1000
HMPREF1120_01099	1.0000	1000	HMPREF1120_05642	1.0000	1000
HMPREF1120_06290	1.0000	1000	HMPREF1120_09136	1.0000	1000
HMPREF1120_03296	1.0000	1000	HMPREF1120_03603	1.0000	1000
HMPREF1120_07937	1.0000	1000	HMPREF1120_04140	1.0000	1000
HMPREF1120_05088	1.0000	1000	HMPREF1120_01710	1.0000	1000
HMPREF1120_03451	1.0000	1000	HMPREF1120_01909	1.0000	1000
HMPREF1120_03636	1.0000	1000	HMPREF1120_01980	1.0000	1000
HMPREF1120_03973	1.0000	1000	HMPREF1120_01020	1.0000	1000
HMPREF1120_07372	1.0000	1000	HMPREF1120_00101	1.0000	1000
HMPREF1120_07003	1.0000	1000	HMPREF1120_08674	1.0000	1000
HMPREF1120_04734	1.0000	1000	HMPREF1120_00197	1.0000	1000
HMPREF1120_01451	1.0000	1000	HMPREF1120_01182	1.0000	1000
HMPREF1120_03772	1.0000	1000	HMPREF1120_04068	1.0000	1000
HMPREF1120_01282	1.0000	1000	HMPREF1120_05091	1.0000	1000

```
HMPREF1120_05843         1.0000   1000  HMPREF1120_05820         1.0000   1000
HMPREF1120_03646         1.0000   1000  HMPREF1120_04805         1.0000   1000
HMPREF1120_01777         1.0000   1000  HMPREF1120_06780         1.0000   1000
HMPREF1120_07526         1.0000   1000  HMPREF1120_00198         1.0000   1000
HMPREF1120_05027         1.0000   1000  HMPREF1120_04430         1.0000   1000
HMPREF1120_07017         1.0000   1000  HMPREF1120_09162         1.0000   1000
HMPREF1120_08770         1.0000   1000  HMPREF1120_06046         1.0000   1000
HMPREF1120_05715         1.0000   1000  HMPREF1120_01343         1.0000   1000
HMPREF1120_01315         1.0000   1000  HMPREF1120_03196         1.0000   1000
HMPREF1120_03454         1.0000   1000  HMPREF1120_00902         1.0000   1000
HMPREF1120_02198         1.0000   1000  HMPREF1120_05841         1.0000   1000
HMPREF1120_07391         1.0000   1000  HMPREF1120_00954         1.0000   1000
HMPREF1120_08769         1.0000   1000  HMPREF1120_07879         1.0000   1000
HMPREF1120_05009         1.0000   1000  HMPREF1120_03199         1.0000   1000
HMPREF1120_00196         1.0000   1000  HMPREF1120_06146         1.0000   1000
HMPREF1120_02306         1.0000   1000  HMPREF1120_01981         1.0000   1000
HMPREF1120_04784         1.0000   1000  HMPREF1120_05366         1.0000   1000
HMPREF1120_06238         1.0000   1000  HMPREF1120_02122         1.0000   1000
HMPREF1120_06617         1.0000   1000  HMPREF1120_06465         1.0000   1000
HMPREF1120_07460         1.0000   1000  HMPREF1120_03504         1.0000   1000
HMPREF1120_06251         1.0000   1000  HMPREF1120_06104         1.0000   1000
HMPREF1120_05260         1.0000   1000  HMPREF1120_02938         1.0000   1000
HMPREF1120_06484         1.0000   1000  HMPREF1120_06164         1.0000   1000
HMPREF1120_06689         1.0000   1000  HMPREF1120_01406         1.0000   1000
HMPREF1120_00109         1.0000   1000  HMPREF1120_05453         1.0000   1000
HMPREF1120_04014         1.0000   1000  HMPREF1120_06399         1.0000   1000
HMPREF1120_00404         1.0000   1000  HMPREF1120_06148         1.0000   1000
HMPREF1120_02792         1.0000   1000  HMPREF1120_08633         1.0000   1000
HMPREF1120_02853         1.0000   1000  HMPREF1120_01156         1.0000   1000
HMPREF1120_01982         1.0000   1000  HMPREF1120_07880         1.0000   1000
HMPREF1120_06804         1.0000   1000  HMPREF1120_02225         1.0000   1000
HMPREF1120_01029         1.0000   1000  HMPREF1120_03745         1.0000   1000
HMPREF1120_03411         1.0000   1000  HMPREF1120_05790         1.0000   1000
HMPREF1120_07971         1.0000   1000  HMPREF1120_02802         1.0000   1000
HMPREF1120_01015         1.0000   1000  HMPREF1120_07241         1.0000   1000
HMPREF1120_01137         1.0000   1000  HMPREF1120_01523         1.0000   1000
HMPREF1120_03195         1.0000   1000  HMPREF1120_08439         1.0000   1000
HMPREF1120_04980         1.0000   1000  HMPREF1120_08783         1.0000   1000
HMPREF1120_07216         1.0000   1000  HMPREF1120_03803         1.0000   1000
HMPREF1120_04197         1.0000   1000  HMPREF1120_07677         1.0000   1000
HMPREF1120_06028         1.0000   1000  HMPREF1120_06922         1.0000   1000
HMPREF1120_06027         1.0000   1000  HMPREF1120_04736         1.0000   1000
HMPREF1120_08058         1.0000   1000  HMPREF1120_03622         1.0000   1000
HMPREF1120_00523         1.0000   1000  HMPREF1120_06308         1.0000   1000
HMPREF1120_04947         1.0000   1000  HMPREF1120_01818         1.0000   1000
HMPREF1120_01896         1.0000   1000  HMPREF1120_03417         1.0000   1000
HMPREF1120_07182         1.0000   1000  HMPREF1120_08026         1.0000   1000
HMPREF1120_04558         1.0000   1000  HMPREF1120_04424         1.0000   1000
HMPREF1120_00714         1.0000   1000  HMPREF1120_06026         1.0000   1000
HMPREF1120_05486         1.0000   1000  HMPREF1120_02756         1.0000   1000
HMPREF1120_02623         1.0000   1000  HMPREF1120_08078         1.0000   1000
HMPREF1120_04804         1.0000   1000  HMPREF1120_06752         1.0000   1000
HMPREF1120_04359         1.0000   1000  HMPREF1120_05433         1.0000   1000
HMPREF1120_02391         1.0000   1000  HMPREF1120_08476         1.0000   1000
HMPREF1120_05622         1.0000   1000  HMPREF1120_01983         1.0000   1000
HMPREF1120_05846         1.0000   1000  HMPREF1120_05848         1.0000   1000
HMPREF1120_03154         1.0000   1000  HMPREF1120_00901         1.0000   1000
HMPREF1120_03453         1.0000   1000  HMPREF1120_06719         1.0000   1000
HMPREF1120_01453         1.0000   1000  HMPREF1120_01601         1.0000   1000
HMPREF1120_07322         1.0000   1000
********************************************************************************

********************************************************************************
COMMAND LINE SUMMARY
********************************************************************************
This information can also be useful in the event you wish to report a
problem with the MEME software.

command: meme All_radiation_promoters_greater_than_five.txt -dna -oc . -nostatus -time 17929 -mod zoops -
```

```
nmotifs 10 -minw 6 -maxw 50 -objfun classic -revcomp -markov_order 0 -psp priors.psp

model:  mod=         zoops    nmotifs=        10    evt=           inf
objective function:           em=       E-value of product of p-values
                              starts=   E-value of product of p-values
strands: + -
width:  minw=            6    maxw=           50
nsites: minsites=        2    maxsites=      467    wnsites=       0.8
theta:  spmap=         uni    spfuzz=        0.5
em:     prior=   dirichlet    b=            0.01    maxiter=        50
        distance=    1e-05
trim:   wg=             11    ws=              1    endgaps=       yes
data:   n=          466858    N=             467
sample: seed=            0    hsfrac=          0
        searchsize= 100000    norand=         no    csites=       1000
Letter frequencies in dataset:
A 0.243 C 0.257 G 0.257 T 0.243
Background letter frequencies (from file dataset with add-one prior applied):
A 0.243 C 0.257 G 0.257 T 0.243
Background model order: 0
********************************************************************************


********************************************************************************
MOTIF STTCAAGTT MEME-1  width =   9  sites = 165  llr = 1677  E-value = 8.3e-005
********************************************************************************
--------------------------------------------------------------------------------
        Motif STTCAAGTT MEME-1 Description
--------------------------------------------------------------------------------
Simplified        A  1:::aa:::
pos.-specific     C  6::8::::1
probability       G  3::2::a:1
matrix            T  :aa::::a8

         bits    2.0  ** **
                 1.8  ** ****
                 1.6  ** ****
                 1.4  ** ****
Relative         1.2  *******
Entropy          1.0  ********
(14.7 bits)      0.8  ********
                 0.6 *********
                 0.4 *********
                 0.2 *********
                 0.0 ---------

Multilevel           CTTCAAGTT
consensus            G  G
sequence

--------------------------------------------------------------------------------

--------------------------------------------------------------------------------
        Motif STTCAAGTT MEME-1 sites sorted by position p-value
--------------------------------------------------------------------------------
Sequence name            Strand  Start   P-value             Site
-------------            ------  ----- ---------           ---------
HMPREF1120_01601             +    614  3.49e-06 AGCAGCACCA CTTCAAGTT GGCCTGCCTG
HMPREF1120_05622             +    124  3.49e-06 ACACCCGCCG CTTCAAGTT GTTCGTTGCC
HMPREF1120_02391             -    554  3.49e-06 TGACGCGGGA CTTCAAGTT GGAGAAGCGG
HMPREF1120_02756             +    523  3.49e-06 CAGCCGTCAA CTTCAAGTT CTAATAAACG
HMPREF1120_03417             +    850  3.49e-06 AGTTCGAGTT CTTCAAGTT CAACCCCGGA
HMPREF1120_03803             +    899  3.49e-06 AAATGAGGAT CTTCAAGTT CGGGACTTGA
HMPREF1120_08783             -     40  3.49e-06 GCCGGGCGCA CTTCAAGTT GGATTAATCG
HMPREF1120_07241             +    663  3.49e-06 AACACGAACA CTTCAAGTT GGAGACCAGG
HMPREF1120_05790             +    208  3.49e-06 ATTCAAACCC CTTCAAGTT TCGTGTCTAC
HMPREF1120_02792             +    347  3.49e-06 CCAGTTGACA CTTCAAGTT GGCGGGGGAT
HMPREF1120_06484             +    360  3.49e-06 GTGAGGCAGG CTTCAAGTT CGCGATATCT
HMPREF1120_02938             +    480  3.49e-06 GGTACCGGTA CTTCAAGTT GCACCCGCGA
```

```
HMPREF1120_06465         +    462  3.49e-06 AAACAATCGC CTTCAAGTT TAGGTCCCTC
HMPREF1120_07879         -    754  3.49e-06 CGGGCGCGAG CTTCAAGTT TGCTTTATCC
HMPREF1120_00198         -    370  3.49e-06 CCTCTCCAGA CTTCAAGTT TTGCCAGCAC
HMPREF1120_01182         -    709  3.49e-06 AACGCATTCA CTTCAAGTT CAACTTGAGT
HMPREF1120_04734         +    975  3.49e-06 CTCCCAACAT CTTCAAGTT CTCTCAACTT
HMPREF1120_03451         -    193  3.49e-06 CCGAAGCGAA CTTCAAGTT TGCGCCGGGC
HMPREF1120_01099         -    447  3.49e-06 GAAGCTGGGA CTTCAAGTT GAGGGTGTCA
HMPREF1120_00038         +     12  3.49e-06 TACGTAAGGG CTTCAAGTT GCCATACATG
HMPREF1120_07676         +    984  3.49e-06 TGAAGTTGTG CTTCAAGTT CATTATCT
HMPREF1120_01403         -    562  3.49e-06 TCCATTCTCT CTTCAAGTT CGGCATCTTC
HMPREF1120_04224         -    863  3.49e-06 CGAGAGAGAG CTTCAAGTT CCTTATACCC
HMPREF1120_06777         +    455  3.49e-06 GAGTGCTGAC CTTCAAGTT GATGAACGGC
HMPREF1120_00264         -    754  3.49e-06 CTCGGAGGAG CTTCAAGTT CGATTCCTGG
HMPREF1120_06644         +    789  3.49e-06 TGGCTTGCCT CTTCAAGTT GTCAATATCG
HMPREF1120_09134         +    398  3.49e-06 TTGATTTGTT CTTCAAGTT TTACCATTTC
HMPREF1120_07623         +    503  3.49e-06 TTATCTCTGA CTTCAAGTT CGCCAGCAAC
HMPREF1120_09019         +    781  3.49e-06 CCTTTGCACG CTTCAAGTT CCCAGCGAAG
HMPREF1120_02305         +    416  3.49e-06 CCCGCTCAAG CTTCAAGTT CGCACTACAG
HMPREF1120_06645         -    824  3.49e-06 TGGCTTGCCT CTTCAAGTT GTCAATATCG
HMPREF1120_02286         -    802  3.49e-06 GATCACTGCA CTTCAAGTT CGCTTGGGAG
HMPREF1120_03649         -    159  3.49e-06 AATGCTGTCC CTTCAAGTT TCAATCGTGT
HMPREF1120_02776         +    731  3.49e-06 ATATTCCAAC CTTCAAGTT TCCCAAAGTT
HMPREF1120_08138         -    627  3.49e-06 CTACCAGCGC CTTCAAGTT TGCTAGACTG
HMPREF1120_08154         +    513  3.49e-06 CTTCTGGCCC CTTCAAGTT GGACGCCTAG
HMPREF1120_01375         -    340  3.49e-06 CCGCGCCCGG CTTCAAGTT GCCCTGACAA
HMPREF1120_01579         -    752  3.49e-06 CTCTCCCTTG CTTCAAGTT CTCCCTCATC
HMPREF1120_08650         +    730  3.49e-06 CAGGCTTCAA CTTCAAGTT TGCGACGACA
HMPREF1120_02021         -    823  3.49e-06 TGCTGCAGAC CTTCAAGTT CAATTTCGCT
HMPREF1120_02359         -    255  3.49e-06 ACGTGTACCA CTTCAAGTT GAACACACGC
HMPREF1120_01968         -    980  3.49e-06 TGGTCAATGC CTTCAAGTT GTGGGGAACG
HMPREF1120_07996         +    509  3.49e-06 CATTGGCGGA CTTCAAGTT CGAGCCATGC
HMPREF1120_02634         +     19  3.49e-06 AGTCCGCTCT CTTCAAGTT TCTGCCATTT
HMPREF1120_00652         -    530  3.49e-06 CGATCGTCCG CTTCAAGTT GCTGCTGCTG
HMPREF1120_06490         -    663  3.49e-06 GAGGAGGCTG CTTCAAGTT GTTCGCTGAC
HMPREF1120_05710         -    188  3.49e-06 AAATTTCAAT CTTCAAGTT GGTGTACAAA
HMPREF1120_07664         +    939  3.49e-06 AAAAAGCTCT CTTCAAGTT TCGATCGGCG
HMPREF1120_01967         +    952  3.49e-06 TGGTCAATGC CTTCAAGTT GTGGGGAACG
HMPREF1120_05950         -    954  3.49e-06 ATTGCCCAGT CTTCAAGTT CCATTACTAG
HMPREF1120_07962         -    697  3.49e-06 TGGAAGCACC CTTCAAGTT CCTCTGCAGA
HMPREF1120_02631         -    259  3.49e-06 AGTCCGCTCT CTTCAAGTT TCTGCCATTT
HMPREF1120_06129         -    937  3.49e-06 CAAAGGCTGG CTTCAAGTT GTACCACTGG
HMPREF1120_07528         -    486  3.49e-06 ACTCTACAAT CTTCAAGTT TGCTCAGATC
HMPREF1120_00418         -    806  3.49e-06 AGATTCATGT CTTCAAGTT GTCAGTGTTC
HMPREF1120_08476         -    949  6.99e-06 GTGTGATGGC GTTCAAGTT TGTCCTGCAC
HMPREF1120_05486         +    399  6.99e-06 AAAATCTCTG GTTCAAGTT CGCGGAGACA
HMPREF1120_07182         -    553  6.99e-06 CCAGACTAAG GTTCAAGTT GTAGATATGG
HMPREF1120_01818         -    229  6.99e-06 GATGCTCGTC GTTCAAGTT GGTGATGTGG
HMPREF1120_00523         +    304  6.99e-06 CACGCGCAGG GTTCAAGTT TGTCTGGGGA
HMPREF1120_06804         +    798  6.99e-06 GCGCATCGCT GTTCAAGTT GTCTTCGGCG
HMPREF1120_01982         -    590  6.99e-06 ACGACCTCAT GTTCAAGTT CAACGAATCC
HMPREF1120_05841         -    486  6.99e-06 GTGTTTGTAG GTTCAAGTT GTCGATTCGT
HMPREF1120_00902         +    315  6.99e-06 GAGTGAGTGA GTTCAAGTT GCTAGAGCCT
HMPREF1120_01020         +    102  6.99e-06 TGCCTGCGCT GTTCAAGTT GCTCGATGTT
HMPREF1120_03603         -    794  6.99e-06 TGGAAGAGCA GTTCAAGTT CAGAGACAGA
HMPREF1120_06290         -    357  6.99e-06 GGCGTGATGC GTTCAAGTT GGCTGAGACG
HMPREF1120_09238         -    793  6.99e-06 ACTGTACGAC GTTCAAGTT TATTCAAACG
HMPREF1120_05234         +    234  6.99e-06 GGTGGGAGCC GTTCAAGTT AGCGACGATA
HMPREF1120_05354         -    875  6.99e-06 CTTTGCCAGC GTTCAAGTT CACTGCGTCT
HMPREF1120_05142         -    361  6.99e-06 TTTCAAGAGC GTTCAAGTT TGATGCCCCA
HMPREF1120_00375         -    958  6.99e-06 GGTCTCCAAG GTTCAAGTT GTGCATGAAT
HMPREF1120_06679         +    209  6.99e-06 ATGGAGCTAG GTTCAAGTT GGTGCTGGGT
HMPREF1120_02578         -    964  6.99e-06 TGTTCCGCAG GTTCAAGTT CAGCCTTGCC
HMPREF1120_02698         -    626  6.99e-06 GAGGCTCGTG GTTCAAGTT GAAGTTATCA
HMPREF1120_03095         -    258  6.99e-06 CGGCACTACA GTTCAAGTT CCCGGTAACC
HMPREF1120_00041         +    523  6.99e-06 GACTAAAGTC GTTCAAGTT GCGGCTGATC
HMPREF1120_09262         +    724  6.99e-06 TCGACCCGGA GTTCAAGTT CATTGTTGTT
HMPREF1120_08308         -    432  6.99e-06 GAGTGGTGTA GTTCAAGTT GGAAACCTCT
HMPREF1120_06188         +    885  6.99e-06 TCATCGACTT GTTCAAGTT GTATGGGGAA
HMPREF1120_05801         +    264  6.99e-06 TGCACCTGAG GTTCAAGTT CAATCCACAC
```

HMPREF1120_02118	+	288	6.99e-06	GCTTCTGTAT	GTTCAAGTT	TCGTTATCTA
HMPREF1120_07951	-	837	6.99e-06	GAATGTCCAC	GTTCAAGTT	TCGCGTGGAA
HMPREF1120_05644	+	917	6.99e-06	GCGTGGAGCT	GTTCAAGTT	GCGGCTGTGG
HMPREF1120_00689	+	695	6.99e-06	CCCGTTGTTT	GTTCAAGTT	AAGAGTCTAC
HMPREF1120_03835	+	45	6.99e-06	AATCCACCAG	GTTCAAGTT	CAGGGCAGGA
HMPREF1120_00627	+	950	6.99e-06	CCACTTTGCA	GTTCAAGTT	TGCGATTGAG
HMPREF1120_00342	+	137	6.99e-06	GCCAGTTCGA	GTTCAAGTT	GGATTTGGAT
HMPREF1120_01106	+	772	6.99e-06	CAATTGTGCA	GTTCAAGTT	CGCATGTCGT
HMPREF1120_04359	+	404	1.05e-05	GATGGTTCGA	CTTGAAGTT	CGACCAGCTC
HMPREF1120_03772	+	373	1.05e-05	TTGTTGTGTC	CTTGAAGTT	ACCTTTCTAA
HMPREF1120_06528	-	544	1.05e-05	AGTCCTTGGA	CTTGAAGTT	CAACGAACCT
HMPREF1120_08115	-	196	1.05e-05	GGGTTGGGTT	CTTGAAGTT	CAGTGTTGAC
HMPREF1120_02454	-	176	1.05e-05	GACCACGACA	CTTGAAGTT	GGAGCGTCCT
HMPREF1120_06671	+	639	1.05e-05	ACTGGCACGA	CTTGAAGTT	GAGCTGCCAG
HMPREF1120_01817	+	203	1.05e-05	CAGATGAAGA	CTTGAAGTT	GTAAAGAGTT
HMPREF1120_08525	+	763	1.05e-05	GTGAACTGGA	CTTGAAGTT	GGGGACAGCG
HMPREF1120_06823	+	483	1.05e-05	TTGCTGAAGA	CTTGAAGTT	CTACTTTGAG
HMPREF1120_01653	-	836	1.05e-05	AGACGTTCTA	CTTGAAGTT	GTGACCCGGG
HMPREF1120_06027	+	234	1.77e-05	CTTCGCCGTT	GTTGAAGTT	GCGTCCGATG
HMPREF1120_06617	+	638	1.77e-05	CCGTTTCTGG	CTTCAAGTG	GCGACCATCT
HMPREF1120_01923	+	75	1.77e-05	GCTGCATCAG	CTTCAAGTG	ATACCTCTTC
HMPREF1120_07608	+	501	1.77e-05	TGGCGCAAAA	GTTGAAGTT	CTGGATCGGA
HMPREF1120_03297	+	875	1.77e-05	AAATCAAATA	CTTCAAGTG	CCCCCAAGCA
HMPREF1120_08742	+	7	1.77e-05	GAATTG	GTTGAAGTT	TCTCGGTTTC
HMPREF1120_07560	+	293	1.77e-05	CAGAGTGTGA	CTTCAAGTG	GGAGGCTCCT
HMPREF1120_03846	-	894	1.77e-05	AGTTGGTGTA	CTTCAAGTG	CCATGGCCAA
HMPREF1120_08773	+	639	1.77e-05	TTATCAGAGA	GTTGAAGTT	TTGCCGGCAT
HMPREF1120_02653	-	455	1.77e-05	CCAGGTACGG	GTTGAAGTT	TGCCTGCGAT
HMPREF1120_07048	+	772	1.77e-05	AGTGGAAGTT	GTTGAAGTT	CCGGCACAAC
HMPREF1120_08684	-	232	2.14e-05	ACATCTCGTC	CTTCAAGTC	TTCGATCGAT
HMPREF1120_02127	-	812	2.14e-05	TTCCCGACGA	CTTCAAGTC	ATTCTGGACT
HMPREF1120_07908	-	635	2.14e-05	ATCTGCTCTG	CTTCAAGTC	CGCTTTACCG
HMPREF1120_00898	-	444	2.14e-05	AGTCAACCCC	CTTCAAGTC	TCTGAATGTG
HMPREF1120_00585	+	369	2.14e-05	TCTTTCAATG	CTTCAAGTC	CTCCTGGTCC
HMPREF1120_03541	-	908	2.14e-05	AACCAACTTT	CTTCAAGTC	GTGCAGTTGT
HMPREF1120_01453	-	708	2.47e-05	CTTCCGATGC	ATTCAAGTT	CCCGCTCACT
HMPREF1120_01523	+	805	2.47e-05	AATTTCTTCC	ATTCAAGTT	CGGCTTCCCG
HMPREF1120_00954	+	547	2.47e-05	CGGGTCGTCC	ATTCAAGTT	GTGTGTAGGA
HMPREF1120_06046	+	612	2.47e-05	ACTCAACGAC	ATTCAAGTT	TCCCACCTTT
HMPREF1120_05027	-	815	2.47e-05	ATGTAGTTGC	ATTCAAGTT	GCGAACTTGC
HMPREF1120_07372	+	628	2.47e-05	ATACTGACTG	ATTCAAGTT	CTGCGAGACA
HMPREF1120_03386	+	663	2.47e-05	ATATTATGGT	ATTCAAGTT	TGATTCTGGC
HMPREF1120_00717	-	774	2.47e-05	ATTCAAATTA	ATTCAAGTT	TACCATTGAT
HMPREF1120_04934	-	700	2.47e-05	CAGGACCGGC	ATTCAAGTT	GTAGACCTGC
HMPREF1120_04516	+	786	2.47e-05	CTGCGCCCAA	ATTCAAGTT	CCTTGCTCGA
HMPREF1120_04980	+	298	2.84e-05	TTACCGTATG	GTTCAAGTG	ACCAATGGGC
HMPREF1120_01777	+	573	2.84e-05	CAAAGCTGAG	GTTCAAGTG	GTGAGAAGAG
HMPREF1120_08448	-	858	2.84e-05	CGAGCGCCTT	GTTCAAGTG	CACTGTCTAC
HMPREF1120_03061	-	601	2.84e-05	AATGAACATA	GTTCAAGTG	GACAAAATTT
HMPREF1120_05848	-	810	3.21e-05	GTCATGCTTG	GTTCAAGTC	AATGCATACA
HMPREF1120_01149	+	499	3.21e-05	GATGGTTTGG	GTTCAAGTC	TGCGGCACTG
HMPREF1120_06363	-	827	3.21e-05	GAATTGTCGG	GTTCAAGTC	TGTTTGTGGT
HMPREF1120_02703	+	679	3.21e-05	GGCACCCACC	GTTCAAGTC	GTGCTCACTG
HMPREF1120_03411	+	268	3.54e-05	CAGAGCAGCC	TTTCAAGTT	GCCGGCGACG
HMPREF1120_03646	+	558	3.54e-05	CACCACCGTG	TTTCAAGTT	GTGAGTGCAA
HMPREF1120_04550	+	343	3.54e-05	ACACCTTTCC	TTTCAAGTT	GCGCAGTGCC
HMPREF1120_02566	-	454	3.54e-05	GTATTTCTGG	TTTCAAGTT	CTTCATGCTC
HMPREF1120_08439	+	256	3.91e-05	GACACCCGGC	CTTGAAGTG	AGGCTTAACA
HMPREF1120_01156	+	332	3.91e-05	CTCTTGTCCC	CTTGAAGTG	AGCTTGCACG
HMPREF1120_06138	+	675	3.91e-05	GTACGTGCCC	CTTGAAGTG	GTAAGGCGTC
HMPREF1120_06393	-	256	3.91e-05	GATACTCTTG	CTTGAAGTG	GATCGGATTG
HMPREF1120_04843	-	861	3.91e-05	GGATGTCGGG	CTTGAAGTG	TCTGTCGGCG
HMPREF1120_04574	+	731	3.91e-05	TATTTATGGC	CTTGAAGTG	TATAGTCACT
HMPREF1120_05635	-	400	3.91e-05	AAGCGACTGC	CTTGAAGTG	TCGCGAGGCT
HMPREF1120_04938	+	65	3.91e-05	ACCGCATCCG	CTTGAAGTG	TCGTCGGTCG
HMPREF1120_04140	+	363	4.28e-05	CAAAATCGGC	CTTGAAGTC	GTCCTGCGTC
HMPREF1120_07157	-	888	4.28e-05	CCGTGTGTTT	CTTGAAGTC	GCAGGTTCGG
HMPREF1120_03399	-	967	4.28e-05	AATTGAAGTG	CTTGAAGTC	GCTTTCCCGC
HMPREF1120_07533	+	695	4.28e-05	TGGGATGTGT	CTTGAAGTC	TGGATACAGA

```
HMPREF1120_04438            +    573  4.28e-05 ACCTTTGTCC CTTGAAGTC GAAGATACAC
HMPREF1120_02117            -    659  4.28e-05 TTTCCTTGGG CTTGAAGTC AGCGAGCATG
HMPREF1120_03745            -    857  4.98e-05 GAAGAAGAGG GTTGAAGTG GCCTTTAAGC
HMPREF1120_03454            +    171  4.98e-05 GAAAAGCGCA GTTGAAGTG CAGGATCTCA
HMPREF1120_09162            -    918  4.98e-05 AGTCGAACAG GTTGAAGTG GCACACAAGA
HMPREF1120_06279            +    171  4.98e-05 TGCCCAAAGG GTTGAAGTG ACCCCGGCCT
HMPREF1120_00149            -    484  4.98e-05 AAAACATCAT GTTGAAGTG GTATGCCATA
HMPREF1120_02715            -    765  4.98e-05 ACTGCACTTG GTTGAAGTG CAAGTGTGGT
HMPREF1120_01896            +    822  6.05e-05 TCCCGCAGTC CTTCAAGAT GCTTTGAAAT
HMPREF1120_06164            +    505  6.05e-05 GATCACGTGG CTTCAAGAT AGCTGACTAA
HMPREF1120_04784            -    379  6.05e-05 GGCACAGTAG CTTCAAGAT CGAGATCAAC
HMPREF1120_00621            +    220  6.38e-05 GCGTGGCTGA TTTGAAGTT GATCCTTGTT
HMPREF1120_04788            -    317  7.09e-05 GTACAGACTC CTCCAAGTT TACCGCAGCA
HMPREF1120_05260            -    279  8.86e-05 AAGGAATTTG CTTCAACTT GAGCGTAAGC
HMPREF1120_07526            +    592  8.86e-05 CAATCATTAT GTCCAAGTT GTAGGTGGTT
--------------------------------------------------------------------------------

--------------------------------------------------------------------------------
        Motif STTCAAGTT MEME-1 block diagrams
--------------------------------------------------------------------------------
SEQUENCE NAME            POSITION P-VALUE  MOTIF DIAGRAM
-------------            ----------------  -------------
HMPREF1120_01601                  3.5e-06  613_[+1]_378
HMPREF1120_05622                  3.5e-06  123_[+1]_868
HMPREF1120_02391                  3.5e-06  553_[-1]_438
HMPREF1120_02756                  3.5e-06  522_[+1]_469
HMPREF1120_03417                  3.5e-06  849_[+1]_142
HMPREF1120_03803                  3.5e-06  898_[+1]_93
HMPREF1120_08783                  3.5e-06  39_[-1]_952
HMPREF1120_07241                  3.5e-06  662_[+1]_329
HMPREF1120_05790                  3.5e-06  207_[+1]_784
HMPREF1120_02792                  3.5e-06  346_[+1]_645
HMPREF1120_06484                  3.5e-06  359_[+1]_632
HMPREF1120_02938                  3.5e-06  479_[+1]_512
HMPREF1120_06465                  3.5e-06  461_[+1]_530
HMPREF1120_07879                  3.5e-06  753_[-1]_238
HMPREF1120_00198                  3.5e-06  369_[-1]_622
HMPREF1120_01182                  3.5e-06  708_[-1]_283
HMPREF1120_04734                  3.5e-06  974_[+1]_17
HMPREF1120_03451                  3.5e-06  192_[-1]_799
HMPREF1120_01099                  3.5e-06  446_[-1]_545
HMPREF1120_00038                  3.5e-06  11_[+1]_980
HMPREF1120_07676                  3.5e-06  983_[+1]_8
HMPREF1120_01403                  3.5e-06  561_[-1]_430
HMPREF1120_04224                  3.5e-06  862_[-1]_129
HMPREF1120_06777                  3.5e-06  454_[+1]_537
HMPREF1120_00264                  3.5e-06  753_[-1]_238
HMPREF1120_06644                  3.5e-06  788_[+1]_203
HMPREF1120_09134                  3.5e-06  397_[+1]_594
HMPREF1120_07623                  3.5e-06  502_[+1]_489
HMPREF1120_09019                  3.5e-06  780_[+1]_211
HMPREF1120_02305                  3.5e-06  415_[+1]_576
HMPREF1120_06645                  3.5e-06  823_[-1]_168
HMPREF1120_02286                  3.5e-06  801_[-1]_190
HMPREF1120_03649                  3.5e-06  158_[-1]_833
HMPREF1120_02776                  3.5e-06  730_[+1]_261
HMPREF1120_08138                  3.5e-06  626_[-1]_365
HMPREF1120_08154                  3.5e-06  512_[+1]_479
HMPREF1120_01375                  3.5e-06  339_[-1]_652
HMPREF1120_01579                  3.5e-06  751_[-1]_240
HMPREF1120_08650                  3.5e-06  729_[+1]_262
HMPREF1120_02021                  3.5e-06  822_[-1]_169
HMPREF1120_02359                  3.5e-06  254_[-1]_737
HMPREF1120_01968                  3.5e-06  979_[-1]_12
HMPREF1120_07996                  3.5e-06  508_[+1]_483
HMPREF1120_02634                  3.5e-06  18_[+1]_973
HMPREF1120_00652                  3.5e-06  529_[-1]_462
HMPREF1120_06490                  3.5e-06  662_[-1]_329
HMPREF1120_05710                  3.5e-06  187_[-1]_804
```

```
HMPREF1120_07664                 3.5e-06  938_[+1]_53
HMPREF1120_01967                 3.5e-06  951_[+1]_40
HMPREF1120_05950                 3.5e-06  953_[-1]_38
HMPREF1120_07962                 3.5e-06  696_[-1]_295
HMPREF1120_02631                 3.5e-06  258_[-1]_733
HMPREF1120_06129                 3.5e-06  936_[-1]_55
HMPREF1120_07528                 3.5e-06  485_[-1]_506
HMPREF1120_00418                 3.5e-06  805_[-1]_186
HMPREF1120_08476                   7e-06  948_[-1]_43
HMPREF1120_05486                   7e-06  398_[+1]_593
HMPREF1120_07182                   7e-06  552_[-1]_439
HMPREF1120_01818                   7e-06  228_[-1]_763
HMPREF1120_00523                   7e-06  303_[+1]_688
HMPREF1120_06804                   7e-06  797_[+1]_194
HMPREF1120_01982                   7e-06  589_[-1]_402
HMPREF1120_05841                   7e-06  485_[-1]_506
HMPREF1120_00902                   7e-06  314_[+1]_677
HMPREF1120_01020                   7e-06  101_[+1]_890
HMPREF1120_03603                   7e-06  793_[-1]_198
HMPREF1120_06290                   7e-06  356_[-1]_635
HMPREF1120_09238                   7e-06  792_[-1]_199
HMPREF1120_05234                   7e-06  233_[+1]_758
HMPREF1120_05354                   7e-06  874_[-1]_117
HMPREF1120_05142                   7e-06  360_[-1]_631
HMPREF1120_00375                   7e-06  957_[-1]_34
HMPREF1120_06679                   7e-06  208_[+1]_783
HMPREF1120_02578                   7e-06  963_[-1]_28
HMPREF1120_02698                   7e-06  625_[-1]_366
HMPREF1120_03095                   7e-06  257_[-1]_734
HMPREF1120_00041                   7e-06  522_[+1]_469
HMPREF1120_09262                   7e-06  723_[+1]_268
HMPREF1120_08308                   7e-06  431_[-1]_560
HMPREF1120_06188                   7e-06  884_[+1]_107
HMPREF1120_05801                   7e-06  263_[+1]_728
HMPREF1120_02118                   7e-06  287_[+1]_704
HMPREF1120_07951                   7e-06  836_[-1]_155
HMPREF1120_05644                   7e-06  916_[+1]_75
HMPREF1120_00689                   7e-06  694_[+1]_297
HMPREF1120_03835                   7e-06  44_[+1]_947
HMPREF1120_00627                   7e-06  949_[+1]_42
HMPREF1120_00342                   7e-06  136_[+1]_855
HMPREF1120_01106                   7e-06  771_[+1]_220
HMPREF1120_04359                   1e-05  403_[+1]_588
HMPREF1120_03772                   1e-05  372_[+1]_619
HMPREF1120_06528                   1e-05  543_[-1]_448
HMPREF1120_08115                   1e-05  195_[-1]_796
HMPREF1120_02454                   1e-05  175_[-1]_816
HMPREF1120_06671                   1e-05  638_[+1]_353
HMPREF1120_01817                   1e-05  202_[+1]_789
HMPREF1120_08525                   1e-05  762_[+1]_229
HMPREF1120_06823                   1e-05  482_[+1]_509
HMPREF1120_01653                   1e-05  835_[-1]_156
HMPREF1120_06027                 1.8e-05  233_[+1]_758
HMPREF1120_06617                 1.8e-05  637_[+1]_354
HMPREF1120_01923                 1.8e-05  74_[+1]_917
HMPREF1120_07608                 1.8e-05  500_[+1]_491
HMPREF1120_03297                 1.8e-05  874_[+1]_117
HMPREF1120_08742                 1.8e-05  6_[+1]_985
HMPREF1120_07560                 1.8e-05  292_[+1]_699
HMPREF1120_03846                 1.8e-05  893_[-1]_98
HMPREF1120_08773                 1.8e-05  638_[+1]_353
HMPREF1120_02653                 1.8e-05  454_[-1]_537
HMPREF1120_07048                 1.8e-05  771_[+1]_220
HMPREF1120_08684                 2.1e-05  231_[-1]_760
HMPREF1120_02127                 2.1e-05  811_[-1]_180
HMPREF1120_07908                 2.1e-05  634_[-1]_357
HMPREF1120_00898                 2.1e-05  443_[-1]_548
HMPREF1120_00585                 2.1e-05  368_[+1]_623
HMPREF1120_03541                 2.1e-05  907_[-1]_84
```

```
HMPREF1120_01453                 2.5e-05  707_[-1]_284
HMPREF1120_01523                 2.5e-05  804_[+1]_187
HMPREF1120_00954                 2.5e-05  546_[+1]_445
HMPREF1120_06046                 2.5e-05  611_[+1]_380
HMPREF1120_05027                 2.5e-05  814_[-1]_177
HMPREF1120_07372                 2.5e-05  627_[+1]_364
HMPREF1120_03386                 2.5e-05  662_[+1]_329
HMPREF1120_00717                 2.5e-05  773_[-1]_218
HMPREF1120_04934                 2.5e-05  699_[-1]_292
HMPREF1120_04516                 2.5e-05  785_[+1]_206
HMPREF1120_04980                 2.8e-05  297_[+1]_694
HMPREF1120_01777                 2.8e-05  572_[+1]_419
HMPREF1120_08448                 2.8e-05  857_[-1]_134
HMPREF1120_03061                 2.8e-05  600_[-1]_391
HMPREF1120_05848                 3.2e-05  809_[-1]_182
HMPREF1120_01149                 3.2e-05  498_[+1]_493
HMPREF1120_06363                 3.2e-05  826_[-1]_165
HMPREF1120_02703                 3.2e-05  678_[+1]_313
HMPREF1120_03411                 3.5e-05  267_[+1]_724
HMPREF1120_03646                 3.5e-05  557_[+1]_434
HMPREF1120_04550                 3.5e-05  342_[+1]_649
HMPREF1120_02566                 3.5e-05  453_[-1]_538
HMPREF1120_08439                 3.9e-05  255_[+1]_736
HMPREF1120_01156                 3.9e-05  331_[+1]_660
HMPREF1120_06138                 3.9e-05  674_[+1]_317
HMPREF1120_06393                 3.9e-05  255_[-1]_736
HMPREF1120_04843                 3.9e-05  860_[-1]_131
HMPREF1120_04574                 3.9e-05  730_[+1]_261
HMPREF1120_05635                 3.9e-05  399_[-1]_592
HMPREF1120_04938                 3.9e-05  64_[+1]_927
HMPREF1120_04140                 4.3e-05  362_[+1]_629
HMPREF1120_07157                 4.3e-05  887_[-1]_104
HMPREF1120_03399                 4.3e-05  966_[-1]_25
HMPREF1120_07533                 4.3e-05  694_[+1]_297
HMPREF1120_04438                 4.3e-05  572_[+1]_419
HMPREF1120_02117                 4.3e-05  658_[-1]_333
HMPREF1120_03745                   5e-05  856_[-1]_135
HMPREF1120_03454                   5e-05  170_[+1]_821
HMPREF1120_09162                   5e-05  917_[-1]_74
HMPREF1120_06279                   5e-05  170_[+1]_821
HMPREF1120_00149                   5e-05  483_[-1]_508
HMPREF1120_02715                   5e-05  764_[-1]_227
HMPREF1120_01896                   6e-05  821_[+1]_170
HMPREF1120_06164                   6e-05  504_[+1]_487
HMPREF1120_04784                   6e-05  378_[-1]_613
HMPREF1120_00621                 6.4e-05  219_[+1]_772
HMPREF1120_04788                 7.1e-05  316_[-1]_675
HMPREF1120_05260                 8.9e-05  278_[-1]_713
HMPREF1120_07526                 8.9e-05  591_[+1]_400
--------------------------------------------------------------------------------

--------------------------------------------------------------------------------
        Motif STTCAAGTT MEME-1 in BLOCKS format
--------------------------------------------------------------------------------
BL   MOTIF STTCAAGTT width=9 seqs=165
HMPREF1120_01601         (  614) CTTCAAGTT  1
HMPREF1120_05622         (  124) CTTCAAGTT  1
HMPREF1120_02391         (  554) CTTCAAGTT  1
HMPREF1120_02756         (  523) CTTCAAGTT  1
HMPREF1120_03417         (  850) CTTCAAGTT  1
HMPREF1120_03803         (  899) CTTCAAGTT  1
HMPREF1120_08783         (   40) CTTCAAGTT  1
HMPREF1120_07241         (  663) CTTCAAGTT  1
HMPREF1120_05790         (  208) CTTCAAGTT  1
HMPREF1120_02792         (  347) CTTCAAGTT  1
HMPREF1120_06484         (  360) CTTCAAGTT  1
HMPREF1120_02938         (  480) CTTCAAGTT  1
HMPREF1120_06465         (  462) CTTCAAGTT  1
HMPREF1120_07879         (  754) CTTCAAGTT  1
```

```
HMPREF1120_00198         (  370) CTTCAAGTT  1
HMPREF1120_01182         (  709) CTTCAAGTT  1
HMPREF1120_04734         (  975) CTTCAAGTT  1
HMPREF1120_03451         (  193) CTTCAAGTT  1
HMPREF1120_01099         (  447) CTTCAAGTT  1
HMPREF1120_00038         (   12) CTTCAAGTT  1
HMPREF1120_07676         (  984) CTTCAAGTT  1
HMPREF1120_01403         (  562) CTTCAAGTT  1
HMPREF1120_04224         (  863) CTTCAAGTT  1
HMPREF1120_06777         (  455) CTTCAAGTT  1
HMPREF1120_00264         (  754) CTTCAAGTT  1
HMPREF1120_06644         (  789) CTTCAAGTT  1
HMPREF1120_09134         (  398) CTTCAAGTT  1
HMPREF1120_07623         (  503) CTTCAAGTT  1
HMPREF1120_09019         (  781) CTTCAAGTT  1
HMPREF1120_02305         (  416) CTTCAAGTT  1
HMPREF1120_06645         (  824) CTTCAAGTT  1
HMPREF1120_02286         (  802) CTTCAAGTT  1
HMPREF1120_03649         (  159) CTTCAAGTT  1
HMPREF1120_02776         (  731) CTTCAAGTT  1
HMPREF1120_08138         (  627) CTTCAAGTT  1
HMPREF1120_08154         (  513) CTTCAAGTT  1
HMPREF1120_01375         (  340) CTTCAAGTT  1
HMPREF1120_01579         (  752) CTTCAAGTT  1
HMPREF1120_08650         (  730) CTTCAAGTT  1
HMPREF1120_02021         (  823) CTTCAAGTT  1
HMPREF1120_02359         (  255) CTTCAAGTT  1
HMPREF1120_01968         (  980) CTTCAAGTT  1
HMPREF1120_07996         (  509) CTTCAAGTT  1
HMPREF1120_02634         (   19) CTTCAAGTT  1
HMPREF1120_00652         (  530) CTTCAAGTT  1
HMPREF1120_06490         (  663) CTTCAAGTT  1
HMPREF1120_05710         (  188) CTTCAAGTT  1
HMPREF1120_07664         (  939) CTTCAAGTT  1
HMPREF1120_01967         (  952) CTTCAAGTT  1
HMPREF1120_05950         (  954) CTTCAAGTT  1
HMPREF1120_07962         (  697) CTTCAAGTT  1
HMPREF1120_02631         (  259) CTTCAAGTT  1
HMPREF1120_06129         (  937) CTTCAAGTT  1
HMPREF1120_07528         (  486) CTTCAAGTT  1
HMPREF1120_00418         (  806) CTTCAAGTT  1
HMPREF1120_08476         (  949) GTTCAAGTT  1
HMPREF1120_05486         (  399) GTTCAAGTT  1
HMPREF1120_07182         (  553) GTTCAAGTT  1
HMPREF1120_01818         (  229) GTTCAAGTT  1
HMPREF1120_00523         (  304) GTTCAAGTT  1
HMPREF1120_06804         (  798) GTTCAAGTT  1
HMPREF1120_01982         (  590) GTTCAAGTT  1
HMPREF1120_05841         (  486) GTTCAAGTT  1
HMPREF1120_00902         (  315) GTTCAAGTT  1
HMPREF1120_01020         (  102) GTTCAAGTT  1
HMPREF1120_03603         (  794) GTTCAAGTT  1
HMPREF1120_06290         (  357) GTTCAAGTT  1
HMPREF1120_09238         (  793) GTTCAAGTT  1
HMPREF1120_05234         (  234) GTTCAAGTT  1
HMPREF1120_05354         (  875) GTTCAAGTT  1
HMPREF1120_05142         (  361) GTTCAAGTT  1
HMPREF1120_00375         (  958) GTTCAAGTT  1
HMPREF1120_06679         (  209) GTTCAAGTT  1
HMPREF1120_02578         (  964) GTTCAAGTT  1
HMPREF1120_02698         (  626) GTTCAAGTT  1
HMPREF1120_03095         (  258) GTTCAAGTT  1
HMPREF1120_00041         (  523) GTTCAAGTT  1
HMPREF1120_09262         (  724) GTTCAAGTT  1
HMPREF1120_08308         (  432) GTTCAAGTT  1
HMPREF1120_06188         (  885) GTTCAAGTT  1
HMPREF1120_05801         (  264) GTTCAAGTT  1
HMPREF1120_02118         (  288) GTTCAAGTT  1
HMPREF1120_07951         (  837) GTTCAAGTT  1
```

```
HMPREF1120_05644         (  917) GTTCAAGTT  1
HMPREF1120_00689         (  695) GTTCAAGTT  1
HMPREF1120_03835         (   45) GTTCAAGTT  1
HMPREF1120_00627         (  950) GTTCAAGTT  1
HMPREF1120_00342         (  137) GTTCAAGTT  1
HMPREF1120_01106         (  772) GTTCAAGTT  1
HMPREF1120_04359         (  404) CTTGAAGTT  1
HMPREF1120_03772         (  373) CTTGAAGTT  1
HMPREF1120_06528         (  544) CTTGAAGTT  1
HMPREF1120_08115         (  196) CTTGAAGTT  1
HMPREF1120_02454         (  176) CTTGAAGTT  1
HMPREF1120_06671         (  639) CTTGAAGTT  1
HMPREF1120_01817         (  203) CTTGAAGTT  1
HMPREF1120_08525         (  763) CTTGAAGTT  1
HMPREF1120_06823         (  483) CTTGAAGTT  1
HMPREF1120_01653         (  836) CTTGAAGTT  1
HMPREF1120_06027         (  234) GTTGAAGTT  1
HMPREF1120_06617         (  638) CTTCAAGTG  1
HMPREF1120_01923         (   75) CTTCAAGTG  1
HMPREF1120_07608         (  501) GTTGAAGTT  1
HMPREF1120_03297         (  875) CTTCAAGTG  1
HMPREF1120_08742         (    7) GTTGAAGTT  1
HMPREF1120_07560         (  293) CTTCAAGTG  1
HMPREF1120_03846         (  894) CTTCAAGTG  1
HMPREF1120_08773         (  639) GTTGAAGTT  1
HMPREF1120_02653         (  455) GTTGAAGTT  1
HMPREF1120_07048         (  772) GTTGAAGTT  1
HMPREF1120_08684         (  232) CTTCAAGTC  1
HMPREF1120_02127         (  812) CTTCAAGTC  1
HMPREF1120_07908         (  635) CTTCAAGTC  1
HMPREF1120_00898         (  444) CTTCAAGTC  1
HMPREF1120_00585         (  369) CTTCAAGTC  1
HMPREF1120_03541         (  908) CTTCAAGTC  1
HMPREF1120_01453         (  708) ATTCAAGTT  1
HMPREF1120_01523         (  805) ATTCAAGTT  1
HMPREF1120_00954         (  547) ATTCAAGTT  1
HMPREF1120_06046         (  612) ATTCAAGTT  1
HMPREF1120_05027         (  815) ATTCAAGTT  1
HMPREF1120_07372         (  628) ATTCAAGTT  1
HMPREF1120_03386         (  663) ATTCAAGTT  1
HMPREF1120_00717         (  774) ATTCAAGTT  1
HMPREF1120_04934         (  700) ATTCAAGTT  1
HMPREF1120_04516         (  786) ATTCAAGTT  1
HMPREF1120_04980         (  298) GTTCAAGTG  1
HMPREF1120_01777         (  573) GTTCAAGTG  1
HMPREF1120_08448         (  858) GTTCAAGTG  1
HMPREF1120_03061         (  601) GTTCAAGTG  1
HMPREF1120_05848         (  810) GTTCAAGTC  1
HMPREF1120_01149         (  499) GTTCAAGTC  1
HMPREF1120_06363         (  827) GTTCAAGTC  1
HMPREF1120_02703         (  679) GTTCAAGTC  1
HMPREF1120_03411         (  268) TTTCAAGTT  1
HMPREF1120_03646         (  558) TTTCAAGTT  1
HMPREF1120_04550         (  343) TTTCAAGTT  1
HMPREF1120_02566         (  454) TTTCAAGTT  1
HMPREF1120_08439         (  256) CTTGAAGTG  1
HMPREF1120_01156         (  332) CTTGAAGTG  1
HMPREF1120_06138         (  675) CTTGAAGTG  1
HMPREF1120_06393         (  256) CTTGAAGTG  1
HMPREF1120_04843         (  861) CTTGAAGTG  1
HMPREF1120_04574         (  731) CTTGAAGTG  1
HMPREF1120_05635         (  400) CTTGAAGTG  1
HMPREF1120_04938         (   65) CTTGAAGTG  1
HMPREF1120_04140         (  363) CTTGAAGTC  1
HMPREF1120_07157         (  888) CTTGAAGTC  1
HMPREF1120_03399         (  967) CTTGAAGTC  1
HMPREF1120_07533         (  695) CTTGAAGTC  1
HMPREF1120_04438         (  573) CTTGAAGTC  1
HMPREF1120_02117         (  659) CTTGAAGTC  1
```

```
HMPREF1120_03745         (  857) GTTGAAGTG  1
HMPREF1120_03454         (  171) GTTGAAGTG  1
HMPREF1120_09162         (  918) GTTGAAGTG  1
HMPREF1120_06279         (  171) GTTGAAGTG  1
HMPREF1120_00149         (  484) GTTGAAGTG  1
HMPREF1120_02715         (  765) GTTGAAGTG  1
HMPREF1120_01896         (  822) CTTCAAGAT  1
HMPREF1120_06164         (  505) CTTCAAGAT  1
HMPREF1120_04784         (  379) CTTCAAGAT  1
HMPREF1120_00621         (  220) TTTGAAGTT  1
HMPREF1120_04788         (  317) CTCCAAGTT  1
HMPREF1120_05260         (  279) CTTCAACTT  1
HMPREF1120_07526         (  592) GTCCAAGTT  1
//

--------------------------------------------------------------------------------

--------------------------------------------------------------------------------
        Motif STTCAAGTT MEME-1 position-specific scoring matrix
--------------------------------------------------------------------------------
log-odds matrix: alength= 4 w= 9 n= 463042 bayes= 12.9478 E= 8.3e-005
  -200    116     37   -300
 -1401  -1401  -1401    204
 -1401   -441  -1401    203
 -1401    159    -20  -1401
   204  -1401  -1401  -1401
   204  -1401  -1401  -1401
 -1401   -540    195  -1401
  -374  -1401  -1401    202
 -1401   -141    -88    165
--------------------------------------------------------------------------------

--------------------------------------------------------------------------------
        Motif STTCAAGTT MEME-1 position-specific probability matrix
--------------------------------------------------------------------------------
letter-probability matrix: alength= 4 w= 9 nsites= 165 E= 8.3e-005
 0.060606  0.575758  0.333333  0.030303
 0.000000  0.000000  0.000000  1.000000
 0.000000  0.012121  0.000000  0.987879
 0.000000  0.775758  0.224242  0.000000
 1.000000  0.000000  0.000000  0.000000
 1.000000  0.000000  0.000000  0.000000
 0.000000  0.006061  0.993939  0.000000
 0.018182  0.000000  0.000000  0.981818
 0.000000  0.096970  0.139394  0.763636
--------------------------------------------------------------------------------

--------------------------------------------------------------------------------
        Motif STTCAAGTT MEME-1 regular expression
--------------------------------------------------------------------------------
[CG]TT[CG]AAGTT
--------------------------------------------------------------------------------




Time 506.75 secs.

********************************************************************************


********************************************************************************
MOTIF ACTACTACTACTACTRNTACT MEME-2      width =  21  sites =  32  llr = 480  E-value = 8.2e+001
********************************************************************************
--------------------------------------------------------------------------------
        Motif ACTACTACTACTACTRNTACT MEME-2 Description
--------------------------------------------------------------------------------
Simplified        A  8::72:8:17:27:2621822
pos.-specific     C  27:16328::722a1:32:81
```

```
probability       G  :1:21::2:22:1::43:3::
matrix            T  :2a:17::9126::8:27::7

         bits    2.0              *
                 1.8   *          *
                 1.6   *          *
                 1.4 * *   * *    *     *
Relative         1.2 * *   ***    *    **
Entropy          1.0 * *  ****    **  ***
(21.6 bits)      0.8 **** *****  **** ****
                 0.6 **** *********** ****
                 0.4 **************** ****
                 0.2 **************** ****
                 0.0 ---------------------

Multilevel           ACTACTACTACTACTAGTACT
consensus               G C G   C   GCCG
sequence                             T

--------------------------------------------------------------------------------

--------------------------------------------------------------------------------
        Motif ACTACTACTACTACTRNTACT MEME-2 sites sorted by position p-value
--------------------------------------------------------------------------------
Sequence name            Strand  Start   P-value                    Site
-------------            ------  ----- ---------            ---------------------
HMPREF1120_04934             +     59  3.71e-13 TACTACTACT ACTACTACTACTACTACTACT ACTACTACTA
HMPREF1120_08377             +    666  8.15e-11 GTAGGCTACG AGTACTACTACTACTACTACT GTTCTTCGTT
HMPREF1120_01833             +    751  1.04e-10 CACAACTTCG ACTACTCCTACCACTACTACT CCTCGTGATT
HMPREF1120_03941             +    760  2.52e-10 ACTAATTAGT ATTGCTACTACTACTGATACT TGCGCGTTGG
HMPREF1120_07793             -    381  1.08e-09 ACTCGGATAT ACTACTACTAGTACTATCGCT ATTAACTACT
HMPREF1120_06748             -    701  1.45e-09 GTAGGTACCT ACTACTAGTAGTACTGGCACT GCAGTGCGAC
HMPREF1120_07658             +    784  1.65e-09 CGCGAGTGAT ACTACCACCACTACTACTACT GTGATACCAC
HMPREF1120_03151             -    205  1.14e-08 ACGCCGCTAT ACTATTACTTCTCCTAATACT CCGTAAGCGC
HMPREF1120_07182             -    660  1.55e-08 GTGCCAGAAC ACTGCCACTGCCACTGCCACT GATGCTCCAC
HMPREF1120_01156             +    747  1.73e-08 TTCGGCTCAA ATTAACCGTACTACTATTACT GAACAGGCCG
HMPREF1120_05123             -    781  2.41e-08 AGGACTTCCT ACTCCCACTACACCAACTACT GCAGTGTGCT
HMPREF1120_00038             +    943  2.99e-08 AAATATAGGT ACTAATACTACCACCATTACC ACATTCACCT
HMPREF1120_07951             -    807  2.99e-08 TTCGCGTGGA AGTAATACTACTACTAATGAT AATTGATGAT
HMPREF1120_08663             +    198  3.32e-08 TACCCCAGCG ACTACTACTGTACCAGGTACT TGTATTCTGG
HMPREF1120_06308             +    453  6.46e-08 GGTCTTGCCC ATTGGTACTACCACTGCCACT AAAATCATAG
HMPREF1120_04563             +    327  6.46e-08 TGTCCATTTG ATTGTCACTACTACTATTGCA GCCGTAGGTT
HMPREF1120_07608             -    644  7.10e-08 ACTCCTTCTT ATTATTACTCCTACTATCACT GTACAGTGTT
HMPREF1120_03603             +    749  7.80e-08 CGTCATCTCT ACGACTACTACTACTACCGCC TTATCGACCG
HMPREF1120_07996             -    750  7.80e-08 AATATTGCTT ACTCACACTATTACTAACACA ACGCCAACTA
HMPREF1120_05939             -    635  8.57e-08 GCAAACTCCT CCTCCTCCTACTACAAGTACC CGGACGGCGG
HMPREF1120_06645             -    461  9.41e-08 CTTGCGTTCC ACTGCCAGTGGAACTGTTACT GTAGGTGTTG
HMPREF1120_06393             -    664  1.47e-07 AGATAGCTGG ACTACTACAATACCTGGTAAT GTTGGGCGAA
HMPREF1120_00924             +    565  1.60e-07 CTTTCAAGCC CATACTAGTACTACAACTACA CACTGTGCGG
HMPREF1120_05354             -    898  1.89e-07 TGGCACTGGC ACTGGCACTGGCACTGGTACT GGCGCTTTGC
HMPREF1120_08480             -    170  3.33e-07 TGCTTTTTCG CTTCTTAGTACTACTAATAAT ATGGCTGACG
HMPREF1120_07322             -    289  3.60e-07 TACCCTGATA CCTACTACTGTACCTGGTGCA GCTCGTCAGC
HMPREF1120_07633             +     93  4.20e-07 ACGAGCGGAT ACTAACCCTACTGCTGGAGCT CCTCTGCGCT
HMPREF1120_07632             -    827  4.20e-07 ACGAGCGGAT ACTAACCCTACTGCTGGAGCT CCTCTGCGCT
HMPREF1120_07533             -    119  4.20e-07 CGTCTCCCCG CCTACTACAGGTACAAGTACA AGTCCAACCA
HMPREF1120_09262             -    540  4.87e-07 AACGGGTACT ACTACTCGTATCACTGGTGAC ATCAACATGA
HMPREF1120_02776             +    894  6.99e-07 ACTCCACCAC ACTACTAGTACAGCATATACA CAATCGTCTT
HMPREF1120_08344             +    717  5.74e-06 CACTATCAGG CCTGCTACTTTCCCCGTTAAT ATATACACAA
--------------------------------------------------------------------------------

--------------------------------------------------------------------------------
        Motif ACTACTACTACTACTRNTACT MEME-2 block diagrams
--------------------------------------------------------------------------------
SEQUENCE NAME            POSITION P-VALUE  MOTIF DIAGRAM
-------------            ----------------  -------------
HMPREF1120_04934                  3.7e-13  58_[+2]_921
HMPREF1120_08377                  8.2e-11  665_[+2]_314
HMPREF1120_01833                    1e-10  750_[+2]_229
HMPREF1120_03941                  2.5e-10  759_[+2]_220
```

```
HMPREF1120_07793                  1.1e-09  380_[-2]_599
HMPREF1120_06748                  1.4e-09  700_[-2]_279
HMPREF1120_07658                  1.7e-09  783_[+2]_196
HMPREF1120_03151                  1.1e-08  204_[-2]_775
HMPREF1120_07182                  1.6e-08  659_[-2]_320
HMPREF1120_01156                  1.7e-08  746_[+2]_233
HMPREF1120_05123                  2.4e-08  780_[-2]_199
HMPREF1120_00038                    3e-08  942_[+2]_37
HMPREF1120_07951                    3e-08  806_[-2]_173
HMPREF1120_08663                  3.3e-08  197_[+2]_782
HMPREF1120_06308                  6.5e-08  452_[+2]_527
HMPREF1120_04563                  6.5e-08  326_[+2]_653
HMPREF1120_07608                  7.1e-08  643_[-2]_336
HMPREF1120_03603                  7.8e-08  748_[+2]_231
HMPREF1120_07996                  7.8e-08  749_[-2]_230
HMPREF1120_05939                  8.6e-08  634_[-2]_345
HMPREF1120_06645                  9.4e-08  460_[-2]_519
HMPREF1120_06393                  1.5e-07  663_[-2]_316
HMPREF1120_00924                  1.6e-07  564_[+2]_415
HMPREF1120_05354                  1.9e-07  897_[-2]_82
HMPREF1120_08480                  3.3e-07  169_[-2]_810
HMPREF1120_07322                  3.6e-07  288_[-2]_691
HMPREF1120_07633                  4.2e-07  92_[+2]_887
HMPREF1120_07632                  4.2e-07  826_[-2]_153
HMPREF1120_07533                  4.2e-07  118_[-2]_861
HMPREF1120_09262                  4.9e-07  539_[-2]_440
HMPREF1120_02776                    7e-07  893_[+2]_86
HMPREF1120_08344                  5.7e-06  716_[+2]_263
--------------------------------------------------------------------------------

--------------------------------------------------------------------------------
        Motif ACTACTACTACTACTRNTACT MEME-2 in BLOCKS format
--------------------------------------------------------------------------------
BL   MOTIF ACTACTACTACTACTRNTACT width=21 seqs=32
HMPREF1120_04934         (   59) ACTACTACTACTACTACTACT  1
HMPREF1120_08377         (  666) AGTACTACTACTACTACTACT  1
HMPREF1120_01833         (  751) ACTACTCCTACCACTACTACT  1
HMPREF1120_03941         (  760) ATTGCTACTACTACTGATACT  1
HMPREF1120_07793         (  381) ACTACTACTAGTACTATCGCT  1
HMPREF1120_06748         (  701) ACTACTAGTAGTACTGGCACT  1
HMPREF1120_07658         (  784) ACTACCACCACTACTACTACT  1
HMPREF1120_03151         (  205) ACTATTACTTCTCCTAATACT  1
HMPREF1120_07182         (  660) ACTGCCACTGCCACTGCCACT  1
HMPREF1120_01156         (  747) ATTAACCGTACTACTATTACT  1
HMPREF1120_05123         (  781) ACTCCCACTACACCAACTACT  1
HMPREF1120_00038         (  943) ACTAATACTACCACCATTACC  1
HMPREF1120_07951         (  807) AGTAATACTACTACTAATGAT  1
HMPREF1120_08663         (  198) ACTACTACTGTACCAGGTACT  1
HMPREF1120_06308         (  453) ATTGGTACTACCACTGCCACT  1
HMPREF1120_04563         (  327) ATTGTCACTACTACTATTGCA  1
HMPREF1120_07608         (  644) ATTATTACTCCTACTATCACT  1
HMPREF1120_03603         (  749) ACGACTACTACTACTACCGCC  1
HMPREF1120_07996         (  750) ACTCACACTATTACTAACACA  1
HMPREF1120_05939         (  635) CCTCCTCCTACTACAAGTACC  1
HMPREF1120_06645         (  461) ACTGCCAGTGGAACTGTTACT  1
HMPREF1120_06393         (  664) ACTACTACAATACCTGGTAAT  1
HMPREF1120_00924         (  565) CATACTAGTACTACAACTACA  1
HMPREF1120_05354         (  898) ACTGGCACTGGCACTGGTACT  1
HMPREF1120_08480         (  170) CTTCTTAGTACTACTAATAAT  1
HMPREF1120_07322         (  289) CCTACTACTGTACCTGGTGCA  1
HMPREF1120_07633         (   93) ACTAACCCTACTGCTGGAGCT  1
HMPREF1120_07632         (  827) ACTAACCCTACTGCTGGAGCT  1
HMPREF1120_07533         (  119) CCTACTACAGGTACAAGTACA  1
HMPREF1120_09262         (  540) ACTACTCGTATCACTGGTGAC  1
HMPREF1120_02776         (  894) ACTACTAGTACAGCATATACA  1
HMPREF1120_08344         (  717) CCTGCTACTTTCCCCGTTAAT  1
//

--------------------------------------------------------------------------------
```

```
--------------------------------------------------------------------------------
	Motif ACTACTACTACTACTRNTACT MEME-2 position-specific scoring matrix
--------------------------------------------------------------------------------
log-odds matrix: alength= 4 w= 21 n= 457438 bayes= 15.8032 E= 8.2e+001 
   174    -46  -1164  -1164 
  -295    148   -204    -37 
 -1164  -1164   -304    200 
   143   -104    -23  -1164 
   -37    128   -204    -96 
 -1164     28  -1164    150 
   174    -46  -1164  -1164 
 -1164    160    -23  -1164 
  -196   -304  -1164    190 
   157   -304    -46   -196 
 -1164    135    -72    -37 
   -37    -23  -1164    129 
   157    -46   -146  -1164 
 -1164    196  -1164  -1164 
   -37   -204  -1164    163 
   121  -1164     66   -295 
   -37     13     28    -15 
  -196    -23  -1164    157 
   163  -1164     -4  -1164 
   -64    171  -1164  -1164 
   -37   -104  -1164    150 
--------------------------------------------------------------------------------

--------------------------------------------------------------------------------
	Motif ACTACTACTACTACTRNTACT MEME-2 position-specific probability matrix
--------------------------------------------------------------------------------
letter-probability matrix: alength= 4 w= 21 nsites= 32 E= 8.2e+001 
 0.812500  0.187500  0.000000  0.000000 
 0.031250  0.718750  0.062500  0.187500 
 0.000000  0.000000  0.031250  0.968750 
 0.656250  0.125000  0.218750  0.000000 
 0.187500  0.625000  0.062500  0.125000 
 0.000000  0.312500  0.000000  0.687500 
 0.812500  0.187500  0.000000  0.000000 
 0.000000  0.781250  0.218750  0.000000 
 0.062500  0.031250  0.000000  0.906250 
 0.718750  0.031250  0.187500  0.062500 
 0.000000  0.656250  0.156250  0.187500 
 0.187500  0.218750  0.000000  0.593750 
 0.718750  0.187500  0.093750  0.000000 
 0.000000  1.000000  0.000000  0.000000 
 0.187500  0.062500  0.000000  0.750000 
 0.562500  0.000000  0.406250  0.031250 
 0.187500  0.281250  0.312500  0.218750 
 0.062500  0.218750  0.000000  0.718750 
 0.750000  0.000000  0.250000  0.000000 
 0.156250  0.843750  0.000000  0.000000 
 0.187500  0.125000  0.000000  0.687500 
--------------------------------------------------------------------------------

--------------------------------------------------------------------------------
	Motif ACTACTACTACTACTRNTACT MEME-2 regular expression
--------------------------------------------------------------------------------
ACT[AG]C[TC]A[CG]TAC[TC]ACT[AG][GCT][TC][AG]CT
--------------------------------------------------------------------------------




Time 1011.74 secs.

********************************************************************************
```

```
********************************************************************************
MOTIF TYTBTKTTKBTTKBBTTBNYY MEME-3      width =  21  sites = 157  llr = 1588  E-value = 1.4e+000
********************************************************************************
--------------------------------------------------------------------------------
        Motif TYTBTKTTKBTTKBBTTBNYY MEME-3 Description
--------------------------------------------------------------------------------
Simplified        A  1:::111:::2:::1:::2:1
pos.-specific     C  1413:22214:2:342:3333
probability       G  2123:5::4322423:142:1
matrix            T  757393885266643893365

         bits    2.0
                 1.8
                 1.6     *
                 1.4     *  *        *
Relative         1.2     *  *       **
Entropy          1.0     *  *    *  **
(14.6 bits)      0.8   * * **    *  **  *
                 0.6 *** * **  ***  **  *
                 0.4 *** * *** **** *** *
                 0.2 ****************** **
                 0.0 ---------------------

Multilevel           TTTGTGTTTCTTTTCTTGCTT
consensus             C T T  GGACGCTC CTCC
sequence                C     T   GG  TA
                                       G
--------------------------------------------------------------------------------


--------------------------------------------------------------------------------
        Motif TYTBTKTTKBTTKBBTTBNYY MEME-3 sites sorted by position p-value
--------------------------------------------------------------------------------
Sequence name            Strand  Start   P-value                    Site
-------------            ------  ----- ---------            ---------------------
HMPREF1120_03803             +    406  1.68e-11 CCACAACCAC TTTGTGTTTGTTTCTTTGCTT TACAGCTTGC
HMPREF1120_02117             +    946  2.31e-08 CGTCGTCGGT TTTCTTCTTCTTTGTTTGCTT TCCCGCGTAC
HMPREF1120_07633             -    128  2.85e-08 GATTGAGGTG TTGTTGTTTGTTGTCTTTATT TCCGAGCGCA
HMPREF1120_07632             +    792  2.85e-08 GATTGAGGTG TTGTTGTTTGTTGTCTTTATT TCCGAGCGCA
HMPREF1120_06027             +    528  3.51e-08 TGTGTGTGTG TCTGTGTTTGTGTTTTTGTCC GAATCGCAGG
HMPREF1120_06028             -    923  3.51e-08 TGTGTGTGTG TCTGTGTTTGTGTTTTTGTCC GAATCGCAGG
HMPREF1120_07062             +    743  6.32e-08 AGTCCTTCCT TCTCTCTTTGTTTCTCTTCTT CGACGGCCAC
HMPREF1120_02776             +    970  7.63e-08 AAGAACTCGC TTTTTGTTTCTGTTTCTCATT CCCATCAACC
HMPREF1120_07880             -    753  1.10e-07 CCAGGGGGAC TCTGTTTTGCTTGTGCTTCTT CTCGCTCCTG
HMPREF1120_09134             +    657  1.56e-07 GCTCGTGCTT CTTCTGCTTCTTTTCTTCCTT CACGCACCAG
HMPREF1120_07560             -    509  1.56e-07 CTTGACGTCT TCTTTCTTTTTCTTCTTGGCT TCTGCGACCT
HMPREF1120_05354             +    388  1.86e-07 TCACATTTTG TTGTTGTTGTTGTTTTTTCTT CATTCGGTTT
HMPREF1120_00523             +    693  2.59e-07 CCTTCAGGCG TTTGTGTTTTGCGTTTTGTCT TACTTGGAGC
HMPREF1120_03061             +    618  2.59e-07 ACTATGTTCA TTTTTCTTTTTTTCCTTTTCA CAATCCAGAG
HMPREF1120_02274             +    345  3.58e-07 TGACCATCAT TTTGATTTTGGTTTGTTGTTT GGTTCGAAAG
HMPREF1120_02275             -    980  3.58e-07          . TTTGATTTTGGTTTGTTGTTT GGTTCGAAAG
HMPREF1120_04563             +     33  4.89e-07 TCAACCTATC TTTCTTTTTGTGTTTTGCTTT CGCCCTTTTG
HMPREF1120_07074             +    212  4.89e-07 CCTGCATTTG TTTGATTTTGATTGTTTCTTT CTGCATTGTA
HMPREF1120_05142             +    559  5.69e-07 TGGCGTCGTC TTTTTTCTTCTGGCTTTCCTT TCGACGGAAC
HMPREF1120_03399             +    841  5.69e-07 CTGGTCGGAG GCTGTGTTGGGTGTTTTCGTT GGCGTTGGCG
HMPREF1120_06528             +    920  6.61e-07 TCCAAGCTTT TGTGTATTGTTTTTGTTGCTT ATACTGCTTG
HMPREF1120_08344             +    779  6.61e-07 GCGCGTCGTT TGTTTGCTGTTTTTCTTCCTT CTCACCTACT
HMPREF1120_03768             +    718  6.61e-07 CAATTTAACT GTTGTGTCTGTTTTCCTCTTT ACTTCTTCCT
HMPREF1120_06363             +    659  8.84e-07 CTCAGTTCTA TTTCTGATTCTTGCGTTCCCC ACCGCCCGGA
HMPREF1120_07003             +    458  1.02e-06 TCAAATACGT CTTCTTTCTCATTTTTTGATT TTGTGGGAGA
HMPREF1120_08211             +    310  1.02e-06 CCTTCCAACA TTTGTGATTGATTGCTTTCCC ATGCTTACCG
HMPREF1120_00244             +    457  1.02e-06 GGGCATCGAG ATTCTTTTGCATTCCTTTGTT GGACACCGCT
HMPREF1120_05123             +    149  1.02e-06 GTTAGCCTTC CTTTTTTTTCTCGGTTTGCTC AGTAGCAGTT
HMPREF1120_00243             -    445  1.02e-06 GGGCATCGAG ATTCTTTTGCATTCCTTTGTT GGACACCGCT
HMPREF1120_05622             -    600  1.17e-06 CGATCCGAAG TCCTTTTTTCTCGCTTTTCTC ATTTCTCACT
HMPREF1120_06279             -    649  1.17e-06 TCAGATGCCT TTTGTGTCGCTTGGATTTCTT GTCCCTGGGC
HMPREF1120_00627             -    976  1.17e-06       GGTA GTGGTCTTTCTTTCTTTGTCC TTTGAGTCTC
HMPREF1120_02225             +    704  1.35e-06 AGGCTTTTTG ATTCTTTTTGGTGTTTTGATT CGTGGGTCGA
HMPREF1120_00041             +    944  1.35e-06 TATGCTTAGG GCTTTGTTTCACTCCTTTTCC GGGCTGAAAG
HMPREF1120_04377             -    186  1.54e-06 TTTCATTTCA TTTCTTTCCTTTGCCTTTCTT CTTCTTGCCT
```

```
HMPREF1120_00196         -    259  1.76e-06 GTGCAAATTG TCGTTGTTGGTTTCATTCTCT TCCTCAACCC
HMPREF1120_05842         -    289  1.76e-06 AATGAATTTG TTTGACTTTGTGGTTTTGCTC TTGCTCAAAT
HMPREF1120_03649         -    725  1.76e-06 GAAGCGGAGA TTTGTATTTTGTGTTTTGCTA CCGTACATAC
HMPREF1120_02703         +    808  1.76e-06 CGCAAACGGA TTCTTCTTTGTCGTGTTTCCT TTGTCACAAA
HMPREF1120_00717         +    373  2.01e-06 GTTTCGTCTG GCGGTGTTTCTGTTGTTCCCT ACTGTGCTTT
HMPREF1120_03202         +     81  2.29e-06 GAGCTGAGGT TCTTTTTTGCTGGGCTTGCCA ATCGGACAAT
HMPREF1120_07792         -    381  2.61e-06 TGCCGTTGTT TCGCTGTTGCTGGCTCTGCTT GACTGGAATA
HMPREF1120_05260         -    680  2.96e-06 AAGCTGTCCG TCTGTCTCTGGTTTTCTCTTT CCATGCCTCC
HMPREF1120_06393         +    226  2.96e-06 GTTGCAAGCG GCTGTGTTTGTTGGATTCATC AATCCGATCC
HMPREF1120_05935         +    122  2.96e-06 CGCCACATCG TCTTTGCTTTTCTTCTTTTCG GTCTGCGACC
HMPREF1120_06465         +    858  3.35e-06 TTTTGTCGAT TGTTTGCTGCATTCGTTTGTT CTCACTTCTT
HMPREF1120_02566         -    402  3.35e-06 GCCTATGCTC GCTTTCCTGCTTTCTTTGTCC CCAGCGAAAA
HMPREF1120_07971         +    977  3.78e-06 TCTTGATCTT TTTGTTCTCTTCTCCTTTCTC GAC
HMPREF1120_02510         -    663  3.78e-06 AGGTTATGCT TTCCTCTTTGGTGGCTTGATC GGGGGGACGA
HMPREF1120_02072         +    503  3.78e-06 CAACGGCTGA GTTCTCTTCGTTTGCTTCTCC CTCTGTGCAT
HMPREF1120_02634         +    507  3.78e-06 TCCTGGCACC TCGTTCTTGTTTTCCCTTCCT TCACCATCGT
HMPREF1120_03622         -    180  4.27e-06 TACTGACCGC GCGCTTTTGTTTTTGTTTTCC AGGGTCAACC
HMPREF1120_06441         +    959  4.27e-06 GCGCCGCGTG TTCGTGTTCGTCTTGTTCTCC GACCCTCGAG
HMPREF1120_07658         +     83  4.27e-06 TTTGGCTGTG CTTGTGTTTCTTGGCTGCTTC CTGCGCGGCT
HMPREF1120_02509         -    778  4.81e-06 ATGACCTTCC TCTTTGCTGTTTGCCCTTGTC TCGCCACAAA
HMPREF1120_08154         -    472  4.81e-06 AAGTGATTGG TCTATGATGGTTGTTTTCGTT CTAGCGCGGC
HMPREF1120_07674         +    675  4.81e-06 GCTTGAACAC TTCCTGTTTGTTGGCTGTTCT ATTTCAATGG
HMPREF1120_03503         +    475  4.81e-06 ATAGAAGCGG GCTGTTTCTCGCTTTTTGCTC TGTGTTGAGC
HMPREF1120_06777         +    919  5.41e-06 TTCAATATAA GCCCTTTTGTTCTCCTTGCTC CTTAAAGGCG
HMPREF1120_07793         -    302  5.41e-06 CTACCTAGCT TCTTTATCTATTTTCTTCTTT ATAATCGCCT
HMPREF1120_08679         +    332  5.41e-06 TGGACTCCAA TTTTTTTTGGGCGGGTTTCTG GACTGAGATA
HMPREF1120_04831         +      8  5.41e-06    CTCTTCT TCTCTGCTCTTCGGCTTGTTT GACGGGGTGG
HMPREF1120_04902         -    930  5.41e-06 TTTATGGGAT TCTATCTTTGGTTCTTTTTCC ATGAATTCAC
HMPREF1120_08078         +     65  6.80e-06 ATGTGCTGCT TTTGTCTCCTATGTCTTTGTT TTCTGGTCAT
HMPREF1120_04947         +    499  6.80e-06 CCAGGGCCGG TGGCTGTTGGGTTCCTTCATC TTGCTGACCC
HMPREF1120_07049         +    173  7.61e-06 GCGCCAGACG TCTGTCTTGGATGTCTGGACC AACAGCACAG
HMPREF1120_04834         +    321  7.61e-06 AGGAACAAAA GTTGTTTTTTTGTTGCTGTTG GTTCTATGCG
HMPREF1120_06537         -    812  7.61e-06 TGAGAAGCTG TTTATTTTGCTGGTGTTGATA TTGAGGTACT
HMPREF1120_07050         -    468  7.61e-06 GCGCCAGACG TCTGTCTTGGATGTCTGGACC AACAGCACAG
HMPREF1120_07951         +    458  7.61e-06 CGAAAACTAG TCCTAGTTGGATTTGTTCTTC AATGCCGGCA
HMPREF1120_03835         +    656  7.61e-06 CGAAAACGCC GTTCATTTTGTCTCGTTGCCC GCGGAGAGCT
HMPREF1120_03195         +    251  8.51e-06 GTTTCTGTCT TTTCTGTTCCTCTTCTGTATG CCAGCTTATT
HMPREF1120_05790         +    876  8.51e-06 AGCGGCTTTC TTCCTTTTTGACTGCCTTCTC CTCTAACCGC
HMPREF1120_04140         +    232  8.51e-06 TAGCAAGACC TTTATCTTTGTCTCTTTGGCG GCAGCCAAGA
HMPREF1120_00374         -    244  8.51e-06 CGATGGTTGC GTTGTGCTTTTCGGTTTTGCT CTTTTCGACG
HMPREF1120_02373         -    944  8.51e-06 GGTGGTTTTG TCTGTGTTGGATGGTCTTGCA AATATAGCAA
HMPREF1120_01523         +    962  9.49e-06 TCAGAGCTCG TCTCTGTTTCTCTTGTGGCAC GATTATCATT
HMPREF1120_04550         -    315  9.49e-06 AAAGGAAAGG TGTTTGTTTCGGTTCTTTTCG TCCAAATTGC
HMPREF1120_04359         -    259  1.06e-05 GCCGGGGACA TTGTTGATCCTTTCCTTGCTG TACCCTTCTA
HMPREF1120_04430         -    420  1.06e-05 CTTACTTCTC TTCTTCTTACTTTCTTTTATA CCTCTTTCTA
HMPREF1120_03772         +    653  1.06e-05 CGTGATGAAC TGTCTGTTGCAGTGCCTTGTT TCATCTCGGC
HMPREF1120_06138         -    286  1.06e-05 TGTCCTCTCT TTTCTCTTGATTTCCTCGTA CTCTACTTTT
HMPREF1120_00546         -    633  1.06e-05 TCATATCCCG ATTTTGCTTCGTTCGTTTATC ATGCATAAAT
HMPREF1120_01435         -    179  1.06e-05 TCTGTGAGCA TCTGTGTCATTTTCTCTGTCT GACAAAAATA
HMPREF1120_03541         -    583  1.06e-05 CCATCTCATC CCTCTGTTTATCTCTTTCGTC TCTCCATCTC
HMPREF1120_07322         +    963  1.18e-05 TATGTCTTTA CTTTTTCTGCTGTGTTTCTCT CTGTTATCTC
HMPREF1120_08783         -    773  1.18e-05 ACACGCGATC TCGCTGTTTCACTGCTTGAAT TAGCACTGTG
HMPREF1120_05987         -    665  1.18e-05 GACACTTACA TCTTTCTTGCTCTGGCTTTCA CCACACAATT
HMPREF1120_06548         +    643  1.18e-05 TATATTGATA CTTCTCTTGTTTTGGCTCGTC AACAGAGATG
HMPREF1120_06617         +     63  1.31e-05 GGGTGGACGG ACTGTCTTGCTTTCTCTTCTG CTGAGCGTGT
HMPREF1120_01909         +    874  1.31e-05 CAGGCATCAA TCTATGCTTGTTGCCCTGATC ACTCCATCTC
HMPREF1120_02791         +    920  1.31e-05 TTGTCGTCGC GTTCTTCTGTACTCCTTTTCT CTATGCCTTT
HMPREF1120_01547         +    568  1.31e-05 CTTCGTTCAC TCGGTGTTGCTTGGATTCGTA GGAATGCGGA
HMPREF1120_08523         +     94  1.31e-05 CCAAATGGTA GTTTTTATACATTTCTTCCTT GCTCAACTCC
HMPREF1120_02139         -    865  1.31e-05 GTCGTTCCTT TTTCTTTTGATTTCTCTCTCA CAATCATGTT
HMPREF1120_08476         -    844  1.45e-05 TTTGATGCCT GTCTTGTTTGATGCCTGTCTT GTATCACGTG
HMPREF1120_05486         +    955  1.45e-05 TGTGTGGATG CCTTTGTCGGGCTCTTTGCCT AAGCGTTCGC
HMPREF1120_07879         +    794  1.45e-05 GTTCTCTTCG TCGTTGTTGCTGGTATTGTTA CTGCTGCTTG
HMPREF1120_01764         +    311  1.45e-05 ATTTTTCGTA GCTGTGTCGTTCGTTCTGGTT ATGTCAAAAT
HMPREF1120_06580         +    784  1.45e-05 CAAAAGGTGT TTTGTCTCGCAGTGTTTGGTG GATCCGCCGC
HMPREF1120_04393         +    829  1.45e-05 TGATGCGGAA TCCCTGTTTGAGGCGCTCTTT ATGCCCGGCT
HMPREF1120_07128         -    446  1.45e-05 ACCATTTTGA TTGCAGTTGTGTTGTTTGACT GGAGTAGACT
HMPREF1120_06758         +     28  1.45e-05 TTGGATCTTA GTTCATTTGCGTTTGTTTCTA CTTGAGCAGT
HMPREF1120_06581         -    459  1.45e-05 CAAAAGGTGT TTTGTCTCGCAGTGTTTGGTG GATCCGCCGC
```

```
HMPREF1120_01967            +    352  1.45e-05 GATTGTATAC TCTCATTCTCTTTGGTTCTTG AAGTGTCTCT
HMPREF1120_07528            +    719  1.45e-05 GTCAAATCTT GTTTTAGTTCTTTCCTTCTTT CTTACCGCTT
HMPREF1120_04424            +    692  1.60e-05 ACACCTTGTC TCGTTGCTGCACTCTTTCTCC GACGGGATTT
HMPREF1120_01923            -    777  1.60e-05 TCCTCGACCT TCTCAGTTTCACTCATTCTCT CCCTTGACCT
HMPREF1120_06047            +    425  1.60e-05 ATCTAACGCC TCTCTGTTGTGTTTGTAGCTC AGGTGGATGT
HMPREF1120_03688            -    711  1.60e-05 CCCTATCTCG TTTCATTTGGTCGTGCTTGTC TGTGCCTCCT
HMPREF1120_01817            +    220  1.60e-05 TTGTAAAGAG TTGTTGATGGGTTTGTTGATG AAAGGTGGGA
HMPREF1120_08663            +    543  1.60e-05 CAAACGTCGG GTTGAGTTGCATGTTCTGACT GGTTGTCGGT
HMPREF1120_00104            +    946  1.60e-05 AGCTAATAAG GTCCTCTTGTTTTTGTGTCTT GTAGTGTAGC
HMPREF1120_07677            -     83  1.77e-05 GAAGAACGGC TGCCTCTTTCTCTCTCTCTTT GAGCAGAAAT
HMPREF1120_00924            +    824  1.77e-05 CTCCGTGCGA TCTGTTATTCTGGTGCTCATC TGAACGTTCA
HMPREF1120_05665            -    913  1.77e-05 TGACGTCATA GTGTTGATTTGTTCTTTGCTC AGCTGCCAGT
HMPREF1120_07908            +    202  1.77e-05 TCAATGTGCT CTGCTTCTGCATTCGTTGGTT TCTCCATTGT
HMPREF1120_03953            +    257  1.77e-05 GCATAATGAC CCTGTGTTACGTGTCTTGCTA CCCATTCTCT
HMPREF1120_09162            +    498  1.96e-05 TGAGATTGCA GTTCTCTTCTTTGCCTTGACG TGATTGTCCT
HMPREF1120_04224            +    207  1.96e-05 GAAGCTCTCA TCTCTATCTCAGTTCCTTATT GCATAATTTG
HMPREF1120_02454            -    567  1.96e-05 AGTGCCAATC TTTCTTTTCGTTGGGTGGATA ACATTGCACC
HMPREF1120_01082            +    605  1.96e-05 CCGCGACCCT TCTGTATCTGTTTCCTTTGAC CAAAGCCTCC
HMPREF1120_06790            -    562  1.96e-05 TGGCCGCGCT CTTCATCTTTTTTTTTCATC TTCACTCACC
HMPREF1120_00797            -    835  1.96e-05 CGAAACCACC TGCTTGTTTCATTTTCTGGTA CACCAGTACG
HMPREF1120_04804            +    701  2.16e-05 TTCAGATAGG GGTTTCTTGGGCTCTTTTCTC CTTTCCCCAT
HMPREF1120_06146            +    129  2.16e-05 AATTGAAAGT TCTCTTCTGCGTTTCTTCGAC AGCACAGTCC
HMPREF1120_08742            -    651  2.16e-05 ACGCTCGTCC TTCGTGTTTCATTCACTCCTG GCTCGCGTCT
HMPREF1120_06329            -    150  2.16e-05 AGTGAAGGCA CCCTTCTTCTTTTTGTTGCCT CCCTTCCTAC
HMPREF1120_07479            +    604  2.16e-05 TACAACATTG TGGTTGTCTTTGTCTTTGCCT TGAGCCCAGG
HMPREF1120_03417            -     63  2.38e-05 AACGACTACT TCTTTCTCTTGTGGCCTGCCT GGGTACCGGA
HMPREF1120_04574            +    500  2.38e-05 CTCTTGGTTC TTGTTATCGTATTTCTTCGCT TCTACCTCCG
HMPREF1120_01052            -    670  2.38e-05 CGCGCGTGTG TTTGTGCTTCATGTGCTCAAT CGGCGGCTCA
HMPREF1120_04126            -    340  2.38e-05 CTCGTCAAAT CCTGTTCTCGTTTGCTTGCTG CCTCTTCCTC
HMPREF1120_02172            +    592  2.38e-05 CATTCTTCAC TTTGTTCTGTACTGGCTCCCT TTTGCCTATT
HMPREF1120_01106            +     88  2.38e-05 TGACATAGCG TTCGTGTTGCAGGTTTGGGTC TTTGAGCACT
HMPREF1120_01896            +    663  2.61e-05 TAAGCCCAGT TCCGTGTCGCATGCGCTGCCT TCCGGCAAGA
HMPREF1120_07937            -    464  2.61e-05 CGGAGCTGAT TCTCTTGTTGTGGGGTTTGTT GGGCGATGCG
HMPREF1120_05100            -    135  2.61e-05 TATAACAAGG TTGGTGATGCTTGTGTTCAAT ATACGTCTAC
HMPREF1120_02305            +    958  2.61e-05 GCCATCCTCC CGTTTTTCTCTCTGTTTGTTC TTCTCATCAT
HMPREF1120_03745            +    801  2.87e-05 GTTACCACTT GCTCTGATTGATTGATTGACT GCCCGCCTTG
HMPREF1120_06583            -    957  2.87e-05 GATGCTCGGC CCTCAGTTCGATTGTTTTGTT CCTTCTTCTT
HMPREF1120_07533            +    610  2.87e-05 GTTGCTTCCT TCTTTGACTTTTTTGTGGACT CGTGTTAACT
HMPREF1120_02714            +    665  2.87e-05 GTAAATCAGA ATTGTGTTAGTTTTCTGTACT CGTGTTTTCA
HMPREF1120_04575            -    380  3.15e-05 GGAGCGAACA CTTGTGCCTGTCGGGTTGCCT TAGATCAGCA
HMPREF1120_06922            +    876  3.45e-05 ATAAAGACAT TTTGTTTTCATGGGGTTCATC AAAGTTCGGC
HMPREF1120_01315            +    623  3.45e-05 CCTGCTTCTC TCTGTATCACTCTGCTTCCCT GCTTACTGCT
HMPREF1120_00405            +    440  3.45e-05 GGCCGTGGCG ACTTTGTCTGTCTTCTTGAAT TCTGATCTAA
HMPREF1120_04938            +    443  3.45e-05 TCCGCAACCA TTCCTTCTTCTTTTGTGCATA TTTGGCAACC
HMPREF1120_00689            -    729  3.45e-05 CAGAAGTAGT TTTTTAGTTCATTCGTTGTTA GCTAGAATCC
HMPREF1120_05503            -    435  3.45e-05 CGGCTTCTTT TTCTTTTTCCACTTCCTTCCC TTTGCCCTTT
HMPREF1120_07676            -    515  3.78e-05 AGAGATTGTG GCTGTATCACTTGCTTTGGTT CGCTTCTCTA
HMPREF1120_02138            -    122  3.78e-05 GGTGCTTCAT CTTGAATTTGTTTTCTTCGCA ACAGATTAGT
HMPREF1120_08389            +    152  4.50e-05 TTGCATCACG ATGATGTTGGGTTTCTTGGTC GCGTGTCGAG
HMPREF1120_05234            -     76  4.91e-05 TGAGAGCAAG TCTGTTACGCGTGCCTTCACC ATTTCCCACG
HMPREF1120_07962            -    946  4.91e-05 TATGGAGTAT GGGGTGTTTGTGGTGTTGATG CCCACGACGA
HMPREF1120_05841            -    500  5.35e-05 TTGTATTGCT GTGTAGTTGGTTGTCTGTGTT TGTAGGTTCA
HMPREF1120_00375            +    686  8.05e-05 CTTATTGAGA TTTTTCTTTCGTGGTTACCTG GGAATAAGCT
--------------------------------------------------------------------------------

--------------------------------------------------------------------------------
        Motif TYTBTKTTKBTTKBBTTBNYY MEME-3 block diagrams
--------------------------------------------------------------------------------
SEQUENCE NAME            POSITION P-VALUE  MOTIF DIAGRAM
-------------            ----------------  -------------
HMPREF1120_03803                  1.7e-11  405_[+3]_574
HMPREF1120_02117                  2.3e-08  945_[+3]_34
HMPREF1120_07633                  2.9e-08  127_[-3]_852
HMPREF1120_07632                  2.9e-08  791_[+3]_188
HMPREF1120_06027                  3.5e-08  527_[+3]_452
HMPREF1120_06028                  3.5e-08  922_[-3]_57
HMPREF1120_07062                  6.3e-08  742_[+3]_237
HMPREF1120_02776                  7.6e-08  969_[+3]_10
HMPREF1120_07880                  1.1e-07  752_[-3]_227
```

HMPREF1120_09134	1.6e-07	656_[+3]_323
HMPREF1120_07560	1.6e-07	508_[-3]_471
HMPREF1120_05354	1.9e-07	387_[+3]_592
HMPREF1120_00523	2.6e-07	692_[+3]_287
HMPREF1120_03061	2.6e-07	617_[+3]_362
HMPREF1120_02274	3.6e-07	344_[+3]_635
HMPREF1120_02275	3.6e-07	979_[-3]
HMPREF1120_04563	4.9e-07	32_[+3]_947
HMPREF1120_07074	4.9e-07	211_[+3]_768
HMPREF1120_05142	5.7e-07	558_[+3]_421
HMPREF1120_03399	5.7e-07	840_[+3]_139
HMPREF1120_06528	6.6e-07	919_[+3]_60
HMPREF1120_08344	6.6e-07	778_[+3]_201
HMPREF1120_03768	6.6e-07	717_[+3]_262
HMPREF1120_06363	8.8e-07	658_[+3]_321
HMPREF1120_07003	1e-06	457_[+3]_522
HMPREF1120_08211	1e-06	309_[+3]_670
HMPREF1120_00244	1e-06	456_[+3]_523
HMPREF1120_05123	1e-06	148_[+3]_831
HMPREF1120_00243	1e-06	444_[-3]_535
HMPREF1120_05622	1.2e-06	599_[-3]_380
HMPREF1120_06279	1.2e-06	648_[-3]_331
HMPREF1120_00627	1.2e-06	975_[-3]_4
HMPREF1120_02225	1.3e-06	703_[+3]_276
HMPREF1120_00041	1.3e-06	943_[+3]_36
HMPREF1120_04377	1.5e-06	185_[-3]_794
HMPREF1120_00196	1.8e-06	258_[-3]_721
HMPREF1120_05842	1.8e-06	288_[-3]_691
HMPREF1120_03649	1.8e-06	724_[-3]_255
HMPREF1120_02703	1.8e-06	807_[+3]_172
HMPREF1120_00717	2e-06	372_[+3]_607
HMPREF1120_03202	2.3e-06	80_[+3]_899
HMPREF1120_07792	2.6e-06	380_[-3]_599
HMPREF1120_05260	3e-06	679_[-3]_300
HMPREF1120_06393	3e-06	225_[+3]_754
HMPREF1120_05935	3e-06	121_[+3]_858
HMPREF1120_06465	3.3e-06	857_[+3]_122
HMPREF1120_02566	3.3e-06	401_[-3]_578
HMPREF1120_07971	3.8e-06	976_[+3]_3
HMPREF1120_02510	3.8e-06	662_[-3]_317
HMPREF1120_02072	3.8e-06	502_[+3]_477
HMPREF1120_02634	3.8e-06	506_[+3]_473
HMPREF1120_03622	4.3e-06	179_[-3]_800
HMPREF1120_06441	4.3e-06	958_[+3]_21
HMPREF1120_07658	4.3e-06	82_[+3]_897
HMPREF1120_02509	4.8e-06	777_[-3]_202
HMPREF1120_08154	4.8e-06	471_[-3]_508
HMPREF1120_07674	4.8e-06	674_[+3]_305
HMPREF1120_03503	4.8e-06	474_[+3]_505
HMPREF1120_06777	5.4e-06	918_[+3]_61
HMPREF1120_07793	5.4e-06	301_[-3]_678
HMPREF1120_08679	5.4e-06	331_[+3]_648
HMPREF1120_04831	5.4e-06	7_[+3]_972
HMPREF1120_04902	5.4e-06	929_[-3]_50
HMPREF1120_08078	6.8e-06	64_[+3]_915
HMPREF1120_04947	6.8e-06	498_[+3]_481
HMPREF1120_07049	7.6e-06	172_[+3]_807
HMPREF1120_04834	7.6e-06	320_[+3]_659
HMPREF1120_06537	7.6e-06	811_[-3]_168
HMPREF1120_07050	7.6e-06	467_[-3]_512
HMPREF1120_07951	7.6e-06	457_[+3]_522
HMPREF1120_03835	7.6e-06	655_[+3]_324
HMPREF1120_03195	8.5e-06	250_[+3]_729
HMPREF1120_05790	8.5e-06	875_[+3]_104
HMPREF1120_04140	8.5e-06	231_[+3]_748
HMPREF1120_00374	8.5e-06	243_[-3]_736
HMPREF1120_02373	8.5e-06	943_[-3]_36
HMPREF1120_01523	9.5e-06	961_[+3]_18
HMPREF1120_04550	9.5e-06	314_[-3]_665

```
HMPREF1120_04359             1.1e-05  258_[-3]_721
HMPREF1120_04430             1.1e-05  419_[-3]_560
HMPREF1120_03772             1.1e-05  652_[+3]_327
HMPREF1120_06138             1.1e-05  285_[-3]_694
HMPREF1120_00546             1.1e-05  632_[-3]_347
HMPREF1120_01435             1.1e-05  178_[-3]_801
HMPREF1120_03541             1.1e-05  582_[-3]_397
HMPREF1120_07322             1.2e-05  962_[+3]_17
HMPREF1120_08783             1.2e-05  772_[-3]_207
HMPREF1120_05987             1.2e-05  664_[-3]_315
HMPREF1120_06548             1.2e-05  642_[+3]_337
HMPREF1120_06617             1.3e-05  62_[+3]_917
HMPREF1120_01909             1.3e-05  873_[+3]_106
HMPREF1120_02791             1.3e-05  919_[+3]_60
HMPREF1120_01547             1.3e-05  567_[+3]_412
HMPREF1120_08523             1.3e-05  93_[+3]_886
HMPREF1120_02139             1.3e-05  864_[-3]_115
HMPREF1120_08476             1.4e-05  843_[-3]_136
HMPREF1120_05486             1.4e-05  954_[+3]_25
HMPREF1120_07879             1.4e-05  793_[+3]_186
HMPREF1120_01764             1.4e-05  310_[+3]_669
HMPREF1120_06580             1.4e-05  783_[+3]_196
HMPREF1120_04393             1.4e-05  828_[+3]_151
HMPREF1120_07128             1.4e-05  445_[-3]_534
HMPREF1120_06758             1.4e-05  27_[+3]_952
HMPREF1120_06581             1.4e-05  458_[-3]_521
HMPREF1120_01967             1.4e-05  351_[+3]_628
HMPREF1120_07528             1.4e-05  718_[+3]_261
HMPREF1120_04424             1.6e-05  691_[+3]_288
HMPREF1120_01923             1.6e-05  776_[-3]_203
HMPREF1120_06047             1.6e-05  424_[+3]_555
HMPREF1120_03688             1.6e-05  710_[-3]_269
HMPREF1120_01817             1.6e-05  219_[+3]_760
HMPREF1120_08663             1.6e-05  542_[+3]_437
HMPREF1120_00104             1.6e-05  945_[+3]_34
HMPREF1120_07677             1.8e-05  82_[-3]_897
HMPREF1120_00924             1.8e-05  823_[+3]_156
HMPREF1120_05665             1.8e-05  912_[-3]_67
HMPREF1120_07908             1.8e-05  201_[+3]_778
HMPREF1120_03953             1.8e-05  256_[+3]_723
HMPREF1120_09162               2e-05  497_[+3]_482
HMPREF1120_04224               2e-05  206_[+3]_773
HMPREF1120_02454               2e-05  566_[-3]_413
HMPREF1120_01082               2e-05  604_[+3]_375
HMPREF1120_06790               2e-05  561_[-3]_418
HMPREF1120_00797               2e-05  834_[-3]_145
HMPREF1120_04804             2.2e-05  700_[+3]_279
HMPREF1120_06146             2.2e-05  128_[+3]_851
HMPREF1120_08742             2.2e-05  650_[-3]_329
HMPREF1120_06329             2.2e-05  149_[-3]_830
HMPREF1120_07479             2.2e-05  603_[+3]_376
HMPREF1120_03417             2.4e-05  62_[-3]_917
HMPREF1120_04574             2.4e-05  499_[+3]_480
HMPREF1120_01052             2.4e-05  669_[-3]_310
HMPREF1120_04126             2.4e-05  339_[-3]_640
HMPREF1120_02172             2.4e-05  591_[+3]_388
HMPREF1120_01106             2.4e-05  87_[+3]_892
HMPREF1120_01896             2.6e-05  662_[+3]_317
HMPREF1120_07937             2.6e-05  463_[-3]_516
HMPREF1120_05100             2.6e-05  134_[-3]_845
HMPREF1120_02305             2.6e-05  957_[+3]_22
HMPREF1120_03745             2.9e-05  800_[+3]_179
HMPREF1120_06583             2.9e-05  956_[-3]_23
HMPREF1120_07533             2.9e-05  609_[+3]_370
HMPREF1120_02714             2.9e-05  664_[+3]_315
HMPREF1120_04575             3.1e-05  379_[-3]_600
HMPREF1120_06922             3.5e-05  875_[+3]_104
HMPREF1120_01315             3.5e-05  622_[+3]_357
HMPREF1120_00405             3.5e-05  439_[+3]_540
```

```
HMPREF1120_04938                  3.5e-05  442_[+3]_537
HMPREF1120_00689                  3.5e-05  728_[-3]_251
HMPREF1120_05503                  3.5e-05  434_[-3]_545
HMPREF1120_07676                  3.8e-05  514_[-3]_465
HMPREF1120_02138                  3.8e-05  121_[-3]_858
HMPREF1120_08389                  4.5e-05  151_[+3]_828
HMPREF1120_05234                  4.9e-05  75_[-3]_904
HMPREF1120_07962                  4.9e-05  945_[-3]_34
HMPREF1120_05841                  5.3e-05  499_[-3]_480
HMPREF1120_00375                  8.1e-05  685_[+3]_294
--------------------------------------------------------------------------------

--------------------------------------------------------------------------------
        Motif TYTBTKTTKBTTKBBTTBNYY MEME-3 in BLOCKS format
--------------------------------------------------------------------------------
BL   MOTIF TYTBTKTTKBTTKBBTTBNYY width=21 seqs=157
HMPREF1120_03803         (  406) TTTGTGTTTGTTTCTTTGCTT  1
HMPREF1120_02117         (  946) TTTCTTCTTCTTTGTTTGCTT  1
HMPREF1120_07633         (  128) TTGTTGTTTGTTGTCTTTATT  1
HMPREF1120_07632         (  792) TTGTTGTTTGTTGTCTTTATT  1
HMPREF1120_06027         (  528) TCTGTGTTTGTGTTTTTGTCC  1
HMPREF1120_06028         (  923) TCTGTGTTTGTGTTTTTGTCC  1
HMPREF1120_07062         (  743) TCTCTCTTTGTTTCTCTTCTT  1
HMPREF1120_02776         (  970) TTTTTGTTTCTGTTTCTCATT  1
HMPREF1120_07880         (  753) TCTGTTTTGCTTGTGCTTCTT  1
HMPREF1120_09134         (  657) CTTCTGCTTCTTTTCTTCCTT  1
HMPREF1120_07560         (  509) TCTTTCTTTTTCTTCTTGGCT  1
HMPREF1120_05354         (  388) TTGTTGTTGTTGTTTTTTCTT  1
HMPREF1120_00523         (  693) TTTGTGTTTTGCGTTTTGTCT  1
HMPREF1120_03061         (  618) TTTTTCTTTTTTTTCCTTTCA  1
HMPREF1120_02274         (  345) TTTGATTTTGGTTTGTTGTTT  1
HMPREF1120_02275         (  980) TTTGATTTTGGTTTGTTGTTT  1
HMPREF1120_04563         (   33) TTTCTTTTTGTGTTTTGCTTT  1
HMPREF1120_07074         (  212) TTTGATTTTGATTGTTTCTTT  1
HMPREF1120_05142         (  559) TTTTTTCTTCTGGCTTTCCTT  1
HMPREF1120_03399         (  841) GCTGTGTTGGGTGTTTTCGTT  1
HMPREF1120_06528         (  920) TGTGTATTGTTTTTGTTGCTT  1
HMPREF1120_08344         (  779) TGTTTGCTGTTTTTCTTCCTT  1
HMPREF1120_03768         (  718) GTTGTGTCTGTTTTCCTCTTT  1
HMPREF1120_06363         (  659) TTTCTGATTCTTGCGTTCCCC  1
HMPREF1120_07003         (  458) CTTCTTTCTCATTTTTTGATT  1
HMPREF1120_08211         (  310) TTTGTGATTGATTGCTTTCCC  1
HMPREF1120_00244         (  457) ATTCTTTTGCATTCCTTTGTT  1
HMPREF1120_05123         (  149) CTTTTTTTTCTCGGTTTGCTC  1
HMPREF1120_00243         (  445) ATTCTTTTGCATTCCTTTGTT  1
HMPREF1120_05622         (  600) TCCTTTTTTCTCGCTTTTCTC  1
HMPREF1120_06279         (  649) TTTGTGTCGCTTGGATTTCTT  1
HMPREF1120_00627         (  976) GTGGTCTTTCTTTCTTTGTCC  1
HMPREF1120_02225         (  704) ATTCTTTTTGGTGTTTTGATT  1
HMPREF1120_00041         (  944) GCTTTGTTTCACTCCTTTTCC  1
HMPREF1120_04377         (  186) TTTCTTTCCTTTGCCTTTCTT  1
HMPREF1120_00196         (  259) TCGTTGTTGGTTTCATTCTCT  1
HMPREF1120_05842         (  289) TTTGACTTTGTGGTTTTGCTC  1
HMPREF1120_03649         (  725) TTTGTATTTTGTGTTTTGCTA  1
HMPREF1120_02703         (  808) TTCTTCTTTGTCGTGTTTCCT  1
HMPREF1120_00717         (  373) GCGGTGTTTCTGTTGTTCCCT  1
HMPREF1120_03202         (   81) TCTTTTTTGCTGGGCTTGCCA  1
HMPREF1120_07792         (  381) TCGCTGTTGCTGGCTCTGCTT  1
HMPREF1120_05260         (  680) TCTGTCTCTGGTTTTCTCTTT  1
HMPREF1120_06393         (  226) GCTGTGTTTGTTGGATTCATC  1
HMPREF1120_05935         (  122) TCTTTGCTTTTCTTCTTTTCG  1
HMPREF1120_06465         (  858) TGTTTGCTGCATTCGTTTGTT  1
HMPREF1120_02566         (  402) GCTTTCCTGCTTTCTTTGTCC  1
HMPREF1120_07971         (  977) TTTGTTCTCTTCTCCTTTCTC  1
HMPREF1120_02510         (  663) TTCCTCTTTGGTGGCTTGATC  1
HMPREF1120_02072         (  503) GTTCTCTTCGTTTGCTTCTCC  1
HMPREF1120_02634         (  507) TCGTTCTTGTTTTCCCTTCCT  1
HMPREF1120_03622         (  180) GCGCTTTTGTTTTTGTTTTCC  1
HMPREF1120_06441         (  959) TTCGTGTTCGTCTTGTTCTCC  1
```

```
HMPREF1120_07658         (   83) CTTGTGTTTCTTGGCTGCTTC  1
HMPREF1120_02509         (  778) TCTTTGCTGTTTGCCCTTGTC  1
HMPREF1120_08154         (  472) TCTATGATGGTTGTTTTCGTT  1
HMPREF1120_07674         (  675) TTCCTGTTTGTTGGCTGTTCT  1
HMPREF1120_03503         (  475) GCTGTTTCTCGCTTTTTGCTC  1
HMPREF1120_06777         (  919) GCCCTTTTGTTCTCCTTGCTC  1
HMPREF1120_07793         (  302) TCTTTATCTATTTTCTTCTTT  1
HMPREF1120_08679         (  332) TTTTTTTTGGGCGGGTTTCTG  1
HMPREF1120_04831         (    8) TCTCTGCTCTTCGGCTTGTTT  1
HMPREF1120_04902         (  930) TCTATCTTTGGTTCTTTTTCC  1
HMPREF1120_08078         (   65) TTTGTCTCCTATGTCTTTGTT  1
HMPREF1120_04947         (  499) TGGCTGTTGGGTTCCTTCATC  1
HMPREF1120_07049         (  173) TCTGTCTTGGATGTCTGGACC  1
HMPREF1120_04834         (  321) GTTGTTTTTTTGTTGCTGTTG  1
HMPREF1120_06537         (  812) TTTATTTTGCTGGTGTTGATA  1
HMPREF1120_07050         (  468) TCTGTCTTGGATGTCTGGACC  1
HMPREF1120_07951         (  458) TCCTAGTTGGATTTGTTCTTC  1
HMPREF1120_03835         (  656) GTTCATTTTGTCTCGTTGCCC  1
HMPREF1120_03195         (  251) TTTCTGTTCCTCTTCTGTATG  1
HMPREF1120_05790         (  876) TTCCTTTTTGACTGCCTTCTC  1
HMPREF1120_04140         (  232) TTTATCTTTGTCTCTTTGGCG  1
HMPREF1120_00374         (  244) GTTGTGCTTTTCGGTTTTGCT  1
HMPREF1120_02373         (  944) TCTGTGTTGGATGGTCTTGCA  1
HMPREF1120_01523         (  962) TCTCTGTTTCTCTTGTGGCAC  1
HMPREF1120_04550         (  315) TGTTTGTTTCGGTTCTTTTCG  1
HMPREF1120_04359         (  259) TTGTTGATCCTTTCCTTGCTG  1
HMPREF1120_04430         (  420) TTCTTCTTACTTTCTTTTATA  1
HMPREF1120_03772         (  653) TGTCTGTTGCAGTGCCTTGTT  1
HMPREF1120_06138         (  286) TTTCTCTTGATTTTCCTCGTA  1
HMPREF1120_00546         (  633) ATTTTGCTTCGTTCGTTTATC  1
HMPREF1120_01435         (  179) TCTGTGTCATTTTCTCTGTCT  1
HMPREF1120_03541         (  583) CCTCTGTTTATCTCTTTCGTC  1
HMPREF1120_07322         (  963) CTTTTTCTGCTGTGTTTCTCT  1
HMPREF1120_08783         (  773) TCGCTGTTTCACTGCTTGAAT  1
HMPREF1120_05987         (  665) TCTTTCTTGCTCTGGCTTTCA  1
HMPREF1120_06548         (  643) CTTCTCTTGTTTTGGCTCGTC  1
HMPREF1120_06617         (   63) ACTGTCTTGCTTTCTCTTCTG  1
HMPREF1120_01909         (  874) TCTATGCTTGTTGCCCTGATC  1
HMPREF1120_02791         (  920) GTTCTTCTGTACTCCTTTTCT  1
HMPREF1120_01547         (  568) TCGGTGTTGCTTGGATTCGTA  1
HMPREF1120_08523         (   94) GTTTTTATACATTTCTTCCTT  1
HMPREF1120_02139         (  865) TTTCTTTTGATTTCTCTCTCA  1
HMPREF1120_08476         (  844) GTCTTGTTTGATGCCTGTCTT  1
HMPREF1120_05486         (  955) CCTTTGTCGGGCTCTTTGCCT  1
HMPREF1120_07879         (  794) TCGTTGTTGCTGGTATTGTTA  1
HMPREF1120_01764         (  311) GCTGTGTCGTTCGTTCTGGTT  1
HMPREF1120_06580         (  784) TTTGTCTCGCAGTGTTTGGTG  1
HMPREF1120_04393         (  829) TCCCTGTTTGAGGCGCTCTTT  1
HMPREF1120_07128         (  446) TTGCAGTTGTGTTGTTTGACT  1
HMPREF1120_06758         (   28) GTTCATTTGCGTTTGTTTCTA  1
HMPREF1120_06581         (  459) TTTGTCTCGCAGTGTTTGGTG  1
HMPREF1120_01967         (  352) TCTCATTCTCTTTGGTTCTTG  1
HMPREF1120_07528         (  719) GTTTTAGTTCTTTCCTTCTTT  1
HMPREF1120_04424         (  692) TCGTTGCTGCACTCTTTCTCC  1
HMPREF1120_01923         (  777) TCTCAGTTTCACTCATTCTCT  1
HMPREF1120_06047         (  425) TCTCTGTTGTGTTTGTAGCTC  1
HMPREF1120_03688         (  711) TTTCATTTGGTCGTGCTTGTC  1
HMPREF1120_01817         (  220) TTGTTGATGGGTTTGTTGATG  1
HMPREF1120_08663         (  543) GTTGAGTTGCATGTTCTGACT  1
HMPREF1120_00104         (  946) GTCCTCTTGTTTTTGTGTCTT  1
HMPREF1120_07677         (   83) TGCCTCTTTCTCTCTCTCTTT  1
HMPREF1120_00924         (  824) TCTGTTATTCTGGTGCTCATC  1
HMPREF1120_05665         (  913) GTGTTGATTTGTTCTTTGCTC  1
HMPREF1120_07908         (  202) CTGCTTCTGCATTCGTTGGTT  1
HMPREF1120_03953         (  257) CCTGTGTTACGTGTCTTGCTA  1
HMPREF1120_09162         (  498) GTTCTCTTCTTTGCCTTGACG  1
HMPREF1120_04224         (  207) TCTCTATCTCAGTTCCTTATT  1
HMPREF1120_02454         (  567) TTTCTTTTCGTTGGGTGGATA  1
HMPREF1120_01082         (  605) TCTGTATCTGTTTCCTTTGAC  1
```

```
HMPREF1120_06790         (  562) CTTCATCTTTTTTTTTTCATC  1
HMPREF1120_00797         (  835) TGCTTGTTTCATTTTCTGGTA  1
HMPREF1120_04804         (  701) GGTTTCTTGGGCTCTTTTCTC  1
HMPREF1120_06146         (  129) TCTCTTCTGCGTTTCTTCGAC  1
HMPREF1120_08742         (  651) TTCGTGTTTCATTCACTCCTG  1
HMPREF1120_06329         (  150) CCCTTCTTCTTTTTGTTGCCT  1
HMPREF1120_07479         (  604) TGGTTGTCTTTGTCTTTGCCT  1
HMPREF1120_03417         (   63) TCTTTCTCTTGTGGCCTGCCT  1
HMPREF1120_04574         (  500) TTGTTATCGTATTTCTTCGCT  1
HMPREF1120_01052         (  670) TTTGTGCTTCATGTGCTCAAT  1
HMPREF1120_04126         (  340) CCTGTTCTCGTTTGCTTGCTG  1
HMPREF1120_02172         (  592) TTTGTTCTGTACTGGCTCCCT  1
HMPREF1120_01106         (   88) TTCGTGTTGCAGGTTTGGGTC  1
HMPREF1120_01896         (  663) TCCGTGTCGCATGCGCTGCCT  1
HMPREF1120_07937         (  464) TCTCTTGTTGTGGGGTTTGTT  1
HMPREF1120_05100         (  135) TTGGTGATGCTTGTGTTCAAT  1
HMPREF1120_02305         (  958) CGTTTTTCTCTCTGTTTGTTC  1
HMPREF1120_03745         (  801) GCTCTGATTGATTGATTGACT  1
HMPREF1120_06583         (  957) CCTCAGTTCGATTGTTTTGTT  1
HMPREF1120_07533         (  610) TCTTTGACTTTTTTGTGGACT  1
HMPREF1120_02714         (  665) ATTGTGTTAGTTTTCTGTACT  1
HMPREF1120_04575         (  380) CTTGTGCCTGTCGGGTTGCCT  1
HMPREF1120_06922         (  876) TTTGTTTTCATGGGGTTCATC  1
HMPREF1120_01315         (  623) TCTGTATCACTCTGCTTCCCT  1
HMPREF1120_00405         (  440) ACTTTGTCTGTCTTCTTGAAT  1
HMPREF1120_04938         (  443) TTCCTTCTTCTTTTGTGCATA  1
HMPREF1120_00689         (  729) TTTTTAGTTCATTCGTTGTTA  1
HMPREF1120_05503         (  435) TTCTTTTTCCACTTCCTTCCC  1
HMPREF1120_07676         (  515) GCTGTATCACTTGCTTTGGTT  1
HMPREF1120_02138         (  122) CTTGAATTTGTTTTCTTCGCA  1
HMPREF1120_08389         (  152) ATGATGTTGGGTTTCTTGGTC  1
HMPREF1120_05234         (   76) TCTGTTACGCGTGCCTTCACC  1
HMPREF1120_07962         (  946) GGGGTGTTTGTGGTGTTGATG  1
HMPREF1120_05841         (  500) GTGTAGTTGGTTGTCTGTGTT  1
HMPREF1120_00375         (  686) TTTTTCTTTCGTGGTTACCTG  1
//

--------------------------------------------------------------------------------

--------------------------------------------------------------------------------
        Motif TYTBTKTTKBTTKBBTTBNYY MEME-3 position-specific scoring matrix
--------------------------------------------------------------------------------
log-odds matrix: alength= 4 w= 21 n= 457438 bayes= 12.9701 E= 1.4e+000 
  -225   -125    -48    143 
 -1394     62   -175    112 
 -1394   -101    -69    156 
  -267     25     42     36 
  -125  -1394  -1394    189 
  -179    -43     83     21 
  -167    -75   -375    163 
 -1394    -58  -1394    177 
  -244   -143     52    101 
  -293     66     42    -16 
    -4  -1394    -58    129 
 -1394    -17    -64    132 
 -1394  -1394     47    141 
 -1394     31     -9     86 
  -225     47      2     45 
 -1394    -29  -1394    170 
  -425  -1394   -143    188 
 -1394     22     64     30 
   -16     34    -34      7 
  -244     39  -1394    135 
  -108     22   -125     98 
--------------------------------------------------------------------------------

--------------------------------------------------------------------------------
        Motif TYTBTKTTKBTTKBBTTBNYY MEME-3 position-specific probability matrix
--------------------------------------------------------------------------------
```

```
letter-probability matrix: alength= 4 w= 21 nsites= 157 E= 1.4e+000
 0.050955  0.108280  0.184713  0.656051
 0.000000  0.394904  0.076433  0.528662
 0.000000  0.127389  0.159236  0.713376
 0.038217  0.305732  0.343949  0.312102
 0.101911  0.000000  0.000000  0.898089
 0.070064  0.191083  0.458599  0.280255
 0.076433  0.152866  0.019108  0.751592
 0.000000  0.171975  0.000000  0.828025
 0.044586  0.095541  0.369427  0.490446
 0.031847  0.407643  0.343949  0.216561
 0.235669  0.000000  0.171975  0.592357
 0.000000  0.229299  0.165605  0.605096
 0.000000  0.000000  0.356688  0.643312
 0.000000  0.318471  0.242038  0.439490
 0.050955  0.356688  0.261146  0.331210
 0.000000  0.210191  0.000000  0.789809
 0.012739  0.000000  0.095541  0.891720
 0.000000  0.299363  0.401274  0.299363
 0.216561  0.324841  0.203822  0.254777
 0.044586  0.337580  0.000000  0.617834
 0.114650  0.299363  0.108280  0.477707
--------------------------------------------------------------------------------

--------------------------------------------------------------------------------
        Motif TYTBTKTTKBTTKBBTTBNYY MEME-3 regular expression
--------------------------------------------------------------------------------
T[TC]T[GTC]T[GT]TT[TG][CGT][TA][TC][TG][TCG][CTG][TC]T[GCT][CTAG][TC][TC]
--------------------------------------------------------------------------------




Time 1513.35 secs.

********************************************************************************


********************************************************************************
MOTIF CCAWAKGTASSTAGGTARGY MEME-4       width =  20  sites =   9  llr = 165  E-value = 2.8e+002
********************************************************************************
--------------------------------------------------------------------------------
        Motif CCAWAKGTASSTAGGTARGY MEME-4 Description
--------------------------------------------------------------------------------
Simplified        A  118671::a:::a1::93::
pos.-specific     C  781:111::33:::::::14
probability       G  111::39::77::8a::79:
matrix            T  1::424:a:::a:1:a1::6

         bits    2.0        **  ** **
                 1.8        **  ** **
                 1.6        **  ** ***
                 1.4       ***  ** *** *
Relative         1.2       ***  ** *** *
Entropy          1.0  ***  **************
(26.4 bits)      0.8  **** **************
                 0.6 ***** **************
                 0.4 ***** **************
                 0.2 ********************
                 0.0 --------------------

Multilevel           CCAAATGTAGGTAGGTAGGT
consensus               TTG   CC      A C
sequence

--------------------------------------------------------------------------------

--------------------------------------------------------------------------------
        Motif CCAWAKGTASSTAGGTARGY MEME-4 sites sorted by position p-value
```

```
--------------------------------------------------------------------------------
Sequence name            Strand  Start   P-value                    Site
-------------            ------  ----- ---------            --------------------
HMPREF1120_04947             +    676  8.78e-12 GTATACGGAC CCAAAGGTAGGTAGGTAGGC AGGCAGAAGC
HMPREF1120_02531             +    687  8.12e-11 AGTAAGCCTC CCATAAGTAGGTAGGTAGGC ATCCAGCTTG
HMPREF1120_02776             +    388  1.20e-10 TATTGTTCGA CCAAATGTACCTAGGTAAGC AATTCACTAC
HMPREF1120_02904             +    521  1.12e-09 GTCTTAGCTA CCCTAGGTACCTAGGTAGGT ACAGGCACCA
HMPREF1120_06974             -    669  1.49e-09 AGCCTGTACT ACAATTGTACCTAGGTAGGT ACCTTAGTTA
HMPREF1120_06129             -      5  4.92e-09 CGAGAGGTAC GCAATTGTAGGTAAGTAAGT ATGC
HMPREF1120_06148             -    863  6.49e-09 ATGTCTTTAG CAATACCTAGGTAGGTAGGT ACTGTACAGC
HMPREF1120_07017             +    513  1.04e-08 AAGGCAGTCC TGGTAGGTAGGTAGGTAGGT TCGGATATTC
HMPREF1120_06047             +    719  5.63e-08 ATTGAAATCA CCAACTGTAGGTATGTTACC ACGATGTCGT
--------------------------------------------------------------------------------

--------------------------------------------------------------------------------
        Motif CCAWAKGTASSTAGGTARGY MEME-4 block diagrams
--------------------------------------------------------------------------------
SEQUENCE NAME            POSITION P-VALUE  MOTIF DIAGRAM
-------------            ----------------  -------------
HMPREF1120_04947                  8.8e-12  675_[+4]_305
HMPREF1120_02531                  8.1e-11  686_[+4]_294
HMPREF1120_02776                  1.2e-10  387_[+4]_593
HMPREF1120_02904                  1.1e-09  520_[+4]_460
HMPREF1120_06974                  1.5e-09  668_[-4]_312
HMPREF1120_06129                  4.9e-09  4_[-4]_976
HMPREF1120_06148                  6.5e-09  862_[-4]_118
HMPREF1120_07017                    1e-08  512_[+4]_468
HMPREF1120_06047                  5.6e-08  718_[+4]_262
--------------------------------------------------------------------------------

--------------------------------------------------------------------------------
        Motif CCAWAKGTASSTAGGTARGY MEME-4 in BLOCKS format
--------------------------------------------------------------------------------
BL   MOTIF CCAWAKGTASSTAGGTARGY width=20 seqs=9
HMPREF1120_04947         (  676) CCAAAGGTAGGTAGGTAGGC  1
HMPREF1120_02531         (  687) CCATAAGTAGGTAGGTAGGC  1
HMPREF1120_02776         (  388) CCAAATGTACCTAGGTAAGC  1
HMPREF1120_02904         (  521) CCCTAGGTACCTAGGTAGGT  1
HMPREF1120_06974         (  669) ACAATTGTACCTAGGTAGGT  1
HMPREF1120_06129         (    5) GCAATTGTAGGTAAGTAAGT  1
HMPREF1120_06148         (  863) CAATACCTAGGTAGGTAGGT  1
HMPREF1120_07017         (  513) TGGTAGGTAGGTAGGTAGGT  1
HMPREF1120_06047         (  719) CCAACTGTAGGTATGTTACC  1
//

--------------------------------------------------------------------------------

--------------------------------------------------------------------------------
        Motif CCAWAKGTASSTAGGTARGY MEME-4 position-specific scoring matrix
--------------------------------------------------------------------------------
log-odds matrix: alength= 4 w= 20 n= 457905 bayes= 16.4827 E= 2.8e+002
  -113    137   -121   -113
  -113    159   -121   -982
   168   -121   -121   -982
   119   -982   -982     87
   146   -121   -982    -13
  -113   -121     37     87
  -982   -121    179   -982
  -982   -982   -982    204
   204   -982   -982   -982
  -982     37    137   -982
  -982     37    137   -982
  -982   -982   -982    204
   204   -982   -982   -982
  -113   -982    159   -113
  -982   -982    196   -982
  -982   -982   -982    204
   187   -982   -982   -113
    46   -982    137   -982
```

```
  -982   -121    179   -982
  -982     79   -982    119
--------------------------------------------------------------------------------

--------------------------------------------------------------------------------
        Motif CCAWAKGTASSTAGGTARGY MEME-4 position-specific probability matrix
--------------------------------------------------------------------------------
letter-probability matrix: alength= 4 w= 20 nsites= 9 E= 2.8e+002
 0.111111  0.666667  0.111111  0.111111
 0.111111  0.777778  0.111111  0.000000
 0.777778  0.111111  0.111111  0.000000
 0.555556  0.000000  0.000000  0.444444
 0.666667  0.111111  0.000000  0.222222
 0.111111  0.111111  0.333333  0.444444
 0.000000  0.111111  0.888889  0.000000
 0.000000  0.000000  0.000000  1.000000
 1.000000  0.000000  0.000000  0.000000
 0.000000  0.333333  0.666667  0.000000
 0.000000  0.333333  0.666667  0.000000
 0.000000  0.000000  0.000000  1.000000
 1.000000  0.000000  0.000000  0.000000
 0.111111  0.000000  0.777778  0.111111
 0.000000  0.000000  1.000000  0.000000
 0.000000  0.000000  0.000000  1.000000
 0.888889  0.000000  0.000000  0.111111
 0.333333  0.000000  0.666667  0.000000
 0.000000  0.111111  0.888889  0.000000
 0.000000  0.444444  0.000000  0.555556
--------------------------------------------------------------------------------

--------------------------------------------------------------------------------
        Motif CCAWAKGTASSTAGGTARGY MEME-4 regular expression
--------------------------------------------------------------------------------
CCA[AT][AT][TG]GTA[GC][GC]TAGGTA[GA]G[TC]
--------------------------------------------------------------------------------




Time 2001.74 secs.

********************************************************************************


********************************************************************************
MOTIF DRGSWHGHMGDRAKSGATGADYTGGVNSADBAMGWCSVYS MEME-5   width =  40  sites =  17  llr = 333  E-value =
1.3e+003
********************************************************************************
--------------------------------------------------------------------------------
        Motif DRGSWHGHMGDRAKSGATGADYTGGVNSADBAMGWCSVYS MEME-5 Description
--------------------------------------------------------------------------------
Simplified        A  343234155:5492:271:94:11:44:72:65:5:14::
pos.-specific     C  1:14131242:::16::22:152:1326312:5::55265
probability       G  4564118::8361446:18132169424:451:91243:4
matrix            T  32::52:3112::4:2351:2363::21:432:152:142

         bits    2.0
                 1.8
                 1.6                                  *
                 1.4             *      *    *        *
Relative         1.2       *     *   *  *    *   *    *
Entropy          1.0       *  * ** * * **    *   *   **    *
(28.2 bits)      0.8   *   *  * ** * * **   **  **  ****   *
                 0.6  **   * ** ** *** ** ****  **  **** * *
                 0.4  **** ******* ****** ***** ** ******* **
                 0.2 ************************** ********** **
                 0.0 ----------------------------------------

Multilevel           GGGCTAGAAGAGAGCGATGAACTGGAACAGGACGACCACC
```

```
consensus            AAAGAC TC GA TGTTC  GT T GCGCTTTA TGGGTG
sequence             T    T C  T         T    CG   C    T C

--------------------------------------------------------------------------------

--------------------------------------------------------------------------------
        Motif DRGSWHGHMGDRAKSGATGADYTGGVNSADBAMGWCSVYS MEME-5 sites sorted by position p-value
--------------------------------------------------------------------------------
Sequence name            Strand  Start   P-value                              Site
-------------            ------  ----- ---------            ----------------------------------------
HMPREF1120_07049             +    474  6.15e-15 CGGGAAACCC TGGATAGCCGGGAGGGATGAATTGGAACAAGAAGTCCATC TATAAGAAGA
HMPREF1120_07050             -    148  6.15e-15 CGGGAAACCC TGGATAGCCGGGAGGGATGAATTGGAACAAGAAGTCCATC TATAAGAAGA
HMPREF1120_03846             +    340  1.89e-13 AGCACCTGAA TGGGTTGAAGTGAGCGAAGAACTGGCGCCTGAAGATGCTC TGGGGATGGG
HMPREF1120_07560             -     41  1.07e-11 CACATCCCAA GAGGATGCACAGATCAATGAACTGGCCCAGTAAGTGAGTC AACCATCTCA
HMPREF1120_06441             -    405  1.21e-11 CCAGCCGGAC GAGGTCGACGAGAACGACGAACCTGCCGACGACGATGCCG ATCCAGACGC
HMPREF1120_02117             -    861  4.25e-11 AGCTGAAGGG GGAAGAGAAGAGAGCTTTGAGCTTGCGGCGTACGACCCCT CTTTAAAGTA
HMPREF1120_06770             -     16  8.91e-11 TACCGTGAGG ATGCTCACCGTGAGGGATCAGTCGGAACAGGTCGACCACG TTGACGAAAC
HMPREF1120_00038             -    426  1.97e-10 TGCCTGTGCC TGGCTAATAGTAAGCTTGGATCTGGGTCCTCAAGTTCGTC CTTGATCGGC
HMPREF1120_06902             +    551  3.16e-10 ACGGGTGGAT GGACTGGAATGGATGGATGAGGTTGAAGAGTTCGTCCTCC TCGCGAAGCA
HMPREF1120_02623             -    483  2.57e-09 AATTGATGGG AAAGTCGTCCAGGACGACGAAGAGGAAGAGGAAGACATCG CGCTCTCAAA
HMPREF1120_07664             -    452  4.01e-09 TACGGATACC AAGCTTGATGAAATCATCTATCTGCGAGAACAAGTGCGCG CCCATCAATC
HMPREF1120_05439             +    626  7.04e-09 CAAACGCCGC GTGCACGTCGGAATCGATCGTCCGCCCCATGGCGTCCGCT CCAGATCCCT
HMPREF1120_02127             -    572  8.05e-09 TCCAGGCCAC CAAGGTGAAGAAAGCGATGGACTAGGTTATTACGAGGATG GGGTCAAAAG
HMPREF1120_05435             +    785  8.05e-09 AATCGATGAC ATGCAAGTAGTGATGAAAGAGGGTGGTCCTCGCGACGGCC CCGGCACAGG
HMPREF1120_05843             -    453  1.96e-08 CAATGAAAAC GACGACCTAGAAGACGACGACCTGGGCCCCGAAGACGATC TGATTGGAGA
HMPREF1120_01106             +    279  2.21e-08 TATCGTCGCT TGACAAGATCAGACGTTTCAGTAGGGGCATTTCGTTCCCT GGGTACGGCT
HMPREF1120_05715             +     25  6.27e-08 TGTCCGGTGC AGGGCAGAAGGAATGTTGGATTTTGAGGAGCTCTGGGACG TTGCTTCTTT
--------------------------------------------------------------------------------

--------------------------------------------------------------------------------
        Motif DRGSWHGHMGDRAKSGATGADYTGGVNSADBAMGWCSVYS MEME-5 block diagrams
--------------------------------------------------------------------------------
SEQUENCE NAME            POSITION P-VALUE  MOTIF DIAGRAM
-------------            ----------------  -------------
HMPREF1120_07049                  6.2e-15  473_[+5]_487
HMPREF1120_07050                  6.2e-15  147_[-5]_813
HMPREF1120_03846                  1.9e-13  339_[+5]_621
HMPREF1120_07560                  1.1e-11  40_[-5]_920
HMPREF1120_06441                  1.2e-11  404_[-5]_556
HMPREF1120_02117                  4.2e-11  860_[-5]_100
HMPREF1120_06770                  8.9e-11  15_[-5]_945
HMPREF1120_00038                    2e-10  425_[-5]_535
HMPREF1120_06902                  3.2e-10  550_[+5]_410
HMPREF1120_02623                  2.6e-09  482_[-5]_478
HMPREF1120_07664                    4e-09  451_[-5]_509
HMPREF1120_05439                    7e-09  625_[+5]_335
HMPREF1120_02127                  8.1e-09  571_[-5]_389
HMPREF1120_05435                  8.1e-09  784_[+5]_176
HMPREF1120_05843                    2e-08  452_[-5]_508
HMPREF1120_01106                  2.2e-08  278_[+5]_682
HMPREF1120_05715                  6.3e-08  24_[+5]_936
--------------------------------------------------------------------------------
```

```
--------------------------------------------------------------------------------
	Motif DRGSWHGHMGDRAKSGATGADYTGGVNSADBAMGWCSVYS MEME-5 in BLOCKS format
--------------------------------------------------------------------------------
BL   MOTIF DRGSWHGHMGDRAKSGATGADYTGGVNSADBAMGWCSVYS width=40 seqs=17
HMPREF1120_07049         (  474) TGGATAGCCGGGAGGGATGAATTGGAACAAGAAGTCCATC  1 
HMPREF1120_07050         (  148) TGGATAGCCGGGAGGGATGAATTGGAACAAGAAGTCCATC  1 
HMPREF1120_03846         (  340) TGGGTTGAAGTGAGCGAAGAACTGGCGCCTGAAGATGCTC  1 
HMPREF1120_07560         (   41) GAGGATGCACAGATCAATGAACTGGCCCAGTAAGTGAGTC  1 
HMPREF1120_06441         (  405) GAGGTCGACGAGAACGACGAACCTGCCGACGACGATGCCG  1 
HMPREF1120_02117         (  861) GGAAGAGAAGAGAGCTTTGAGCTTGCGGCGTACGACCCCT  1 
HMPREF1120_06770         (   16) ATGCTCACCGTGAGGGATCAGTCGGAACAGGTCGACCACG  1 
HMPREF1120_00038         (  426) TGGCTAATAGTAAGCTTGGATCTGGGTCCTCAAGTTCGTC  1 
HMPREF1120_06902         (  551) GGACTGGAATGGATGGATGAGGTTGAAGAGTTCGTCCTCC  1 
HMPREF1120_02623         (  483) AAAGTCGTCCAGGACGACGAAGAGGAAGAGGAAGACATCG  1 
HMPREF1120_07664         (  452) AAGCTTGATGAAATCATCTATCTGCGAGAACAAGTGCGCG  1 
HMPREF1120_05439         (  626) GTGCACGTCGGAATCGATCGTCCGCCCCATGGCGTCCGCT  1 
HMPREF1120_02127         (  572) CAAGGTGAAGAAAGCGATGGACTAGGTTATTACGAGGATG  1 
HMPREF1120_05435         (  785) ATGCAAGTAGTGATGAAAGAGGGTGGTCCTCGCGACGGCC  1 
HMPREF1120_05843         (  453) GACGACCTAGAAGACGACGACCTGGGCCCCGAAGACGATC  1 
HMPREF1120_01106         (  279) TGACAAGATCAGACGTTTCAGTAGGGGCATTTCGTTCCCT  1 
HMPREF1120_05715         (   25) AGGGCAGAAGGAATGTTGGATTTTGAGGAGCTCTGGGACG  1 
//

--------------------------------------------------------------------------------

--------------------------------------------------------------------------------
	Motif DRGSWHGHMGDRAKSGATGADYTGGVNSADBAMGWCSVYS MEME-5 position-specific scoring matrix
--------------------------------------------------------------------------------
log-odds matrix: alength= 4 w= 40 n= 448565 bayes= 16.0525 E= 1.3e+003 
    28   -213     46     28 
    54  -1073     87    -46 
    28   -213    133  -1073 
   -46     68     68  -1073 
    28   -213   -113    112 
    76     19   -213     -4 
  -104   -213    168  -1073 
    95    -13  -1073     28 
   112     46  -1073   -104 
 -1073    -54    157   -204 
    95  -1073     19     -4 
    54  -1073    133  -1073 
   186  -1073   -113  -1073 
   -46   -213     68     54 
 -1073    119     68  -1073 
   -46  -1073    119     -4 
   154  -1073  -1073     28 
  -104    -13   -113    112 
 -1073    -54    157   -204 
   186  -1073   -113  -1073 
    76   -213     19     -4 
 -1073    104    -54     28 
  -104    -54   -213    141 
  -204  -1073    133     28 
 -1073   -113    178  -1073 
    54     19     46  -1073 
    54    -13    -13    -46 
 -1073    119     46   -204 
   154     19  -1073  -1073 
   -46   -113     46     54 
 -1073    -13     87     28 
   141  -1073   -113     -4 
    95    104  -1073  -1073 
 -1073  -1073    187   -204 
    95  -1073   -213     95 
 -1073    104    -13     -4 
  -104    104     46  -1073 
    54    -13     19   -104 
 -1073    119  -1073     76 
```

```
 -1073     87     46    -46
--------------------------------------------------------------------------------

--------------------------------------------------------------------------------
        Motif DRGSWHGHMGDRAKSGATGADYTGGVNSADBAMGWCSVYS MEME-5 position-specific probability matrix
--------------------------------------------------------------------------------
letter-probability matrix: alength= 4 w= 40 nsites= 17 E= 1.3e+003
 0.294118  0.058824  0.352941  0.294118
 0.352941  0.000000  0.470588  0.176471
 0.294118  0.058824  0.647059  0.000000
 0.176471  0.411765  0.411765  0.000000
 0.294118  0.058824  0.117647  0.529412
 0.411765  0.294118  0.058824  0.235294
 0.117647  0.058824  0.823529  0.000000
 0.470588  0.235294  0.000000  0.294118
 0.529412  0.352941  0.000000  0.117647
 0.000000  0.176471  0.764706  0.058824
 0.470588  0.000000  0.294118  0.235294
 0.352941  0.000000  0.647059  0.000000
 0.882353  0.000000  0.117647  0.000000
 0.176471  0.058824  0.411765  0.352941
 0.000000  0.588235  0.411765  0.000000
 0.176471  0.000000  0.588235  0.235294
 0.705882  0.000000  0.000000  0.294118
 0.117647  0.235294  0.117647  0.529412
 0.000000  0.176471  0.764706  0.058824
 0.882353  0.000000  0.117647  0.000000
 0.411765  0.058824  0.294118  0.235294
 0.000000  0.529412  0.176471  0.294118
 0.117647  0.176471  0.058824  0.647059
 0.058824  0.000000  0.647059  0.294118
 0.000000  0.117647  0.882353  0.000000
 0.352941  0.294118  0.352941  0.000000
 0.352941  0.235294  0.235294  0.176471
 0.000000  0.588235  0.352941  0.058824
 0.705882  0.294118  0.000000  0.000000
 0.176471  0.117647  0.352941  0.352941
 0.000000  0.235294  0.470588  0.294118
 0.647059  0.000000  0.117647  0.235294
 0.470588  0.529412  0.000000  0.000000
 0.000000  0.000000  0.941176  0.058824
 0.470588  0.000000  0.058824  0.470588
 0.000000  0.529412  0.235294  0.235294
 0.117647  0.529412  0.352941  0.000000
 0.352941  0.235294  0.294118  0.117647
 0.000000  0.588235  0.000000  0.411765
 0.000000  0.470588  0.352941  0.176471
--------------------------------------------------------------------------------

--------------------------------------------------------------------------------
        Motif DRGSWHGHMGDRAKSGATGADYTGGVNSADBAMGWCSVYS MEME-5 regular expression
--------------------------------------------------------------------------------
[GAT][GA][GA][CG][TA][ACT]G[ATC][AC]G[AGT][GA]A[GT][CG][GT][AT][TC]GA[AGT][CT]T[GT]G[AGC][ACG][CG][AC][GT]
[GTC][AT][CA]G[AT][CGT][CG][AGC][CT][CG]
--------------------------------------------------------------------------------




Time 2492.86 secs.

********************************************************************************


********************************************************************************
MOTIF TATMCCATGMMAYSWRCCCAMWKYNRASRGKRTKMCGYSRKCTBCWMTGK MEME-6 width =  50  sites =   6  llr = 238  E-
value = 6.0e+004
********************************************************************************
--------------------------------------------------------------------------------
```

```
        Motif TATMCCATGMMAYSWRCCCAMWKYNRASRGKRTKMCGYSRKCTBCWMTGK MEME-6 Description
--------------------------------------------------------------------------------
Simplified        A  27:72:8::53a2:332::873::358:3::5::3::227:::::75:2:
pos.-specific     C  :2238a22237:35::8aa:3::53::7222:::5a:55:2a:38:52::
probability       G  :::::::72:::5:7:::::3225235835272:a:333::32:::87
matrix            T  828::::82:::5:7::::2:7732:::::5:83:::3::5:a3:3:8:3

         bits    2.0      *     *     **                **    **
                 1.8      *     *     **                **    **
                 1.6      *     *     **                **    **
                 1.4 * *  ***   *     ***       *     *  **    **    *
Relative         1.2 * * ****   *  * **** *    *  *  *  **    ** ** **
Entropy          1.0 * ******  ** **********  *** * *** **  * ** ******
(57.3 bits)      0.8 ********* ** **********  *** * *** **  * ** ******
                 0.6 ************************ ****************** ******
                 0.4 ************************ *************************
                 0.2 ************************ *************************
                 0.0 --------------------------------------------------

Multilevel           TATACCATGACATCTGCCCAATTCAAACGGTATGCCGCCATCTCCAATGG
consensus               C     CA CGAA    CAGTCG GA GG TA  TGGG  G TC  T
sequence                                                        T

--------------------------------------------------------------------------------

--------------------------------------------------------------------------------
        Motif TATMCCATGMMAYSWRCCCAMWKYNRASRGKRTKMCGYSRKCTBCWMTGK MEME-6 sites sorted by position p-value
--------------------------------------------------------------------------------
Sequence name            Strand  Start   P-value                                  Site
-------------            ------  ----- ---------            -----------------------------------------------
---
HMPREF1120_07049             -    399  1.40e-24 TGAAGCTTGG
TATACCATGACATGTGCCCAATGTCGAGGGTATGCCGTCAGCTTCAATGG TAAGGGAGAG
HMPREF1120_07050             +    213  1.40e-24 TGAAGCTTGG
TATACCATGACATGTGCCCAATGTCGAGGGTATGCCGTCAGCTTCAATGG TAAGGGAGAG
HMPREF1120_02510             +    936  1.48e-20 CCTCTTTGAA
TATACCATGCAACCAACCCAAATCAAACAGGGTGACGCGGTCTCCTCTGT GGATCCATTA
HMPREF1120_02509             -    686  1.48e-20 CCTCTTTGAA
TATACCATGCAACCAACCCAAATCAAACAGGGTGACGCGGTCTCCTCTGT GGATCCATTA
HMPREF1120_06441             -    193  1.31e-15 GAACTGCGCG
ACTCACCCTACATCTGCCCTCTTCTGACGGCATTCCGACATCTGGACCAG GTTGTGGCGG
HMPREF1120_06490             -    426  1.93e-15 GAAGATACAA
TTCCCCATCGCAAGTGACCACTTGGAGCCCTGGTGCGCAACCTGCAATGG CTGAATCCTA
--------------------------------------------------------------------------------

--------------------------------------------------------------------------------
        Motif TATMCCATGMMAYSWRCCCAMWKYNRASRGKRTKMCGYSRKCTBCWMTGK MEME-6 block diagrams
--------------------------------------------------------------------------------
SEQUENCE NAME            POSITION P-VALUE  MOTIF DIAGRAM
-------------            ----------------  -------------
HMPREF1120_07049                  1.4e-24  398_[-6]_552
HMPREF1120_07050                  1.4e-24  212_[+6]_738
HMPREF1120_02510                  1.5e-20  935_[+6]_15
HMPREF1120_02509                  1.5e-20  685_[-6]_265
HMPREF1120_06441                  1.3e-15  192_[-6]_758
HMPREF1120_06490                  1.9e-15  425_[-6]_525
--------------------------------------------------------------------------------

--------------------------------------------------------------------------------
        Motif TATMCCATGMMAYSWRCCCAMWKYNRASRGKRTKMCGYSRKCTBCWMTGK MEME-6 in BLOCKS format
--------------------------------------------------------------------------------
BL   MOTIF TATMCCATGMMAYSWRCCCAMWKYNRASRGKRTKMCGYSRKCTBCWMTGK width=50 seqs=6
HMPREF1120_07049         (  399) TATACCATGACATGTGCCCAATGTCGAGGGTATGCCGTCAGCTTCAATGG  1
HMPREF1120_07050         (  213) TATACCATGACATGTGCCCAATGTCGAGGGTATGCCGTCAGCTTCAATGG  1
HMPREF1120_02510         (  936) TATACCATGCAACCAACCCAAATCAAACAGGGTGACGCGGTCTCCTCTGT  1
HMPREF1120_02509         (  686) TATACCATGCAACCAACCCAAATCAAACAGGGTGACGCGGTCTCCTCTGT  1
HMPREF1120_06441         (  193) ACTCACCCTACATCTGCCCTCTTCTGACGGCATTCCGACATCTGGACCAG  1
HMPREF1120_06490         (  426) TTCCCCATCGCAAGTGACCACTTGGAGCCCTGGTGCGCAACCTGCAATGG  1
//
```

```
--------------------------------------------------------------------------------

--------------------------------------------------------------------------------
	Motif TATMCCATGMMAYSWRCCCAMWKYNRASRGKRTKMCGYSRKCTBCWMTGK MEME-6 position-specific scoring matrix
--------------------------------------------------------------------------------
log-odds matrix: alength= 4 w= 50 n= 443895 bayes= 15.8338 E= 6.0e+004 
   -54   -923   -923    178 
   146    -63   -923    -54 
  -923    -63   -923    178 
   146     37   -923   -923 
   -54    169   -923   -923 
  -923    196   -923   -923 
   178    -63   -923   -923 
  -923    -63   -923    178 
  -923    -63    137    -54 
   104     37    -63   -923 
    46    137   -923   -923 
   204   -923   -923   -923 
   -54     37   -923    104 
  -923     96     96   -923 
    46   -923   -923    146 
    46   -923    137   -923 
   -54    169   -923   -923 
  -923    196   -923   -923 
  -923    196   -923   -923 
   178   -923   -923    -54 
   146     37   -923   -923 
    46   -923   -923    146 
  -923   -923     37    146 
  -923     96    -63     46 
    46     37    -63    -54 
   104   -923     96   -923 
   178   -923    -63   -923 
  -923    137     37   -923 
    46    -63     96   -923 
  -923    -63    169   -923 
  -923    -63     37    104 
   104   -923     96   -923 
  -923   -923    -63    178 
  -923   -923    137     46 
    46     96    -63   -923 
  -923    196   -923   -923 
  -923   -923    196   -923 
   -54     96   -923     46 
   -54     96     37   -923 
   146   -923     37   -923 
  -923    -63     37    104 
  -923    196   -923   -923 
  -923   -923   -923    204 
  -923     37     37     46 
  -923    169    -63   -923 
   146   -923   -923     46 
   104     96   -923   -923 
  -923    -63   -923    178 
   -54   -923    169   -923 
  -923   -923    137     46 
--------------------------------------------------------------------------------

--------------------------------------------------------------------------------
	Motif TATMCCATGMMAYSWRCCCAMWKYNRASRGKRTKMCGYSRKCTBCWMTGK MEME-6 position-specific probability
matrix
--------------------------------------------------------------------------------
letter-probability matrix: alength= 4 w= 50 nsites= 6 E= 6.0e+004 
  0.166667  0.000000  0.000000  0.833333 
  0.666667  0.166667  0.000000  0.166667 
  0.000000  0.166667  0.000000  0.833333 
  0.666667  0.333333  0.000000  0.000000 
  0.166667  0.833333  0.000000  0.000000 
```

```
 0.000000  1.000000  0.000000  0.000000
 0.833333  0.166667  0.000000  0.000000
 0.000000  0.166667  0.000000  0.833333
 0.000000  0.166667  0.666667  0.166667
 0.500000  0.333333  0.166667  0.000000
 0.333333  0.666667  0.000000  0.000000
 1.000000  0.000000  0.000000  0.000000
 0.166667  0.333333  0.000000  0.500000
 0.000000  0.500000  0.500000  0.000000
 0.333333  0.000000  0.000000  0.666667
 0.333333  0.000000  0.666667  0.000000
 0.166667  0.833333  0.000000  0.000000
 0.000000  1.000000  0.000000  0.000000
 0.000000  1.000000  0.000000  0.000000
 0.833333  0.000000  0.000000  0.166667
 0.666667  0.333333  0.000000  0.000000
 0.333333  0.000000  0.000000  0.666667
 0.000000  0.000000  0.333333  0.666667
 0.000000  0.500000  0.166667  0.333333
 0.333333  0.333333  0.166667  0.166667
 0.500000  0.000000  0.500000  0.000000
 0.833333  0.000000  0.166667  0.000000
 0.000000  0.666667  0.333333  0.000000
 0.333333  0.166667  0.500000  0.000000
 0.000000  0.166667  0.833333  0.000000
 0.000000  0.166667  0.333333  0.500000
 0.500000  0.000000  0.500000  0.000000
 0.000000  0.000000  0.166667  0.833333
 0.000000  0.000000  0.666667  0.333333
 0.333333  0.500000  0.166667  0.000000
 0.000000  1.000000  0.000000  0.000000
 0.000000  0.000000  1.000000  0.000000
 0.166667  0.500000  0.000000  0.333333
 0.166667  0.500000  0.333333  0.000000
 0.666667  0.000000  0.333333  0.000000
 0.000000  0.166667  0.333333  0.500000
 0.000000  1.000000  0.000000  0.000000
 0.000000  0.000000  0.000000  1.000000
 0.000000  0.333333  0.333333  0.333333
 0.000000  0.833333  0.166667  0.000000
 0.666667  0.000000  0.000000  0.333333
 0.500000  0.500000  0.000000  0.000000
 0.000000  0.166667  0.000000  0.833333
 0.166667  0.000000  0.833333  0.000000
 0.000000  0.000000  0.666667  0.333333
--------------------------------------------------------------------------------

--------------------------------------------------------------------------------
        Motif TATMCCATGMMAYSWRCCCAMWKYNRASRGKRTKMCGYSRKCTBCWMTGK MEME-6 regular expression
--------------------------------------------------------------------------------
TAT[AC]CCATG[AC][CA]A[TC][CG][TA][GA]CCCA[AC][TA][TG][CT][AC][AG]A[CG][GA]G[TG][AG]T[GT][CA]CG[CT][CG][AG]
[TG]CT[CGT]C[AT][AC]TG[GT]
--------------------------------------------------------------------------------




Time 2983.61 secs.

********************************************************************************


********************************************************************************
MOTIF CDCTTGCTBTCRATNTYGKMYTCTTTSAWCWKKTTYYVAAMYTSRACTTC MEME-7 width =  50  sites =   7  llr = 245  E-
value = 5.9e-001
********************************************************************************
--------------------------------------------------------------------------------
        Motif CDCTTGCTBTCRATNTYGKMYTCTTTSAWCWKKTTYYVAAMYTSRACTTC MEME-7 Description
--------------------------------------------------------------------------------
```

```
Simplified        A  33::1:::::69:3::114:::3:::73:3::1:1:46741:149::::
pos.-specific     C  7:7:117:4:9::13:6::4437::143:9:1:1:3631344361:a3:7
probability       G  :431:7::3113111::96:::3:::6:1:146:3::31:11:331::1:
matrix            T  :3:9713a39:1:73a4:3167:7a9::616447764:1::37:1::793

         bits    2.0        *       *        *                     *
                 1.8        *       *        *                     *
                 1.6        *       *        *                     *
                 1.4    *   * ** *  * *      **   *               ** *
Relative         1.2    *   * ** *  * *   * *** * *    *    *  *  ****
Entropy          1.0 * **  ** ** *  ***  ******** *  * * *  *  *  *****
(50.5 bits)      0.8 * ****** ** ** ***  ******** *  *** *  *  *  *****
                 0.6 * ****** ***** **********************  ** ** *****
                 0.4 ************** ************************** ** *****
                 0.2 ************** ***********************************
                 0.0 --------------------------------------------------

Multilevel           CGCTTGCTCTCAATATCGGATTCTTTGATCTGGTTTCAAAACTCAACTTC
consensus            AAG   T G  G  C T TCCCGA  CCA ATT GCTC CCTCGG  C T
sequence              T      T     T                      G

--------------------------------------------------------------------------------

--------------------------------------------------------------------------------
        Motif CDCTTGCTBTCRATNTYGKMYTCTTTSAWCWKKTTYYVAAMYTSRACTTC MEME-7 sites sorted by position p-value
--------------------------------------------------------------------------------
Sequence name            Strand  Start   P-value                                  Site
-------------            ------  ----- ---------            -----------------------------------------------
---
HMPREF1120_07049             -    327  5.12e-25 AAAGACTCTT
CGCTTGCTCTCAATTTCGGACTGTTTGATCAGGTTTCAAAACTCGACTTC CGACGAGGTT
HMPREF1120_07050             +    285  5.12e-25 AAAGACTCTT
CGCTTGCTCTCAATTTCGGACTGTTTGATCAGGTTTCAAAACTCGACTTC CGACGAGGTT
HMPREF1120_07397             +    288  3.85e-17 ATGATGCTCC
AAGTTGTTGTCAATGTTGACTTCTTTCCACTTTTGCCAGACTTCAACCTC CCTTCTTCGA
HMPREF1120_09019             +     14  4.52e-15 CTGCTCAGCG
AACTTGCTTGCGAGATCGTCTCCTTTGAGCTGGATATGTAGATGAACTTT CGCCAATGCG
HMPREF1120_04574             +    879  7.94e-15 ACCTGATTTT
CTGGTCCTGTCAACATTGGATCCATTCATTTTGTTTCCACCTCCCGCTGC ACGGGCAAAA
HMPREF1120_09134             +    920  8.85e-15 TTCTTCCAGT
CGCTCGTTTTGGATCTTGGTCTCTTTGCTCGTTCGTTGACCGTGTACCTT GACAACTAAT
HMPREF1120_01817             +    934  1.35e-14 CCAGACTCAA
CTCTATCTCTCTGTCTCATCTTCATCCAACTCTTTCTCCAACCAAACTTC ATACCAACTC
--------------------------------------------------------------------------------

--------------------------------------------------------------------------------
        Motif CDCTTGCTBTCRATNTYGKMYTCTTTSAWCWKKTTYYVAAMYTSRACTTC MEME-7 block diagrams
--------------------------------------------------------------------------------
SEQUENCE NAME            POSITION P-VALUE  MOTIF DIAGRAM
-------------            ----------------  -------------
HMPREF1120_07049                  5.1e-25  326_[-7]_624
HMPREF1120_07050                  5.1e-25  284_[+7]_666
HMPREF1120_07397                  3.8e-17  287_[+7]_663
HMPREF1120_09019                  4.5e-15  13_[+7]_937
HMPREF1120_04574                  7.9e-15  878_[+7]_72
HMPREF1120_09134                  8.9e-15  919_[+7]_31
HMPREF1120_01817                  1.4e-14  933_[+7]_17
--------------------------------------------------------------------------------

--------------------------------------------------------------------------------
        Motif CDCTTGCTBTCRATNTYGKMYTCTTTSAWCWKKTTYYVAAMYTSRACTTC MEME-7 in BLOCKS format
--------------------------------------------------------------------------------
BL   MOTIF CDCTTGCTBTCRATNTYGKMYTCTTTSAWCWKKTTYYVAAMYTSRACTTC width=50 seqs=7
HMPREF1120_07049         (  327) CGCTTGCTCTCAATTTCGGACTGTTTGATCAGGTTTCAAAACTCGACTTC  1
HMPREF1120_07050         (  285) CGCTTGCTCTCAATTTCGGACTGTTTGATCAGGTTTCAAAACTCGACTTC  1
HMPREF1120_07397         (  288) AAGTTGTTGTCAATGTTGACTTCTTTCCACTTTTGCCAGACTTCAACCTC  1
HMPREF1120_09019         (   14) AACTTGCTTGCGAGATCGTCTCCTTTGAGCTGGATATGTAGATGAACTTT  1
HMPREF1120_04574         (  879) CTGGTCCTGTCAACATTGGATCCATTCATTTTGTTTCCACCTCCCGCTGC  1
HMPREF1120_09134         (  920) CGCTCGTTTTGGATCTTGGTCTCTTTGCTCGTTCGTTGACCGTGTACCTT  1
```

```
HMPREF1120_01817         (  934) CTCTATCTCTCTGTCTCATCTTCATCCAACTCTTTCTCCAACCAAACTTC  1
//

--------------------------------------------------------------------------------

--------------------------------------------------------------------------------
        Motif CDCTTGCTBTCRATNTYGKMYTCTTTSAWCWKKTTYYVAAMYTSRACTTC MEME-7 position-specific scoring matrix
--------------------------------------------------------------------------------
log-odds matrix: alength= 4 w= 50 n= 443895 bayes= 15.7964 E= 5.9e-001
    24    147   -945   -945
    24   -945     74     24
  -945    147     15   -945
  -945   -945    -85    182
   -76    -85   -945    156
  -945    -85    147    -76
  -945    147   -945     24
  -945   -945   -945    204
  -945     74     15     24
  -945   -945    -85    182
  -945    173    -85   -945
   123   -945     15    -76
   182   -945    -85   -945
  -945    -85    -85    156
    24     15    -85     24
  -945   -945   -945    204
  -945    115   -945     82
   -76   -945    173   -945
   -76   -945    115     24
    82     74   -945    -76
  -945     74   -945    123
  -945     15   -945    156
  -945    147     15   -945
    24   -945   -945    156
  -945   -945   -945    204
  -945    -85   -945    182
  -945     74    115   -945
   156     15   -945   -945
    24   -945    -85    123
  -945    173   -945    -76
    24   -945    -85    123
  -945    -85     74     82
  -945   -945    115     82
   -76    -85   -945    156
  -945   -945     15    156
   -76     15   -945    123
  -945    115   -945     82
    82     15     15   -945
   123    -85    -85    -76
   156     15   -945   -945
    82     74    -85   -945
   -76     74    -85     24
  -945     15   -945    156
   -76    115     15   -945
    82    -85     15    -76
   182   -945    -85   -945
  -945    196   -945   -945
  -945     15   -945    156
  -945   -945    -85    182
  -945    147   -945     24
--------------------------------------------------------------------------------

--------------------------------------------------------------------------------
        Motif CDCTTGCTBTCRATNTYGKMYTCTTTSAWCWKKTTYYVAAMYTSRACTTC MEME-7 position-specific probability
matrix
--------------------------------------------------------------------------------
letter-probability matrix: alength= 4 w= 50 nsites= 7 E= 5.9e-001
 0.285714  0.714286  0.000000  0.000000
 0.285714  0.000000  0.428571  0.285714
 0.000000  0.714286  0.285714  0.000000
```

```
 0.000000  0.000000  0.142857  0.857143
 0.142857  0.142857  0.000000  0.714286
 0.000000  0.142857  0.714286  0.142857
 0.000000  0.714286  0.000000  0.285714
 0.000000  0.000000  0.000000  1.000000
 0.000000  0.428571  0.285714  0.285714
 0.000000  0.000000  0.142857  0.857143
 0.000000  0.857143  0.142857  0.000000
 0.571429  0.000000  0.285714  0.142857
 0.857143  0.000000  0.142857  0.000000
 0.000000  0.142857  0.142857  0.714286
 0.285714  0.285714  0.142857  0.285714
 0.000000  0.000000  0.000000  1.000000
 0.000000  0.571429  0.000000  0.428571
 0.142857  0.000000  0.857143  0.000000
 0.142857  0.000000  0.571429  0.285714
 0.428571  0.428571  0.000000  0.142857
 0.000000  0.428571  0.000000  0.571429
 0.000000  0.285714  0.000000  0.714286
 0.000000  0.714286  0.285714  0.000000
 0.285714  0.000000  0.000000  0.714286
 0.000000  0.000000  0.000000  1.000000
 0.000000  0.142857  0.000000  0.857143
 0.000000  0.428571  0.571429  0.000000
 0.714286  0.285714  0.000000  0.000000
 0.285714  0.000000  0.142857  0.571429
 0.000000  0.857143  0.000000  0.142857
 0.285714  0.000000  0.142857  0.571429
 0.000000  0.142857  0.428571  0.428571
 0.000000  0.000000  0.571429  0.428571
 0.142857  0.142857  0.000000  0.714286
 0.000000  0.000000  0.285714  0.714286
 0.142857  0.285714  0.000000  0.571429
 0.000000  0.571429  0.000000  0.428571
 0.428571  0.285714  0.285714  0.000000
 0.571429  0.142857  0.142857  0.142857
 0.714286  0.285714  0.000000  0.000000
 0.428571  0.428571  0.142857  0.000000
 0.142857  0.428571  0.142857  0.285714
 0.000000  0.285714  0.000000  0.714286
 0.142857  0.571429  0.285714  0.000000
 0.428571  0.142857  0.285714  0.142857
 0.857143  0.000000  0.142857  0.000000
 0.000000  1.000000  0.000000  0.000000
 0.000000  0.285714  0.000000  0.714286
 0.000000  0.000000  0.142857  0.857143
 0.000000  0.714286  0.000000  0.285714
--------------------------------------------------------------------------------

--------------------------------------------------------------------------------
	Motif CDCTTGCTBTCRATNTYGKMYTCTTTSAWCWKKTTYYVAAMYTSRACTTC MEME-7 regular expression
--------------------------------------------------------------------------------
[CA][GAT][CG]TTG[CT]T[CGT]TC[AG]AT[ACT]T[CT]G[GT][AC][TC][TC][CG][TA]TT[GC][AC][TA]C[TA][GT][GT]T[TG][TC]
[CT][ACG]A[AC][AC][CT][TC][CG][AG]AC[TC]T[CT]
--------------------------------------------------------------------------------




Time 3474.58 secs.

********************************************************************************


********************************************************************************
MOTIF WGCTWCKYMKSCCACRASKTCGARRCCSKTGCGCTTGAAMT MEME-8	width =  41  sites =   3  llr = 141  E-value = 
2.1e+005
********************************************************************************
--------------------------------------------------------------------------------
```

```
        Motif WGCTWCKYMKSCCACRASKTCGARRCCSKTGCGCTTGAAMT MEME-8 Description
--------------------------------------------------------------------------------
Simplified        A  3:::7:::3::::a:7a:::::a33:::::::::::aa7:
pos.-specific     C  ::a::a:77:7aa:a::3::a::::aa3:::a:a:::::3:
probability       G  :a::::3::33::::3:73::a:77::77:a:a:::a::::
matrix            T  7::a3:73:7:::::::7a::::::::3a::::aa::::a

         bits    2.0  *** *     **** *  ****  **  ********** *
                 1.8  *** *     **** *  ****  **  ********** *
                 1.6  *** *     **** *  ****  **  ********** *
                 1.4  *** *     **** *  ****  **  ********** *
Relative         1.2 ******     **** *  ****  **  ********** *
Entropy          1.0 *****************************************
(68.0 bits)      0.8 *****************************************
                 0.6 *****************************************
                 0.4 *****************************************
                 0.2 *****************************************
                 0.0 -----------------------------------------

Multilevel           TGCTACTCCTCCCACAAGTTCGAGGCCGGTGCGCTTGAAAT
consensus            A   T GTAGG    G CG    AA  CT          C
sequence

--------------------------------------------------------------------------------

--------------------------------------------------------------------------------
        Motif WGCTWCKYMKSCCACRASKTCGARRCCSKTGCGCTTGAAMT MEME-8 sites sorted by position p-value
--------------------------------------------------------------------------------
Sequence name            Strand  Start   P-value                              Site
-------------            ------  ----- ---------            -----------------------------------------
HMPREF1120_07049             -    567  2.34e-25 TGTTGGCGGC TGCTACTCCTCCCACAAGTTCGAGGCCGGTGCGCTTGAAAT
ATTAGCATCC
HMPREF1120_07050             +     54  2.34e-25 TGTTGGCGGC TGCTACTCCTCCCACAAGTTCGAGGCCGGTGCGCTTGAAAT
ATTAGCATCC
HMPREF1120_06238             +    427  4.08e-20 CACTGTCGCG AGCTTCGTAGGCCACGACGTCGAAACCCTTGCGCTTGAACT
GCTCGATGGC
--------------------------------------------------------------------------------

--------------------------------------------------------------------------------
        Motif WGCTWCKYMKSCCACRASKTCGARRCCSKTGCGCTTGAAMT MEME-8 block diagrams
--------------------------------------------------------------------------------
SEQUENCE NAME            POSITION P-VALUE  MOTIF DIAGRAM
-------------            ----------------  -------------
HMPREF1120_07049                  2.3e-25  566_[-8]_393
HMPREF1120_07050                  2.3e-25  53_[+8]_906
HMPREF1120_06238                  4.1e-20  426_[+8]_533
--------------------------------------------------------------------------------

--------------------------------------------------------------------------------
        Motif WGCTWCKYMKSCCACRASKTCGARRCCSKTGCGCTTGAAMT MEME-8 in BLOCKS format
--------------------------------------------------------------------------------
BL   MOTIF WGCTWCKYMKSCCACRASKTCGARRCCSKTGCGCTTGAAMT width=41 seqs=3
HMPREF1120_07049         (  567) TGCTACTCCTCCCACAAGTTCGAGGCCGGTGCGCTTGAAAT  1
HMPREF1120_07050         (   54) TGCTACTCCTCCCACAAGTTCGAGGCCGGTGCGCTTGAAAT  1
HMPREF1120_06238         (  427) AGCTTCGTAGGCCACGACGTCGAAACCCTTGCGCTTGAACT  1
//

--------------------------------------------------------------------------------

--------------------------------------------------------------------------------
        Motif WGCTWCKYMKSCCACRASKTCGARRCCSKTGCGCTTGAAMT MEME-8 position-specific scoring matrix
--------------------------------------------------------------------------------
log-odds matrix: alength= 4 w= 41 n= 448098 bayes= 15.9661 E= 2.1e+005
    46   -823   -823    145
  -823   -823    195   -823
  -823    195   -823   -823
  -823   -823   -823    204
   145   -823   -823     46
  -823    195   -823   -823
```

```
   -823   -823     37    145
   -823    137   -823     46
     46    137   -823   -823
   -823   -823     37    145
   -823    137     37   -823
   -823    195   -823   -823
   -823    195   -823   -823
    204   -823   -823   -823
   -823    195   -823   -823
    145   -823     37   -823
    204   -823   -823   -823
   -823     37    137   -823
   -823   -823     37    145
   -823   -823   -823    204
   -823    195   -823   -823
   -823   -823    195   -823
    204   -823   -823   -823
     46   -823    137   -823
     46   -823    137   -823
   -823    195   -823   -823
   -823    195   -823   -823
   -823     37    137   -823
   -823   -823    137     46
   -823   -823   -823    204
   -823   -823    195   -823
   -823    195   -823   -823
   -823   -823    195   -823
   -823    195   -823   -823
   -823   -823   -823    204
   -823   -823   -823    204
   -823   -823    195   -823
    204   -823   -823   -823
    204   -823   -823   -823
    145     37   -823   -823
   -823   -823   -823    204
--------------------------------------------------------------------------------

--------------------------------------------------------------------------------
        Motif WGCTWCKYMKSCCACRASKTCGARRCCSKTGCGCTTGAAMT MEME-8 position-specific probability matrix
--------------------------------------------------------------------------------
letter-probability matrix: alength= 4 w= 41 nsites= 3 E= 2.1e+005
 0.333333  0.000000  0.000000  0.666667
 0.000000  0.000000  1.000000  0.000000
 0.000000  1.000000  0.000000  0.000000
 0.000000  0.000000  0.000000  1.000000
 0.666667  0.000000  0.000000  0.333333
 0.000000  1.000000  0.000000  0.000000
 0.000000  0.000000  0.333333  0.666667
 0.000000  0.666667  0.000000  0.333333
 0.333333  0.666667  0.000000  0.000000
 0.000000  0.000000  0.333333  0.666667
 0.000000  0.666667  0.333333  0.000000
 0.000000  1.000000  0.000000  0.000000
 0.000000  1.000000  0.000000  0.000000
 1.000000  0.000000  0.000000  0.000000
 0.000000  1.000000  0.000000  0.000000
 0.666667  0.000000  0.333333  0.000000
 1.000000  0.000000  0.000000  0.000000
 0.000000  0.333333  0.666667  0.000000
 0.000000  0.000000  0.333333  0.666667
 0.000000  0.000000  0.000000  1.000000
 0.000000  1.000000  0.000000  0.000000
 0.000000  0.000000  1.000000  0.000000
 1.000000  0.000000  0.000000  0.000000
 0.333333  0.000000  0.666667  0.000000
 0.333333  0.000000  0.666667  0.000000
 0.000000  1.000000  0.000000  0.000000
 0.000000  1.000000  0.000000  0.000000
 0.000000  0.333333  0.666667  0.000000
```

```
 0.000000  0.000000  0.666667  0.333333
 0.000000  0.000000  0.000000  1.000000
 0.000000  0.000000  1.000000  0.000000
 0.000000  1.000000  0.000000  0.000000
 0.000000  0.000000  1.000000  0.000000
 0.000000  1.000000  0.000000  0.000000
 0.000000  0.000000  0.000000  1.000000
 0.000000  0.000000  0.000000  1.000000
 0.000000  0.000000  1.000000  0.000000
 1.000000  0.000000  0.000000  0.000000
 1.000000  0.000000  0.000000  0.000000
 0.666667  0.333333  0.000000  0.000000
 0.000000  0.000000  0.000000  1.000000
--------------------------------------------------------------------------------

--------------------------------------------------------------------------------
        Motif WGCTWCKYMKSCCACRASKTCGARRCCSKTGCGCTTGAAMT MEME-8 regular expression
--------------------------------------------------------------------------------
[TA]GCT[AT]C[TG][CT][CA][TG][CG]CCAC[AG]A[GC][TG]TCGA[GA][GA]CC[GC][GT]TGCGCTTGAA[AC]T
--------------------------------------------------------------------------------




Time 3961.07 secs.

********************************************************************************


********************************************************************************
MOTIF GTACAATGTWSTACAWYGTW MEME-9       width =  20  sites =   8  llr = 158  E-value = 2.5e+002
********************************************************************************
--------------------------------------------------------------------------------
        Motif GTACAATGTWSTACAWYGTW MEME-9 Description
--------------------------------------------------------------------------------
Simplified        A  ::a:aa:::6:19:851::6
pos.-specific     C  :::a::1:::5:19:14:1:
probability       G  9::::::a::5::13:183:
matrix            T  1a::::9:a4:9:::44364

         bits    2.0  ***** **
                 1.8  ***** **
                 1.6  ***** **
                 1.4 *********  ***
Relative         1.2 *********  ****  *
Entropy          1.0 ***************  * *
(28.5 bits)      0.8 ***************  * *
                 0.6 **************** ***
                 0.4 **************** ***
                 0.2 ********************
                 0.0 --------------------

Multilevel           GTACAATGTACTACAACGTA
consensus                     TG   GTTTGT
sequence

--------------------------------------------------------------------------------

--------------------------------------------------------------------------------
        Motif GTACAATGTWSTACAWYGTW MEME-9 sites sorted by position p-value
--------------------------------------------------------------------------------
Sequence name            Strand  Start   P-value                    Site
-------------            ------  ----- ---------            --------------------
HMPREF1120_00038             +    317  1.44e-12 ACTAGATACT GTACAATGTACTACAATGTA CTAGTCATCG
HMPREF1120_00264             +    118  1.78e-11 CGGCTCAGTT GTACAATGTTGTACATCGTA GAGCAGGAGC
HMPREF1120_05730             +    923  1.21e-10 GTGCTCCACC GTACAATGTTGTACAATGGT ATCCCCCAAC
HMPREF1120_06864             +    604  4.94e-10 GCGGTAGTAT GTACAATGTTGTACAACTGT GGTGGCAGTC
HMPREF1120_08770             +    870  5.77e-10 TACAATGGTT GTACAATGTAGTACAAGGCT GAGAGATCCG
HMPREF1120_03399             -    799  3.26e-09 TCATCTCCGG GTACAACGTACAACGTTGTA TACAACGTGC
```

```
HMPREF1120_02776             -    776  3.42e-09 CCCTGGCACT GTACAATGTACTCGGTCGTA GTAAGATAAG
HMPREF1120_07908             -    761  3.91e-09 TTCAACGGCT TTACAATGTACTACACATTA TGCACACTCG
--------------------------------------------------------------------------------

--------------------------------------------------------------------------------
        Motif GTACAATGTWSTACAWYGTW MEME-9 block diagrams
--------------------------------------------------------------------------------
SEQUENCE NAME            POSITION P-VALUE  MOTIF DIAGRAM
-------------            ----------------  -------------
HMPREF1120_00038                  1.4e-12  316_[+9]_664
HMPREF1120_00264                  1.8e-11  117_[+9]_863
HMPREF1120_05730                  1.2e-10  922_[+9]_58
HMPREF1120_06864                  4.9e-10  603_[+9]_377
HMPREF1120_08770                  5.8e-10  869_[+9]_111
HMPREF1120_03399                  3.3e-09  798_[-9]_182
HMPREF1120_02776                  3.4e-09  775_[-9]_205
HMPREF1120_07908                  3.9e-09  760_[-9]_220
--------------------------------------------------------------------------------

--------------------------------------------------------------------------------
        Motif GTACAATGTWSTACAWYGTW MEME-9 in BLOCKS format
--------------------------------------------------------------------------------
BL   MOTIF GTACAATGTWSTACAWYGTW width=20 seqs=8
HMPREF1120_00038         (  317) GTACAATGTACTACAATGTA  1
HMPREF1120_00264         (  118) GTACAATGTTGTACATCGTA  1
HMPREF1120_05730         (  923) GTACAATGTTGTACAATGGT  1
HMPREF1120_06864         (  604) GTACAATGTTGTACAACTGT  1
HMPREF1120_08770         (  870) GTACAATGTAGTACAAGGCT  1
HMPREF1120_03399         (  799) GTACAACGTACAACGTTGTA  1
HMPREF1120_02776         (  776) GTACAATGTACTCGGTCGTA  1
HMPREF1120_07908         (  761) TTACAATGTACTACACATTA  1
//

--------------------------------------------------------------------------------

--------------------------------------------------------------------------------
        Motif GTACAATGTWSTACAWYGTW MEME-9 position-specific scoring matrix
--------------------------------------------------------------------------------
log-odds matrix: alength= 4 w= 20 n= 457905 bayes= 16.5416 E= 2.5e+002
  -965   -965    176    -96
  -965   -965   -965    204
   204   -965   -965   -965
  -965    196   -965   -965
   204   -965   -965   -965
   204   -965   -965   -965
  -965   -104   -965    185
  -965   -965    196   -965
  -965   -965   -965    204
   136   -965   -965     63
  -965     96     96   -965
   -96   -965   -965    185
   185   -104   -965   -965
  -965    176   -104   -965
   163   -965     -4   -965
   104   -104   -965     63
   -96     54   -104     63
  -965   -965    154      4
  -965   -104     -4    136
   136   -965   -965     63
--------------------------------------------------------------------------------

--------------------------------------------------------------------------------
        Motif GTACAATGTWSTACAWYGTW MEME-9 position-specific probability matrix
--------------------------------------------------------------------------------
letter-probability matrix: alength= 4 w= 20 nsites= 8 E= 2.5e+002
 0.000000  0.000000  0.875000  0.125000
 0.000000  0.000000  0.000000  1.000000
 1.000000  0.000000  0.000000  0.000000
 0.000000  1.000000  0.000000  0.000000
```

```
 1.000000  0.000000  0.000000  0.000000
 1.000000  0.000000  0.000000  0.000000
 0.000000  0.125000  0.000000  0.875000
 0.000000  0.000000  1.000000  0.000000
 0.000000  0.000000  0.000000  1.000000
 0.625000  0.000000  0.000000  0.375000
 0.000000  0.500000  0.500000  0.000000
 0.125000  0.000000  0.000000  0.875000
 0.875000  0.125000  0.000000  0.000000
 0.000000  0.875000  0.125000  0.000000
 0.750000  0.000000  0.250000  0.000000
 0.500000  0.125000  0.000000  0.375000
 0.125000  0.375000  0.125000  0.375000
 0.000000  0.000000  0.750000  0.250000
 0.000000  0.125000  0.250000  0.625000
 0.625000  0.000000  0.000000  0.375000
--------------------------------------------------------------------------------

--------------------------------------------------------------------------------
        Motif GTACAATGTWSTACAWYGTW MEME-9 regular expression
--------------------------------------------------------------------------------
GTACAATGT[AT][CG]TAC[AG][AT][CT][GT][TG][AT]
--------------------------------------------------------------------------------




Time 4445.84 secs.

********************************************************************************


********************************************************************************
MOTIF GTKATGGTCWTGGYCSADMAAGCGCCDGTGBTDCBYGCY MEME-10   width =  39  sites =   5  llr = 175  E-value =
7.8e+003
********************************************************************************
--------------------------------------------------------------------------------
        Motif GTKATGGTCWTGGYCSADMAAGCGCCDGTGBTDCBYGCY MEME-10 Description
--------------------------------------------------------------------------------
Simplified        A  :::8::22:6:2::::84688:::::4::::22:::2::
pos.-specific     C  :2:2:::2a::::684::4:::8:aa:2::4::646:a4
probability       G  a:6::a8::::8a::6:2:2:82a::482a4:424:8::
matrix            T  :84:a::6:4a::42:24::22::::2:8:284224::6

         bits    2.0 *   **  * * *          ***   *       *
                 1.8 *   **  * * *          ***   *       *
                 1.6 *   **  * * *          ***   *       *
                 1.4 *   **  * * *          ***   *       *
Relative         1.2 ** **** * *** * *  ******* *** *    **
Entropy          1.0 ******* ********* ******** *** *   ****
(50.5 bits)      0.8 ******* ********* ******** *** *   ****
                 0.6 ***************** ******** *** * * ****
                 0.4 ***************************************
                 0.2 ***************************************
                 0.0 ---------------------------------------

Multilevel           GTGATGGTCATGGCCGAAAAAGCGCCAGTGCTGCCCGCT
consensus             CTC  AA T A TTCTTCGTTG   GCG GATGGTA C
sequence                    C         G        T   T ATT

--------------------------------------------------------------------------------

--------------------------------------------------------------------------------
        Motif GTKATGGTCWTGGYCSADMAAGCGCCDGTGBTDCBYGCY MEME-10 sites sorted by position p-value
--------------------------------------------------------------------------------
Sequence name            Strand  Start   P-value                            Site
-------------            ------  ----- ---------            ---------------------------------------
HMPREF1120_07049             -    281  9.74e-21 TTCCGACGAG GTTATGGTCATGGTCGATCAAGCGCCAGTGCTGCGCGCT
TTGGGTGTCT
```

```
HMPREF1120_07050            +    342  9.74e-21 TTCCGACGAG GTTATGGTCATGGTCGATCAAGCGCCAGTGCTGCGCGCT
TTGGGTGTCT
HMPREF1120_02021            +    453  2.85e-16 GTCGTCAATG GTGATGGACTTGGCCCAAAGAGCGCCTCTGGTAGCTGCT
CGAGACTCGG
HMPREF1120_09162            +    264  3.40e-15 TTTGCGCTCT GCGATGGTCATAGCTGTGAATTCGCCGGTGGTTCCTACC
GGGACCAGCC
HMPREF1120_04805            +    356  4.93e-15 ACCGTATAAA GTGCTGACCTTGGCCCAAAAAGGGCCGGGGTATTTCGCC
AAAGGATGGA
--------------------------------------------------------------------------------

--------------------------------------------------------------------------------
        Motif GTKATGGTCWTGGYCSADMAAGCGCCDGTGBTDCBYGCY MEME-10 block diagrams
--------------------------------------------------------------------------------
SEQUENCE NAME            POSITION P-VALUE  MOTIF DIAGRAM
-------------            ----------------  -------------
HMPREF1120_07049                  9.7e-21  280_[-10]_681
HMPREF1120_07050                  9.7e-21  341_[+10]_620
HMPREF1120_02021                  2.8e-16  452_[+10]_509
HMPREF1120_09162                  3.4e-15  263_[+10]_698
HMPREF1120_04805                  4.9e-15  355_[+10]_606
--------------------------------------------------------------------------------

--------------------------------------------------------------------------------
        Motif GTKATGGTCWTGGYCSADMAAGCGCCDGTGBTDCBYGCY MEME-10 in BLOCKS format
--------------------------------------------------------------------------------
BL   MOTIF GTKATGGTCWTGGYCSADMAAGCGCCDGTGBTDCBYGCY width=39 seqs=5
HMPREF1120_07049         (  281) GTTATGGTCATGGTCGATCAAGCGCCAGTGCTGCGCGCT  1
HMPREF1120_07050         (  342) GTTATGGTCATGGTCGATCAAGCGCCAGTGCTGCGCGCT  1
HMPREF1120_02021         (  453) GTGATGGACTTGGCCCAAAGAGCGCCTCTGGTAGCTGCT  1
HMPREF1120_09162         (  264) GCGATGGTCATAGCTGTGAATTCGCCGGTGGTTCCTACC  1
HMPREF1120_04805         (  356) GTGCTGACCTTGGCCCAAAAAGGGCCGGGGTATTTCGCC  1
//

--------------------------------------------------------------------------------

--------------------------------------------------------------------------------
        Motif GTKATGGTCWTGGYCSADMAAGCGCCDGTGBTDCBYGCY MEME-10 position-specific scoring matrix
--------------------------------------------------------------------------------
log-odds matrix: alength= 4 w= 39 n= 449032 bayes= 15.8889 E= 7.8e+003
  -897   -897    196   -897
  -897    -36   -897    172
  -897   -897    122     72
   172    -36   -897   -897
  -897   -897   -897    204
  -897   -897    196   -897
   -28   -897    163   -897
   -28    -36   -897    130
  -897    196   -897   -897
   130   -897   -897     72
  -897   -897   -897    204
   -28   -897    163   -897
  -897   -897    196   -897
  -897    122   -897     72
  -897    163   -897    -28
  -897     64    122   -897
   172   -897   -897    -28
    72   -897    -36     72
   130     64   -897   -897
   172   -897    -36   -897
   172   -897   -897    -28
  -897   -897    163    -28
  -897    163    -36   -897
  -897   -897    196   -897
  -897    196   -897   -897
  -897    196   -897   -897
    72   -897     64    -28
  -897    -36    163   -897
  -897   -897    -36    172
  -897   -897    196   -897
```

```
  -897     64     64    -28
   -28   -897   -897    172
   -28   -897     64     72
  -897    122    -36    -28
  -897     64     64    -28
  -897    122   -897     72
   -28   -897    163   -897
  -897    196   -897   -897
  -897     64   -897    130
--------------------------------------------------------------------------------

--------------------------------------------------------------------------------
	Motif GTKATGGTCWTGGYCSADMAAGCGCCDGTGBTDCBYGCY MEME-10 position-specific probability matrix
--------------------------------------------------------------------------------
letter-probability matrix: alength= 4 w= 39 nsites= 5 E= 7.8e+003 
 0.000000  0.000000  1.000000  0.000000 
 0.000000  0.200000  0.000000  0.800000 
 0.000000  0.000000  0.600000  0.400000 
 0.800000  0.200000  0.000000  0.000000 
 0.000000  0.000000  0.000000  1.000000 
 0.000000  0.000000  1.000000  0.000000 
 0.200000  0.000000  0.800000  0.000000 
 0.200000  0.200000  0.000000  0.600000 
 0.000000  1.000000  0.000000  0.000000 
 0.600000  0.000000  0.000000  0.400000 
 0.000000  0.000000  0.000000  1.000000 
 0.200000  0.000000  0.800000  0.000000 
 0.000000  0.000000  1.000000  0.000000 
 0.000000  0.600000  0.000000  0.400000 
 0.000000  0.800000  0.000000  0.200000 
 0.000000  0.400000  0.600000  0.000000 
 0.800000  0.000000  0.000000  0.200000 
 0.400000  0.000000  0.200000  0.400000 
 0.600000  0.400000  0.000000  0.000000 
 0.800000  0.000000  0.200000  0.000000 
 0.800000  0.000000  0.000000  0.200000 
 0.000000  0.000000  0.800000  0.200000 
 0.000000  0.800000  0.200000  0.000000 
 0.000000  0.000000  1.000000  0.000000 
 0.000000  1.000000  0.000000  0.000000 
 0.000000  1.000000  0.000000  0.000000 
 0.400000  0.000000  0.400000  0.200000 
 0.000000  0.200000  0.800000  0.000000 
 0.000000  0.000000  0.200000  0.800000 
 0.000000  0.000000  1.000000  0.000000 
 0.000000  0.400000  0.400000  0.200000 
 0.200000  0.000000  0.000000  0.800000 
 0.200000  0.000000  0.400000  0.400000 
 0.000000  0.600000  0.200000  0.200000 
 0.000000  0.400000  0.400000  0.200000 
 0.000000  0.600000  0.000000  0.400000 
 0.200000  0.000000  0.800000  0.000000 
 0.000000  1.000000  0.000000  0.000000 
 0.000000  0.400000  0.000000  0.600000 
--------------------------------------------------------------------------------

--------------------------------------------------------------------------------
	Motif GTKATGGTCWTGGYCSADMAAGCGCCDGTGBTDCBYGCY MEME-10 regular expression
--------------------------------------------------------------------------------
G[TC][GT][AC]TG[GA][TAC]C[AT]T[GA]G[CT][CT][GC][AT][ATG][AC][AG][AT][GT][CG]GCC[AGT][GC][TG]G[CGT][TA][GTA]
[CGT][CGT][CT][GA]C[TC]
--------------------------------------------------------------------------------




Time 4928.92 secs.

********************************************************************************
```

```
********************************************************************************
SUMMARY OF MOTIFS
********************************************************************************

--------------------------------------------------------------------------------
	Combined block diagrams: non-overlapping sites with p-value < 0.0001
--------------------------------------------------------------------------------
SEQUENCE NAME            COMBINED P-VALUE  MOTIF DIAGRAM
-------------            ----------------  -------------
HMPREF1120_00418                 2.28e-02  272_[-4(1.81e-05)]_152_\
    [-5(2.14e-05)]_321_[-1(3.49e-06)]_186
HMPREF1120_02408                 3.17e-01  346_[-8(5.64e-05)]_74_\
    [-7(5.08e-05)]_489
HMPREF1120_01131                 1.79e-02  606_[+9(1.06e-05)]_3_[-2(1.32e-05)]_\
    350
HMPREF1120_08232                 4.43e-01  940_[-5(6.14e-05)]_20
HMPREF1120_01106                 1.70e-03  87_[+3(2.38e-05)]_170_\
    [+5(2.21e-08)]_143_[-3(6.86e-05)]_25_[-10(6.75e-05)]_225_[+1(6.99e-06)]_\
    220
HMPREF1120_00342                 5.97e-01  32_[+1(6.99e-06)]_95_[+1(6.99e-06)]_\
    855
HMPREF1120_05503                 2.07e-02  29_[+8(8.01e-05)]_247_\
    [+2(4.12e-05)]_69_[-7(2.16e-05)]_110_[+2(1.13e-06)]_132_[-7(5.40e-06)]_103_\
    [+2(3.57e-06)]_106
HMPREF1120_06548                 7.02e-01  566_[+6(7.41e-05)]_26_\
    [+3(1.18e-05)]_337
HMPREF1120_06442                 4.47e-01  177_[-7(4.92e-05)]_559_\
    [+2(2.50e-05)]_59_[-8(9.84e-05)]_93
HMPREF1120_03392                 2.25e-01  289_[+6(6.07e-05)]_7_[-6(5.33e-05)]_\
    221_[-3(4.91e-05)]_20_[-10(3.49e-05)]_143_[-3(5.82e-05)]_139
HMPREF1120_00104                 7.63e-02  447_[-8(1.10e-05)]_457_\
    [+3(1.60e-05)]_34
HMPREF1120_06522                 2.22e-01  739_[-7(5.08e-05)]_98_\
    [+8(2.92e-05)]_72
HMPREF1120_02117                 2.14e-08  55_[-5(2.20e-05)]_53_[+8(9.84e-05)]_\
    20_[-3(8.71e-05)]_286_[+4(8.61e-05)]_122_[-1(4.28e-05)]_193_[-5(4.25e-11)]_\
    45_[+3(2.31e-08)]_34
HMPREF1120_00243                 2.00e-01  444_[-3(1.02e-06)]_380_\
    [-6(9.77e-05)]_105
HMPREF1120_07631                 4.49e-01  954_[-2(1.44e-05)]_25
HMPREF1120_04377                 7.98e-03  50_[-7(1.99e-05)]_85_[-3(1.54e-06)]_\
    535_[-2(7.70e-06)]_48_[-2(5.76e-05)]_122_[-3(4.91e-05)]_26
HMPREF1120_07528                 1.58e-03  108_[+5(7.27e-06)]_337_\
    [-1(3.49e-06)]_89_[+5(2.38e-05)]_31_[-1(5.35e-05)]_[-1(6.99e-06)]_46_\
    [+3(1.45e-05)]_15_[-1(9.56e-05)]_184_[-8(7.78e-05)]_12
HMPREF1120_00627                 4.08e-04  77_[-5(2.38e-05)]_530_\
    [-5(5.56e-05)]_262_[+1(6.99e-06)]_17_[-3(1.17e-06)]_4
HMPREF1120_06863                 3.33e-01  124_[-2(2.80e-05)]_106_\
    [-8(3.30e-05)]_115_[+6(8.39e-05)]_443_[-4(6.87e-05)]_80
HMPREF1120_08881                 2.52e-01  214_[-8(8.56e-05)]_465_\
    [+7(1.57e-05)]_230
HMPREF1120_04665                 1.83e-01  649_[-7(2.87e-05)]_99_\
    [-2(3.85e-05)]_73_[+7(4.09e-06)]_58
HMPREF1120_08459                 6.37e-01  832_[+10(7.24e-05)]_129
HMPREF1120_03061                 4.68e-03  105_[-7(9.57e-05)]_219_\
    [+9(2.42e-05)]_206_[-1(2.84e-05)]_8_[+3(2.59e-07)]_362
HMPREF1120_07509                 5.35e-01  157_[-7(2.83e-05)]_392_\
    [-2(5.22e-05)]_380
HMPREF1120_06129                 5.09e-04  4_[-4(4.92e-09)]_14_[+1(6.05e-05)]_\
    751_[-7(7.38e-05)]_88_[-1(3.49e-06)]_55
HMPREF1120_05730                 3.35e-04  40_[-2(5.58e-05)]_14_[+2(1.32e-05)]_\
    530_[+5(4.91e-05)]_256_[+9(1.21e-10)]_58
HMPREF1120_01971                 9.44e-01  695_[-1(9.56e-05)]_296
HMPREF1120_05552                 3.02e-01  221_[-7(1.51e-05)]_729
HMPREF1120_02631                 2.08e-03  85_[-7(8.21e-06)]_123_\
    [-1(3.49e-06)]_56_[-3(3.78e-05)]_426_[-10(7.78e-05)]_79_[-8(7.50e-06)]_71
HMPREF1120_06634                 9.84e-01  1000
```

```
HMPREF1120_03639                 7.00e-01  718_[-8(5.93e-05)]_241
HMPREF1120_05086                 4.92e-01  372_[-8(8.56e-05)]_408_\
    [+9(3.05e-05)]_4_[+1(6.38e-05)]_146
HMPREF1120_03771                 2.76e-02  61_[-2(1.38e-05)]_117_\
    [+6(9.63e-05)]_265_[+4(1.65e-05)]_113_[+1(6.05e-05)]_45_[+2(2.07e-05)]_209_\
    [-1(1.77e-05)]_60
HMPREF1120_01653                 2.07e-01  305_[-9(7.86e-05)]_265_\
    [+5(8.22e-05)]_147_[+1(2.14e-05)]_49_[-1(1.05e-05)]_44_[-1(4.61e-05)]_103
HMPREF1120_02703                 2.42e-03  62_[+2(4.57e-05)]_84_\
    [-10(7.04e-05)]_155_[-2(5.76e-05)]_121_[-2(5.22e-05)]_110_[+5(1.42e-05)]_4_\
    [+1(3.21e-05)]_120_[+3(1.76e-06)]_172
HMPREF1120_03835                 2.87e-04  44_[+1(6.99e-06)]_236_\
    [-3(6.32e-05)]_23_[+4(2.15e-07)]_249_[-1(9.56e-05)]_44_[+3(7.61e-06)]_162_\
    [+3(9.42e-05)]_52_[+7(1.73e-05)]_39
HMPREF1120_02790                 5.29e-01  162_[-3(3.15e-05)]_817
HMPREF1120_01095                 1.47e-03  256_[+3(9.42e-05)]_31_\
    [+8(9.89e-06)]_88_[+4(3.17e-06)]_230_[-9(4.76e-07)]_122_[+7(9.72e-05)]_121
HMPREF1120_03541                 8.41e-02  99_[+9(2.64e-05)]_463_\
    [-3(1.06e-05)]_304_[-1(2.14e-05)]_29_[-3(2.61e-05)]_34
HMPREF1120_02172                 1.35e-02  56_[-6(6.07e-05)]_20_[+9(4.48e-06)]_\
    445_[+3(2.38e-05)]_388
HMPREF1120_00689                 8.85e-03  259_[-4(1.30e-05)]_123_\
    [+1(8.86e-05)]_283_[+1(6.99e-06)]_25_[-3(3.45e-05)]_251
HMPREF1120_03048                 5.23e-03  280_[-8(6.42e-07)]_100_\
    [+6(2.59e-05)]_336_[+9(4.19e-06)]_173
HMPREF1120_03880                 6.05e-01  2_[+6(7.95e-06)]_144_[-6(3.89e-05)]_\
    754
HMPREF1120_06931                 8.83e-01  435_[-10(9.63e-05)]_526
HMPREF1120_08377                 1.21e-03  432_[+6(4.13e-05)]_183_\
    [+2(8.15e-11)]_171_[-2(8.48e-05)]_122
HMPREF1120_00312                 4.30e-02  367_[-10(1.79e-05)]_268_\
    [-6(7.62e-05)]_276
HMPREF1120_04902                 7.01e-01  929_[-3(5.41e-06)]_50
HMPREF1120_06823                 1.24e-01  74_[-10(2.58e-05)]_369_\
    [+1(1.05e-05)]_471_[-3(9.42e-05)]_17
HMPREF1120_06746                 1.34e-01  24_[-10(1.68e-05)]_259_\
    [-8(2.01e-05)]_105_[-1(7.44e-05)]_523
HMPREF1120_02714                 5.91e-01  87_[+5(5.42e-05)]_38_[+5(3.81e-05)]_\
    459_[+3(2.87e-05)]_315
HMPREF1120_08389                 1.38e-01  151_[+3(4.50e-05)]_326_\
    [+6(6.63e-06)]_312_[-2(7.96e-05)]_119
HMPREF1120_02139                 9.02e-03  203_[+3(4.50e-05)]_248_\
    [-6(3.83e-05)]_67_[-8(5.22e-05)]_234_[-3(1.31e-05)]_115
HMPREF1120_05939                 4.90e-02  310_[+2(2.54e-06)]_303_\
    [-2(8.57e-08)]_98_[+2(4.89e-05)]_226
HMPREF1120_07217                 3.80e-01  908_[-3(6.32e-05)]_71
HMPREF1120_07962                 2.14e-02  59_[-5(3.62e-05)]_57_[-6(2.97e-05)]_\
    490_[-1(3.49e-06)]_240_[-3(4.91e-05)]_34
HMPREF1120_01351                 6.80e-02  25_[-6(1.18e-05)]_4_[+4(6.73e-05)]_\
    93_[-7(5.58e-05)]_758
HMPREF1120_01636                 9.62e-02  52_[+9(2.35e-05)]_584_\
    [+2(5.74e-06)]_259_[-6(6.34e-05)]_14
HMPREF1120_05323                 5.68e-01  1000
HMPREF1120_04126                 4.34e-03  339_[-3(2.38e-05)]_86_\
    [+5(2.51e-05)]_71_[+1(6.73e-05)]_282_[-7(6.60e-07)]_[-8(4.25e-05)]_61
HMPREF1120_07074                 9.29e-02  211_[+3(4.89e-07)]_399_\
    [-3(9.42e-05)]_348
HMPREF1120_05589                 9.67e-01  1000
HMPREF1120_08630                 1.08e-02  732_[+4(4.12e-08)]_248
HMPREF1120_04575                 8.64e-01  379_[-3(3.15e-05)]_600
HMPREF1120_06595                 1.31e-01  243_[+6(1.64e-05)]_360_\
    [+3(4.50e-05)]_104_[+6(9.24e-05)]_172
HMPREF1120_04938                 7.04e-04  64_[+1(3.91e-05)]_54_[+5(6.05e-06)]_\
    275_[+3(3.45e-05)]_324_[-7(3.72e-06)]_163
HMPREF1120_05123                 1.57e-03  148_[+3(1.02e-06)]_250_\
    [-6(8.39e-05)]_311_[-2(2.41e-08)]_199
HMPREF1120_06825                 2.54e-01  666_[+5(4.32e-05)]_177_\
    [-4(2.86e-05)]_97
HMPREF1120_04438                 3.89e-01  572_[+1(4.28e-05)]_419
```

```
HMPREF1120_03953                  3.84e-01  256_[+3(1.77e-05)]_723
HMPREF1120_02531                  1.16e-04  347_[-4(6.73e-05)]_319_\
    [+4(8.12e-11)]_64_[-9(7.02e-06)]_210
HMPREF1120_05950                  1.08e-03  119_[+7(6.52e-05)]_99_\
    [+3(8.05e-05)]_52_[-9(5.25e-05)]_30_[+1(8.86e-05)]_64_[+10(3.31e-05)]_46_\
    [-8(9.51e-05)]_136_[-8(3.14e-05)]_26_[-6(5.01e-06)]_110_[-1(3.49e-06)]_38
HMPREF1120_01967                  1.51e-02  351_[+3(1.45e-05)]_15_\
    [+3(8.71e-05)]_543_[+1(3.49e-06)]_9_[-2(1.11e-05)]_10
HMPREF1120_05644                  3.04e-03  82_[-2(3.59e-05)]_36_\
    [+10(6.20e-05)]_136_[+4(3.89e-05)]_47_[-6(6.81e-05)]_67_[-2(1.63e-05)]_36_\
    [-9(3.05e-05)]_341_[+1(6.99e-06)]_75
HMPREF1120_01546                  2.58e-01  217_[+6(8.05e-05)]_133_\
    [-2(6.56e-05)]_120_[-4(6.32e-05)]_236_[-2(9.04e-05)]_22_[+1(6.38e-05)]_151
HMPREF1120_00797                  4.77e-01  386_[-1(8.86e-05)]_95_\
    [+2(9.33e-05)]_323_[-3(1.96e-05)]_145
HMPREF1120_08663                  1.60e-02  197_[+2(3.32e-08)]_221_\
    [-1(7.79e-05)]_94_[+3(1.60e-05)]_437
HMPREF1120_05462                  8.82e-01  1000
HMPREF1120_07533                  6.87e-03  118_[-2(4.20e-07)]_228_\
    [-7(2.31e-05)]_33_[-2(9.62e-05)]_134_[+3(9.49e-06)]_68_[+1(4.28e-05)]_297
HMPREF1120_03675                  8.86e-01  1000
HMPREF1120_06581                  8.26e-01  458_[-3(1.45e-05)]_521
HMPREF1120_02779                  1.53e-01  54_[-1(4.98e-05)]_109_\
    [+9(5.11e-05)]_158_[-1(2.47e-05)]_641
HMPREF1120_04883                  2.87e-03  68_[+9(9.39e-06)]_67_[-5(1.07e-05)]_\
    138_[+10(5.38e-05)]_254_[+6(2.97e-05)]_285_[+4(1.99e-06)]_19
HMPREF1120_04910                  1.46e-01  133_[-10(2.53e-05)]_49_\
    [-8(7.55e-05)]_49_[-8(1.38e-05)]_143_[-3(4.13e-05)]_484
HMPREF1120_07397                  2.37e-08  171_[+1(8.86e-05)]_107_\
    [+7(3.85e-17)]_663
HMPREF1120_02089                  4.79e-01  509_[-6(6.52e-05)]_164_\
    [-5(6.29e-05)]_237
HMPREF1120_07479                  8.85e-03  399_[-8(8.27e-05)]_67_\
    [+4(8.97e-05)]_41_[-9(3.45e-08)]_15_[+3(2.16e-05)]_241_[+3(6.86e-05)]_114
HMPREF1120_02715                  1.52e-01  397_[-5(7.27e-06)]_196_\
    [-6(7.30e-05)]_81_[-1(4.98e-05)]_227
HMPREF1120_04920                  1.98e-02  289_[-8(3.07e-05)]_550_\
    [-4(8.05e-07)]_26_[+1(9.56e-05)]_65
HMPREF1120_08523                  9.38e-02  93_[+3(1.31e-05)]_336_\
    [+3(9.49e-06)]_352_[+5(1.47e-06)]_137
HMPREF1120_05339                  3.54e-01  662_[-4(8.44e-05)]_172_\
    [+9(1.21e-05)]_126
HMPREF1120_06790                  6.01e-01  561_[-3(1.96e-05)]_418
HMPREF1120_06758                  2.33e-01  27_[+3(1.45e-05)]_569_\
    [-1(9.56e-05)]_65_[+3(9.49e-06)]_288
HMPREF1120_07664                  9.38e-05  67_[-9(6.43e-05)]_9_[-6(9.63e-05)]_\
    178_[-1(4.28e-05)]_118_[-5(4.01e-09)]_92_[-5(5.70e-05)]_261_[-3(9.42e-05)]_\
    33_[+1(3.49e-06)]_53
HMPREF1120_03399                  1.20e-07  604_[-6(7.95e-06)]_144_\
    [-9(3.26e-09)]_22_[+3(5.69e-07)]_20_[-5(1.34e-05)]_45_[-1(4.28e-05)]_\
    [-1(9.56e-05)]_16
HMPREF1120_02769                  2.77e-02  15_[+8(9.51e-05)]_488_\
    [+3(6.32e-05)]_120_[+4(5.39e-05)]_88_[-2(4.12e-05)]_117_[-7(1.46e-05)]_19
HMPREF1120_03104                  2.69e-01  14_[-5(2.79e-05)]_668_\
    [+7(3.87e-05)]_228
HMPREF1120_05710                  9.01e-03  76_[-1(6.05e-05)]_15_[+3(8.71e-05)]_\
    66_[-1(3.49e-06)]_308_[+4(8.79e-05)]_7_[+9(4.48e-06)]_2_[-2(4.41e-05)]_426
HMPREF1120_02138                  1.61e-01  121_[-3(3.78e-05)]_347_\
    [+5(6.61e-05)]_374_[+8(7.83e-05)]_56
HMPREF1120_05753                  6.82e-01  508_[-3(7.44e-05)]_471
HMPREF1120_07854                  4.21e-01  855_[+9(7.86e-05)]_125
HMPREF1120_04684                  2.56e-01  50_[+1(4.61e-05)]_154_\
    [-2(6.35e-05)]_766
HMPREF1120_00585                  3.35e-02  368_[+1(2.14e-05)]_32_\
    [+6(2.13e-05)]_253_[-2(3.99e-05)]_267
HMPREF1120_06490                  1.08e-08  425_[-6(1.93e-15)]_187_\
    [-1(3.49e-06)]_171_[-10(8.26e-05)]_119
HMPREF1120_02566                  5.69e-02  139_[-4(6.45e-05)]_242_\
    [-3(3.35e-06)]_31_[-1(3.54e-05)]_367_[-3(4.91e-05)]_150
```

```
HMPREF1120_07951                 5.21e-05  210_[+3(1.06e-05)]_17_\
    [+7(8.47e-05)]_50_[+2(3.35e-05)]_88_[+3(7.61e-06)]_214_[-8(8.21e-05)]_73_\
    [-2(2.99e-08)]_9_[-1(6.99e-06)]_155
HMPREF1120_05725                 9.45e-01  811_[-7(5.32e-05)]_139
HMPREF1120_07128                 3.10e-01  445_[-3(1.45e-05)]_133_\
    [-2(9.93e-05)]_102_[-1(9.56e-05)]_269
HMPREF1120_02118                 1.88e-01  158_[-4(5.02e-05)]_109_\
    [+1(6.99e-06)]_292_[-10(8.51e-06)]_373
HMPREF1120_05801                 1.48e-04  20_[-7(4.06e-05)]_48_[+1(3.54e-05)]_\
    136_[+1(6.99e-06)]_54_[+5(6.29e-05)]_125_[-8(8.05e-05)]_154_[-8(2.99e-05)]_\
    200_[-4(3.24e-08)]_53
HMPREF1120_06188                 1.68e-01  496_[+3(9.42e-05)]_154_\
    [-10(3.62e-05)]_174_[+1(6.99e-06)]_107
HMPREF1120_03768                 2.16e-01  284_[-6(8.28e-05)]_383_\
    [+3(6.61e-07)]_1_[+3(1.96e-05)]_4_[+3(6.07e-06)]_215
HMPREF1120_00286                 7.21e-01  324_[+8(9.66e-05)]_635
HMPREF1120_02424                 1.83e-05  98_[+9(1.03e-06)]_172_\
    [-8(1.13e-06)]_113_[+5(5.56e-05)]_82_[-4(2.84e-07)]_[+8(7.86e-05)]_31_\
    [-1(8.86e-05)]_333
HMPREF1120_00652                 2.22e-04  104_[+4(4.76e-07)]_90_\
    [-1(2.84e-05)]_187_[+5(6.45e-05)]_79_[-1(3.49e-06)]_98_[+2(8.48e-05)]_140_\
    [-6(3.60e-06)]_153
HMPREF1120_08713                 8.39e-01  858_[-8(7.25e-05)]_101
HMPREF1120_07632                 2.96e-03  487_[+2(4.89e-05)]_283_\
    [+3(2.85e-08)]_14_[-2(4.20e-07)]_153
HMPREF1120_00149                 6.31e-01  483_[-1(4.98e-05)]_508
HMPREF1120_02287                 6.01e-01  299_[-1(3.54e-05)]_219_\
    [-6(5.09e-05)]_423
HMPREF1120_04393                 1.02e-01  276_[+2(8.48e-05)]_467_\
    [+1(8.86e-05)]_55_[+3(1.45e-05)]_151
HMPREF1120_06862                 5.88e-01  353_[+2(2.07e-05)]_348_\
    [+4(6.87e-05)]_258
HMPREF1120_06895                 4.67e-01  1000
HMPREF1120_01920                 1.95e-01  267_[-7(2.64e-05)]_320_\
    [-2(4.72e-05)]_342
HMPREF1120_06329                 2.83e-03  24_[+2(2.32e-05)]_88_[+4(1.06e-05)]_\
    54_[+6(2.40e-05)]_42_[+6(2.79e-05)]_463_[-2(6.35e-05)]_70_[-10(4.56e-05)]_\
    58
HMPREF1120_05252                 6.01e-01  439_[-5(6.77e-05)]_521
HMPREF1120_02898                 1.20e-01  693_[+7(7.05e-05)]_23_\
    [+7(1.91e-06)]_16_[-9(5.82e-05)]_148
HMPREF1120_00045                 1.73e-01  75_[+2(5.58e-05)]_90_[+3(8.71e-05)]_\
    25_[-10(8.38e-05)]_316_[+1(6.38e-05)]_395_[-1(9.56e-05)]
HMPREF1120_05635                 4.32e-01  247_[-9(9.69e-05)]_132_\
    [-1(3.91e-05)]_231_[+6(8.75e-05)]_311
HMPREF1120_03442                 1.13e-01  75_[-7(6.60e-07)]_317_\
    [+9(4.71e-05)]_538
HMPREF1120_01547                 1.96e-01  8_[+5(7.11e-05)]_519_[+3(1.31e-05)]_\
    308_[-1(6.38e-05)]_22_[+2(9.04e-05)]_52
HMPREF1120_07039                 1.81e-01  158_[-9(6.28e-05)]_131_\
    [-5(6.14e-05)]_179_[-7(1.51e-05)]_422
HMPREF1120_02634                 1.14e-02  18_[+1(3.49e-06)]_123_\
    [+7(8.21e-06)]_306_[+3(3.78e-06)]_4_[-5(7.29e-05)]_429
HMPREF1120_03876                 5.85e-01  73_[+9(6.43e-05)]_409_\
    [+5(3.02e-05)]_458
HMPREF1120_02275                 5.85e-02  237_[-3(2.87e-05)]_579_\
    [+3(7.61e-06)]_121_[-3(3.58e-07)]
HMPREF1120_03066                 3.51e-01  172_[-4(8.79e-05)]_21_\
    [+3(2.16e-05)]_6_[+3(5.82e-05)]_739
HMPREF1120_02373                 2.75e-01  131_[-1(8.86e-05)]_423_\
    [+10(5.15e-05)]_341_[-3(8.51e-06)]_36
HMPREF1120_06864                 6.07e-05  39_[+5(4.11e-05)]_309_\
    [-9(1.03e-06)]_81_[+2(2.24e-05)]_57_[+2(4.89e-05)]_15_[+9(4.94e-10)]_377
HMPREF1120_02274                 4.05e-03  344_[+3(3.58e-07)]_116_\
    [-3(7.61e-06)]_498
HMPREF1120_08525                 3.31e-02  587_[+10(4.63e-05)]_119_\
    [-8(6.61e-06)]_159_[+2(3.35e-05)]_34
HMPREF1120_07050                 1.77e-80  53_[+8(2.34e-25)]_53_[-5(6.15e-15)]_\
    25_[+6(1.40e-24)]_22_[+7(5.12e-25)]_7_[+10(9.74e-21)]_3_[+3(5.35e-05)]_63_\
```

```
    [-3(7.61e-06)]_202_[-4(5.14e-05)]_141_[+9(2.35e-05)]_129
HMPREF1120_05273                 1.39e-01  356_[-8(1.86e-05)]_92_\
    [-10(4.92e-05)]_137_[+1(7.79e-05)]_326
HMPREF1120_07048                 1.19e-01  96_[+9(7.86e-05)]_655_\
    [+1(1.77e-05)]_220
HMPREF1120_04516                 2.06e-01  203_[-7(4.26e-05)]_333_\
    [-9(6.89e-05)]_179_[+1(2.47e-05)]_206
HMPREF1120_07996                 1.55e-03  122_[+7(1.59e-05)]_336_\
    [+1(3.49e-06)]_232_[-2(7.80e-08)]_127_[-1(1.05e-05)]_94
HMPREF1120_03503                 7.59e-02  84_[-10(1.13e-05)]_351_\
    [+3(4.81e-06)]_505
HMPREF1120_02653                 4.01e-01  402_[-4(6.32e-05)]_32_\
    [-1(1.77e-05)]_537
HMPREF1120_01129                 8.76e-01  809_[+2(8.76e-05)]_170
HMPREF1120_01052                 3.48e-01  669_[-3(2.38e-05)]_216_\
    [-2(6.56e-05)]_73
HMPREF1120_01536                 8.09e-01  51_[+10(8.51e-05)]_755_\
    [-3(7.44e-05)]_134
HMPREF1120_06676                 7.22e-02  79_[-6(1.00e-05)]_339_\
    [+5(1.87e-05)]_209_[+5(4.22e-05)]_243
HMPREF1120_08308                 4.57e-02  166_[+10(1.57e-05)]_3_\
    [+5(3.62e-05)]_183_[-1(6.99e-06)]_560
HMPREF1120_04375                 9.14e-01  1000
HMPREF1120_09262                 9.32e-03  89_[+7(6.94e-05)]_126_\
    [+5(7.29e-05)]_191_[+1(1.05e-05)]_34_[-2(4.87e-07)]_98_[-1(3.54e-05)]_56_\
    [+1(6.99e-06)]_268
HMPREF1120_00244                 2.09e-01  26_[+6(9.77e-05)]_380_\
    [+3(1.02e-06)]_523
HMPREF1120_06537                 5.90e-02  216_[-8(2.67e-05)]_160_\
    [+4(5.02e-05)]_48_[+8(6.97e-05)]_285_[-3(7.61e-06)]_157_[-1(5.35e-05)]_2
HMPREF1120_00235                 2.55e-02  171_[+6(1.00e-05)]_356_\
    [-4(1.12e-05)]_108_[-3(5.35e-05)]_110_[+8(2.62e-05)]_123
HMPREF1120_01435                 2.03e-01  178_[-3(1.06e-05)]_801
HMPREF1120_01637                 1.31e-01  68_[-4(9.74e-05)]_246_\
    [-3(9.42e-05)]_117_[-3(7.44e-05)]_3_[-3(4.91e-05)]_134_[+9(3.31e-06)]_69_\
    [+3(8.71e-05)]_2_[-5(9.05e-05)]_197
HMPREF1120_07508                 5.61e-01  361_[+3(3.45e-05)]_200_\
    [-3(8.71e-05)]_397
HMPREF1120_04934                 1.10e-05  40_[+2(3.71e-13)]_[+2(3.71e-13)]_\
    [+2(3.71e-13)]_32_[+2(3.71e-13)]_[+2(3.71e-13)]_[+2(3.71e-13)]_\
    [+2(3.71e-13)]_309_[-2(4.27e-05)]_150_[-1(2.47e-05)]_91_[-2(4.53e-07)]_107_\
    [-2(2.80e-05)]_52
HMPREF1120_02072                 3.92e-02  168_[-5(8.20e-06)]_294_\
    [+3(3.78e-06)]_45_[+6(3.89e-05)]_382
HMPREF1120_02791                 8.76e-03  29_[+9(2.17e-05)]_176_\
    [-10(9.94e-05)]_90_[+8(7.52e-05)]_139_[+9(6.28e-05)]_271_[+10(8.36e-06)]_\
    55_[+3(1.31e-05)]_60
HMPREF1120_04834                 3.46e-01  128_[+5(6.14e-05)]_152_\
    [+3(7.61e-06)]_659
HMPREF1120_01817                 1.57e-09  25_[-6(4.52e-05)]_127_\
    [+1(1.05e-05)]_8_[+3(1.60e-05)]_43_[+5(2.14e-05)]_610_[+7(1.35e-14)]_17
HMPREF1120_03202                 1.48e-02  80_[+3(2.29e-06)]_197_\
    [-6(3.61e-05)]_154_[+7(1.27e-05)]_27_[-5(1.92e-05)]_92_[-3(3.78e-05)]_268
HMPREF1120_07674                 7.63e-02  439_[-2(3.85e-05)]_69_\
    [-5(5.16e-05)]_105_[+3(4.81e-06)]_116_[-8(4.47e-05)]_148
HMPREF1120_07633                 1.55e-03  92_[+2(4.20e-07)]_14_[-3(2.85e-08)]_\
    283_[-2(4.89e-05)]_548
HMPREF1120_01995                 5.35e-02  88_[-2(5.58e-05)]_132_\
    [+3(9.42e-05)]_68_[-2(1.11e-05)]_137_[+5(4.91e-05)]_413_[+10(5.00e-05)]_20
HMPREF1120_00159                 3.48e-01  395_[+1(7.44e-05)]_463_\
    [+2(2.60e-05)]_112
HMPREF1120_08773                 6.17e-01  638_[+1(1.77e-05)]_353
HMPREF1120_04488                 5.98e-01  117_[+2(4.72e-05)]_862
HMPREF1120_03846                 1.15e-07  339_[+5(1.89e-13)]_334_\
    [+4(7.62e-05)]_67_[-9(4.45e-05)]_73_[-1(1.77e-05)]_98
HMPREF1120_00041                 3.79e-03  203_[-8(8.75e-05)]_278_\
    [+1(6.99e-06)]_40_[+5(6.77e-05)]_141_[-10(2.92e-05)]_152_[+3(1.35e-06)]_36
HMPREF1120_05330                 6.24e-01  449_[-2(3.35e-05)]_530
HMPREF1120_07767                 7.29e-01  110_[+8(4.58e-05)]_849
```

```
HMPREF1120_01851                 1.16e-01  425_[+8(6.61e-06)]_398_\
    [+2(5.46e-06)]_115
HMPREF1120_06902                 2.85e-04  550_[+5(3.16e-10)]_47_\
    [-8(5.64e-05)]_87_[+7(5.24e-05)]_185
HMPREF1120_09146                 5.11e-01  258_[+10(9.05e-05)]_346_\
    [-7(1.68e-05)]_307
HMPREF1120_03095                 1.76e-01  257_[-1(6.99e-06)]_734
HMPREF1120_07560                 2.52e-11  40_[-5(1.07e-11)]_212_\
    [+1(1.77e-05)]_207_[-3(1.56e-07)]_294_[-4(5.88e-08)]_2_[+9(2.87e-05)]_135
HMPREF1120_00546                 2.44e-01  205_[-8(5.64e-05)]_17_\
    [-3(6.32e-05)]_249_[+8(9.84e-05)]_58_[-3(1.06e-05)]_317_[+9(8.85e-05)]_10
HMPREF1120_04401                 3.22e-01  18_[+9(2.64e-05)]_191_\
    [+9(3.53e-05)]_315_[-4(2.46e-05)]_416
HMPREF1120_03941                 1.43e-05  76_[-1(6.99e-06)]_6_[-1(1.05e-05)]_\
    206_[-2(6.51e-07)]_379_[-8(7.03e-05)]_12_[+2(2.52e-10)]_15_[+2(9.29e-06)]_\
    33_[-1(3.21e-05)]_34_[-3(5.41e-06)]_87
HMPREF1120_05534                 1.33e-01  355_[-8(4.01e-06)]_531_\
    [+10(7.56e-05)]_34
HMPREF1120_01968                 1.18e-01  806_[-10(7.67e-05)]_35_\
    [+1(3.54e-05)]_60_[+2(1.11e-05)]_9_[-1(3.49e-06)]_12
HMPREF1120_05435                 7.72e-03  172_[-9(1.91e-05)]_592_\
    [+5(8.05e-09)]_176
HMPREF1120_02359                 3.22e-01  254_[-1(3.49e-06)]_737
HMPREF1120_04788                 5.46e-01  316_[-1(7.09e-05)]_527_\
    [-9(2.17e-05)]_128
HMPREF1120_05477                 3.21e-01  531_[+3(8.71e-05)]_131_\
    [-6(6.25e-05)]_154_[-8(2.14e-05)]_72
HMPREF1120_06974                 1.15e-03  560_[+5(6.45e-05)]_68_\
    [-4(1.49e-09)]_254_[+9(4.45e-05)]_38
HMPREF1120_04831                 2.75e-01  7_[+3(5.41e-06)]_972
HMPREF1120_02501                 7.19e-02  88_[+9(4.20e-05)]_107_\
    [+4(1.06e-05)]_305_[+4(3.17e-07)]_137_[-9(4.71e-05)]_283
HMPREF1120_04925                 3.45e-01  16_[-10(8.51e-05)]_693_\
    [+9(8.52e-05)]_156_[+8(8.65e-05)]_35
HMPREF1120_02021                 3.88e-09  75_[-9(3.14e-05)]_357_\
    [+10(2.85e-16)]_331_[-1(3.49e-06)]_169
HMPREF1120_06363                 8.49e-03  18_[+3(1.77e-05)]_24_\
    [+10(5.00e-05)]_225_[+7(4.54e-05)]_105_[-7(6.23e-05)]_126_[+3(8.84e-07)]_\
    147_[-1(3.21e-05)]_165
HMPREF1120_07673                 1.23e-01  32_[-10(9.48e-05)]_8_[-5(2.58e-05)]_\
    476_[-5(8.84e-05)]_223_[+9(1.81e-05)]_122
HMPREF1120_07049                 4.88e-79  172_[+3(7.61e-06)]_63_\
    [-3(5.35e-05)]_3_[-10(9.74e-21)]_7_[-7(5.12e-25)]_22_[-6(1.40e-24)]_25_\
    [+5(6.15e-15)]_53_[-8(2.34e-25)]_209_[-3(4.27e-06)]_163
HMPREF1120_07792                 8.36e-02  380_[-3(2.61e-06)]_470_\
    [+7(1.11e-05)]_24_[+5(6.94e-05)]_15
HMPREF1120_03151                 1.47e-02  204_[-2(1.14e-08)]_775
HMPREF1120_08650                 2.52e-02  61_[-7(8.35e-05)]_457_\
    [+3(7.44e-05)]_140_[+1(3.49e-06)]_186_[-3(4.91e-05)]_55
HMPREF1120_06770                 2.50e-05  15_[-5(8.91e-11)]_59_[+1(8.86e-05)]_\
    24_[+10(5.46e-05)]_71_[+7(7.50e-05)]_353_[-4(5.91e-05)]_259_[-5(8.84e-05)]_\
    21
HMPREF1120_07390                 3.48e-01  364_[+5(1.27e-05)]_596
HMPREF1120_05935                 2.61e-02  41_[+10(9.05e-05)]_41_\
    [+3(2.96e-06)]_79_[-8(2.08e-05)]_111_[+4(8.44e-05)]_493_[+3(5.35e-05)]_93
HMPREF1120_01293                 3.60e-01  65_[-8(4.89e-05)]_215_\
    [+7(2.83e-05)]_146_[+8(7.25e-05)]_212_[+7(7.38e-05)]_180
HMPREF1120_07658                 2.01e-04  82_[+3(4.27e-06)]_25_[+7(4.12e-05)]_\
    605_[+2(1.65e-09)]_196
HMPREF1120_06441                 1.08e-15  192_[-6(1.31e-15)]_162_\
    [-5(1.21e-11)]_206_[-8(7.83e-05)]_60_[-2(7.34e-06)]_186_[+3(4.27e-06)]_21
HMPREF1120_00898                 2.10e-01  161_[-7(5.76e-05)]_232_\
    [-1(2.14e-05)]_11_[+2(3.12e-05)]_65_[+2(1.84e-05)]_319_[-1(2.47e-05)]_102
HMPREF1120_08578                 1.37e-01  510_[+8(8.27e-05)]_107_\
    [-9(3.64e-05)]_177_[+6(4.32e-05)]_95
HMPREF1120_06580                 2.26e-01  148_[-10(5.07e-05)]_10_\
    [-2(5.95e-05)]_565_[+3(1.45e-05)]_196
HMPREF1120_07908                 2.77e-05  11_[+7(2.47e-05)]_140_\
    [+3(1.77e-05)]_92_[+9(4.58e-05)]_300_[-1(2.14e-05)]_117_[-9(3.91e-09)]_220
```

```
HMPREF1120_07157                  1.27e-01  487_[-8(3.66e-05)]_21_\
    [-3(5.82e-05)]_317_[-1(4.28e-05)]_104
HMPREF1120_06748                  3.59e-03  244_[-8(1.88e-05)]_415_\
    [-2(1.45e-09)]_279
HMPREF1120_01579                  9.93e-02  261_[+8(6.39e-05)]_53_\
    [-7(8.87e-05)]_346_[-1(3.49e-06)]_240
HMPREF1120_07981                  7.12e-01  455_[-3(7.44e-05)]_524
HMPREF1120_04574                  9.47e-09  180_[-3(2.87e-05)]_298_\
    [+3(2.38e-05)]_210_[+1(3.91e-05)]_96_[+9(5.97e-05)]_23_[+7(7.94e-15)]_72
HMPREF1120_03688                  1.87e-01  621_[-7(8.35e-05)]_39_\
    [-3(1.60e-05)]_146_[+10(1.30e-05)]_84
HMPREF1120_03067                  8.84e-01  1000
HMPREF1120_08344                  4.27e-04  39_[+1(7.44e-05)]_156_\
    [-5(6.94e-05)]_32_[-6(4.13e-05)]_217_[-3(2.38e-05)]_152_[+2(5.74e-06)]_41_\
    [+3(6.61e-07)]_201
HMPREF1120_04843                  9.77e-02  368_[-1(9.56e-05)]_483_\
    [-1(3.91e-05)]_32_[-5(1.63e-06)]_59
HMPREF1120_06583                  1.08e-01  322_[+8(2.57e-05)]_435_\
    [+7(1.82e-05)]_108_[-3(2.87e-05)]_23
HMPREF1120_01375                  1.71e-01  28_[-3(6.86e-05)]_56_[-7(8.22e-05)]_\
    184_[-1(3.49e-06)]_652
HMPREF1120_08154                  2.19e-02  183_[-3(3.15e-05)]_267_\
    [-3(4.81e-06)]_20_[+1(3.49e-06)]_255_[-10(6.65e-05)]_185
HMPREF1120_02698                  1.06e-02  56_[-7(3.23e-05)]_22_[+9(6.12e-05)]_\
    130_[+4(1.76e-05)]_72_[+3(3.78e-05)]_234_[-1(6.99e-06)]_253_[+9(6.89e-05)]_\
    93
HMPREF1120_08138                  2.24e-01  157_[-1(9.56e-05)]_243_\
    [+5(5.70e-05)]_150_[-2(7.00e-05)]_6_[-1(3.49e-06)]_313_[-1(4.98e-05)]_43
HMPREF1120_08448                  4.60e-03  55_[+2(4.43e-06)]_121_\
    [+7(1.95e-05)]_253_[+4(1.56e-05)]_337_[-1(2.84e-05)]_25_[+3(8.71e-05)]_13_\
    [+2(3.12e-05)]_54
HMPREF1120_08742                  1.48e-01  6_[+1(1.77e-05)]_635_[-3(2.16e-05)]_\
    227_[-2(8.48e-05)]_81
HMPREF1120_00007                  1.74e-01  366_[+9(5.53e-05)]_243_\
    [-4(4.88e-06)]_98_[+7(3.62e-05)]_203
HMPREF1120_02578                  6.68e-02  430_[-1(3.21e-05)]_225_\
    [+3(2.87e-05)]_40_[+6(4.52e-05)]_188_[-1(6.99e-06)]_28
HMPREF1120_02127                  8.15e-04  571_[-5(8.05e-09)]_200_\
    [-1(2.14e-05)]_18_[+9(2.56e-05)]_142
HMPREF1120_06618                  7.34e-01  1000
HMPREF1120_02904                  6.16e-05  47_[-7(3.80e-05)]_164_\
    [-2(6.35e-05)]_49_[-2(5.40e-05)]_168_[+4(1.12e-09)]_16_[+2(9.33e-05)]_129_\
    [+2(6.78e-05)]_67_[-3(9.42e-05)]_131_[-3(9.49e-06)]_33
HMPREF1120_00716                  4.46e-03  160_[+2(1.21e-05)]_280_\
    [-4(4.76e-07)]_79_[-2(6.56e-05)]_28_[+5(9.49e-05)]_207_[-3(2.38e-05)]_123
HMPREF1120_06341                  6.51e-02  248_[-4(5.91e-05)]_124_\
    [-5(1.17e-05)]_43_[+7(4.54e-05)]_475
HMPREF1120_04563                  1.45e-05  32_[+3(4.89e-07)]_9_[+3(2.38e-05)]_\
    152_[-2(3.98e-06)]_33_[+2(5.95e-05)]_16_[+2(6.46e-08)]_33_[+4(4.08e-06)]_\
    88_[+3(8.05e-05)]_491
HMPREF1120_06646                  8.44e-01  852_[+2(9.62e-05)]_127
HMPREF1120_02776                  1.43e-13  387_[+4(1.20e-10)]_323_\
    [+1(3.49e-06)]_36_[-9(3.42e-09)]_79_[-1(6.99e-06)]_10_[+2(6.99e-07)]_55_\
    [+3(7.63e-08)]_10
HMPREF1120_06679                  2.37e-01  208_[+1(6.99e-06)]_100_\
    [+7(9.29e-05)]_156_[-3(5.35e-05)]_456
HMPREF1120_05155                  3.64e-01  422_[-7(1.25e-05)]_387_\
    [-7(5.32e-05)]_91
HMPREF1120_01512                  4.08e-01  623_[+2(8.48e-05)]_214
HMPREF1120_01172                  5.09e-02  99_[-7(9.01e-05)]_66_[+5(1.11e-06)]_\
    280_[-8(3.85e-05)]_424
HMPREF1120_09135                  5.17e-01  707_[+7(7.16e-05)]_243
HMPREF1120_08781                  1.30e-02  47_[+2(5.22e-05)]_260_\
    [+7(4.40e-05)]_194_[+5(2.95e-07)]_329_[-3(2.61e-05)]_38
HMPREF1120_01519                  1.23e-02  71_[-1(4.61e-05)]_253_\
    [+5(2.72e-05)]_[-8(7.33e-05)]_474_[+4(6.57e-06)]_92
HMPREF1120_00622                  2.19e-01  8_[+5(8.84e-05)]_375_[+5(5.56e-05)]_\
    71_[+2(3.99e-05)]_180_[+5(5.56e-05)]_150_[-4(2.11e-05)]_55
HMPREF1120_03297                  2.09e-02  96_[+7(4.84e-05)]_379_\
```

```
    [-4(6.75e-06)]_270_[-10(4.14e-05)]_20_[+1(1.77e-05)]_117
HMPREF1120_08211                 5.99e-02  309_[+3(1.02e-06)]_350_\
    [+10(9.48e-05)]_281
HMPREF1120_05905                 8.51e-03  153_[+3(3.78e-05)]_133_\
    [+7(1.79e-05)]_165_[+1(8.86e-05)]_191_[+9(1.91e-05)]_163_[+6(8.49e-06)]_45
HMPREF1120_03649                 1.40e-03  [+10(2.31e-05)]_119_[-1(3.49e-06)]_\
    105_[-1(3.49e-06)]_88_[+3(3.78e-05)]_334_[-3(1.76e-06)]_177_[+6(7.72e-05)]_\
    28
HMPREF1120_00621                 8.72e-02  219_[+1(6.38e-05)]_201_\
    [+8(1.36e-05)]_60_[+5(9.71e-05)]_138_[-8(8.56e-05)]_251
HMPREF1120_01082                 1.21e-01  410_[+6(2.21e-06)]_144_\
    [+3(1.96e-05)]_375
HMPREF1120_08679                 4.78e-02  331_[+3(5.41e-06)]_558_\
    [+4(1.71e-05)]_70
HMPREF1120_06393                 2.72e-03  225_[+3(2.96e-06)]_9_[-1(3.91e-05)]_\
    88_[-3(9.42e-05)]_290_[-2(1.47e-07)]_248_[+3(2.38e-05)]_47
HMPREF1120_03733                 9.47e-01  1000
HMPREF1120_00978                 5.65e-02  199_[+4(9.35e-05)]_89_\
    [+9(7.69e-05)]_207_[-7(2.35e-05)]_16_[-5(8.84e-05)]_230_[-5(4.01e-05)]_4_\
    [+7(9.29e-05)]_35
HMPREF1120_00006                 5.54e-02  44_[-5(4.32e-05)]_35_[+8(5.54e-05)]_\
    469_[-7(3.62e-05)]_98_[+4(4.88e-06)]_203
HMPREF1120_06138                 1.14e-02  [+2(3.72e-05)]_111_[-7(2.47e-05)]_\
    103_[-3(1.06e-05)]_368_[+1(3.91e-05)]_14_[-5(4.91e-05)]_263
HMPREF1120_04842                 6.22e-01  529_[-8(2.67e-05)]_430
HMPREF1120_02509                 1.37e-11  635_[+3(4.50e-05)]_29_\
    [-6(1.48e-20)]_42_[-3(4.81e-06)]_202
HMPREF1120_08480                 3.00e-02  107_[-8(7.19e-05)]_21_\
    [-2(3.33e-07)]_27_[+8(7.08e-05)]_429_[+3(5.82e-05)]_292
HMPREF1120_00717                 2.58e-02  372_[+3(2.01e-06)]_158_\
    [-10(3.03e-05)]_183_[-1(2.47e-05)]_141_[+10(6.65e-05)]_38
HMPREF1120_07062                 1.57e-04  176_[-9(1.70e-06)]_220_\
    [+3(5.35e-05)]_37_[+10(3.81e-05)]_229_[+3(6.32e-08)]_4_[+7(8.51e-06)]_56_\
    [-9(4.20e-05)]_107
HMPREF1120_08684                 2.80e-01  231_[-1(2.14e-05)]_760
HMPREF1120_05439                 1.17e-02  66_[+5(2.72e-05)]_519_\
    [+5(7.04e-09)]_335
HMPREF1120_07638                 4.07e-01  330_[-7(7.50e-05)]_137_\
    [-9(2.56e-05)]_463
HMPREF1120_00405                 9.60e-02  239_[+9(2.78e-06)]_180_\
    [+3(3.45e-05)]_540
HMPREF1120_08447                 1.32e-03  395_[-7(1.27e-05)]_3_[+6(6.16e-05)]_\
    63_[-3(5.35e-05)]_19_[-2(3.59e-05)]_25_[-1(3.54e-05)]_20_[-1(3.49e-06)]_\
    160_[-1(3.49e-06)]_120_[-1(3.49e-06)]_17
HMPREF1120_01764                 4.73e-02  21_[+2(5.40e-05)]_49_[+7(2.83e-05)]_\
    169_[+3(1.45e-05)]_569_[+6(1.26e-05)]_50
HMPREF1120_04550                 1.05e-05  162_[+6(3.50e-05)]_11_\
    [+10(7.77e-06)]_25_[+9(2.89e-06)]_7_[-3(9.49e-06)]_7_[+1(3.54e-05)]_64_\
    [+2(4.57e-05)]_328_[-10(8.77e-05)]_51_[+6(1.02e-05)]_96
HMPREF1120_02510                 4.82e-13  175_[-9(9.69e-05)]_111_\
    [+10(7.24e-05)]_118_[-2(5.05e-05)]_178_[-3(3.78e-06)]_189_[+3(4.81e-06)]_\
    42_[+6(1.48e-20)]_15
HMPREF1120_04120                 1.71e-01  445_[-3(5.82e-05)]_128_\
    [-4(5.91e-05)]_333_[+8(2.85e-05)]_12
HMPREF1120_03732                 9.41e-01  1000
HMPREF1120_02286                 5.95e-02  158_[+1(2.47e-05)]_123_\
    [-10(4.68e-06)]_174_[+4(4.21e-05)]_254_[+1(6.99e-06)]_15_[-1(3.49e-06)]_\
    190
HMPREF1120_03724                 2.77e-01  1_[-7(9.30e-06)]_872_[-8(9.66e-05)]_\
    36
HMPREF1120_06645                 2.10e-03  14_[-10(3.20e-05)]_196_\
    [+10(2.98e-05)]_76_[+2(7.10e-08)]_75_[-2(9.41e-08)]_342_[-1(3.49e-06)]_83_\
    [-5(9.27e-05)]_45
HMPREF1120_04812                 8.57e-02  100_[+3(9.42e-05)]_628_\
    [-4(5.02e-05)]_98_[+7(2.60e-05)]_83
HMPREF1120_05987                 6.61e-01  664_[-3(1.18e-05)]_17_\
    [-3(4.13e-05)]_277
HMPREF1120_07793                 1.14e-04  301_[-3(5.41e-06)]_58_\
    [-2(1.08e-09)]_14_[+5(4.11e-05)]_545
```

```
HMPREF1120_00375                 3.27e-02  270_[+5(1.68e-05)]_9_[+1(4.28e-05)]_\
    255_[+5(3.81e-05)]_62_[+3(8.05e-05)]_50_[-6(2.33e-05)]_151_[-1(6.99e-06)]_\
    34
HMPREF1120_01149                 2.75e-01  115_[+2(3.85e-05)]_341_\
    [+5(2.58e-05)]_135_[+2(4.89e-05)]_327
HMPREF1120_02526                 4.74e-01  224_[-9(7.22e-05)]_453_\
    [-10(3.31e-05)]_101_[+9(3.43e-05)]_143
HMPREF1120_02305                 2.89e-04  15_[+2(9.93e-05)]_13_[+2(5.74e-06)]_\
    131_[-4(7.10e-06)]_3_[+9(9.01e-06)]_171_[+1(3.49e-06)]_456_[-1(6.99e-06)]_\
    68_[+3(2.61e-05)]_22
HMPREF1120_06671                 1.45e-04  173_[-7(4.01e-06)]_122_\
    [-2(1.56e-06)]_63_[+10(7.90e-05)]_69_[-2(1.06e-06)]_80_[+1(1.05e-05)]_141_\
    [+2(9.93e-05)]_67_[+7(7.27e-05)]_74
HMPREF1120_05694                 1.93e-01  99_[-6(9.37e-05)]_270_\
    [+3(5.82e-05)]_69_[-8(2.26e-05)]_450
HMPREF1120_09019                 2.51e-09  13_[+7(4.52e-15)]_54_[-7(9.87e-05)]_\
    494_[+7(9.01e-05)]_69_[+1(3.49e-06)]_211
HMPREF1120_06616                 6.07e-01  13_[+6(5.48e-05)]_937
HMPREF1120_05142                 4.27e-03  360_[-1(6.99e-06)]_94_\
    [-8(1.18e-05)]_54_[+3(5.69e-07)]_317_[-4(9.95e-05)]_84
HMPREF1120_07623                 1.08e-01  502_[+1(3.49e-06)]_489
HMPREF1120_09134                 3.50e-12  72_[+10(3.55e-05)]_157_\
    [+1(3.49e-06)]_120_[+1(3.49e-06)]_193_[-6(7.94e-05)]_7_[+3(1.56e-07)]_242_\
    [+7(8.85e-15)]_31
HMPREF1120_05354                 3.63e-05  316_[-3(2.87e-05)]_50_\
    [+3(1.86e-07)]_146_[+3(1.18e-05)]_4_[-1(6.99e-06)]_93_[-3(2.29e-06)]_31_\
    [-1(7.09e-05)]_68_[-8(6.85e-05)]_23_[-1(6.99e-06)]_14_[-2(1.89e-07)]_3_\
    [-2(5.24e-07)]_58
HMPREF1120_01508                 1.60e-01  313_[-2(5.40e-05)]_90_\
    [-4(4.10e-05)]_149_[-10(3.37e-05)]_118_[+3(9.42e-05)]_229
HMPREF1120_00838                 3.09e-01  578_[+7(9.72e-05)]_11_\
    [+4(8.10e-05)]_240_[-2(3.47e-05)]_80
HMPREF1120_07321                 6.37e-02  663_[+3(1.06e-05)]_316
HMPREF1120_02454                 1.94e-05  11_[-1(3.54e-05)]_155_\
    [-1(1.05e-05)]_262_[+2(8.22e-05)]_99_[-3(1.96e-05)]_197_[+9(2.27e-08)]_71_\
    [+6(3.35e-05)]_75
HMPREF1120_06644                 3.18e-02  253_[+3(3.45e-05)]_46_\
    [+5(6.14e-05)]_85_[+10(8.77e-05)]_181_[+5(9.27e-05)]_83_[+1(3.49e-06)]_203
HMPREF1120_08477                 2.53e-01  96_[+2(6.78e-05)]_734_\
    [-7(1.32e-05)]_99
HMPREF1120_05234                 2.35e-01  75_[-3(4.91e-05)]_137_\
    [+1(6.99e-06)]_285_[-9(4.97e-05)]_56_[+1(7.79e-05)]_388
HMPREF1120_03386                 2.29e-02  16_[-3(2.61e-05)]_412_\
    [-3(8.84e-07)]_192_[+1(2.47e-05)]_256_[-2(3.99e-05)]_52
HMPREF1120_05842                 1.92e-01  288_[-3(1.76e-06)]_691
HMPREF1120_00264                 1.93e-04  117_[+9(1.78e-11)]_341_\
    [-1(6.05e-05)]_266_[-1(3.49e-06)]_238
HMPREF1120_06777                 2.84e-03  399_[-2(1.77e-05)]_34_\
    [+1(3.49e-06)]_93_[+1(6.99e-06)]_326_[+2(5.05e-05)]_6_[+3(5.41e-06)]_61
HMPREF1120_04224                 7.75e-03  162_[+3(6.32e-05)]_33_\
    [+8(5.80e-06)]_43_[-8(6.94e-06)]_14_[-8(2.43e-05)]_382_[+2(8.22e-05)]_63_\
    [-1(3.49e-06)]_129
HMPREF1120_01423                 1.71e-01  280_[+8(5.11e-05)]_124_\
    [+9(5.67e-05)]_282_[+2(8.48e-05)]_14_[+4(2.61e-05)]_198
HMPREF1120_06279                 1.44e-02  166_[-5(1.31e-05)]_442_\
    [-3(1.17e-06)]_331
HMPREF1120_05665                 5.35e-02  21_[-3(6.32e-05)]_362_\
    [-2(5.22e-05)]_199_[+8(3.87e-06)]_95_[+4(8.44e-05)]_132_[-3(1.77e-05)]_67
HMPREF1120_08115                 4.56e-01  195_[-1(1.05e-05)]_654_\
    [-1(6.73e-05)]_133
HMPREF1120_05585                 1.42e-02  21_[-5(4.28e-06)]_177_\
    [+8(2.71e-06)]_326_[-2(4.41e-05)]_374
HMPREF1120_02014                 8.28e-01  1000
HMPREF1120_03609                 1.29e-01  116_[+2(1.21e-05)]_35_\
    [-5(6.45e-05)]_7_[+7(9.87e-05)]_318_[-3(5.35e-05)]_392
HMPREF1120_00924                 3.19e-02  41_[-7(4.40e-05)]_473_\
    [+2(1.60e-07)]_238_[+3(1.77e-05)]_156
HMPREF1120_01403                 6.85e-02  6_[-2(7.47e-05)]_146_[+1(6.99e-06)]_\
    40_[-2(1.63e-05)]_318_[-1(3.49e-06)]_430
```

```
HMPREF1120_01833                  4.23e-04  364_[+7(5.58e-05)]_200_\
    [+2(3.85e-05)]_88_[+2(1.91e-05)]_6_[+2(1.04e-10)]_229
HMPREF1120_07608                  2.84e-03  415_[+6(6.81e-05)]_35_\
    [+1(1.77e-05)]_97_[-2(3.59e-05)]_16_[-2(7.10e-08)]_66_[+2(6.66e-06)]_145_\
    [-2(6.56e-05)]_83
HMPREF1120_01522                  9.39e-03  609_[-10(2.12e-05)]_162_\
    [+4(2.66e-08)]_68_[-5(5.99e-05)]_62
HMPREF1120_06047                  4.44e-03  424_[+3(1.60e-05)]_273_\
    [+4(5.63e-08)]_174_[+6(6.43e-05)]_3_[+2(9.04e-05)]_14
HMPREF1120_01923                  5.37e-02  74_[+1(1.77e-05)]_693_\
    [-3(1.60e-05)]_57_[+2(2.90e-05)]_125
HMPREF1120_05100                  4.42e-01  134_[-3(2.61e-05)]_404_\
    [+5(8.22e-05)]_401
HMPREF1120_09238                  5.69e-02  315_[+7(2.24e-05)]_427_\
    [-1(6.99e-06)]_148_[-1(3.21e-05)]_42
HMPREF1120_00374                  2.83e-01  40_[-7(3.39e-05)]_58_\
    [-10(9.33e-05)]_56_[-3(8.51e-06)]_92_[-3(6.32e-05)]_623
HMPREF1120_06528                  4.14e-02  543_[-1(1.05e-05)]_367_\
    [+3(6.61e-07)]_60
HMPREF1120_07676                  5.47e-02  514_[-3(3.78e-05)]_101_\
    [-2(7.71e-05)]_195_[-1(2.47e-05)]_110_[+1(1.77e-05)]_3_[+1(3.49e-06)]_8
HMPREF1120_00038                  2.15e-14  11_[+1(3.49e-06)]_67_[-1(6.99e-06)]_\
    28_[-2(7.71e-05)]_46_[+9(8.68e-05)]_105_[+9(1.44e-12)]_89_[-5(1.97e-10)]_\
    121_[-9(7.69e-05)]_336_[+2(2.99e-08)]_37
HMPREF1120_01099                  6.79e-03  446_[-1(3.49e-06)]_224_\
    [-5(6.77e-05)]_226_[-4(2.42e-06)]_35
HMPREF1120_05642                  3.09e-02  50_[-10(8.51e-05)]_194_\
    [-9(8.85e-05)]_154_[-2(7.70e-06)]_136_[-9(9.53e-05)]_219_[+5(1.68e-05)]_83_\
    [+3(9.42e-05)]_3
HMPREF1120_06290                  5.77e-02  84_[-6(9.24e-05)]_96_[-1(2.47e-05)]_\
    117_[-1(6.99e-06)]_19_[-2(6.15e-05)]_595
HMPREF1120_09136                  4.27e-01  42_[+6(8.51e-05)]_908
HMPREF1120_03296                  1.45e-01  334_[+3(6.86e-05)]_479_\
    [+4(8.72e-07)]_146
HMPREF1120_03603                  5.30e-04  301_[+5(3.20e-06)]_407_\
    [+2(7.80e-08)]_24_[-1(6.99e-06)]_89_[+1(3.54e-05)]_100
HMPREF1120_07937                  2.28e-01  463_[-3(2.61e-05)]_207_\
    [+9(1.54e-05)]_289
HMPREF1120_04140                  3.00e-03  166_[-8(1.27e-05)]_24_\
    [+3(8.51e-06)]_110_[+1(4.28e-05)]_52_[+2(6.56e-05)]_150_[-3(5.35e-05)]_48_\
    [-3(6.86e-05)]_113_[+10(2.82e-05)]_61_[-4(7.31e-05)]_83
HMPREF1120_05088                  2.47e-01  379_[+10(8.02e-05)]_388_\
    [-2(5.95e-05)]_173
HMPREF1120_01710                  3.57e-01  927_[+4(7.10e-06)]_53
HMPREF1120_03451                  1.21e-01  192_[-1(3.49e-06)]_268_\
    [-7(3.50e-05)]_440_[+9(3.64e-05)]_21
HMPREF1120_01909                  5.44e-01  873_[+3(1.31e-05)]_106
HMPREF1120_03636                  2.63e-01  46_[-7(4.33e-05)]_52_[-7(6.49e-06)]_\
    157_[-7(3.07e-05)]_595
HMPREF1120_01980                  5.73e-01  266_[-2(1.38e-05)]_135_\
    [-2(9.93e-05)]_557
HMPREF1120_03973                  3.29e-01  802_[-9(9.80e-06)]_178
HMPREF1120_01020                  1.99e-01  101_[+1(6.99e-06)]_75_\
    [-8(1.72e-05)]_530_[-1(6.99e-06)]_235
HMPREF1120_07372                  1.43e-02  1_[-8(4.89e-05)]_245_[+7(8.98e-06)]_\
    106_[-2(1.16e-05)]_163_[+1(2.47e-05)]_260_[+2(4.41e-05)]_83
HMPREF1120_00101                  1.55e-01  317_[+8(2.00e-05)]_86_\
    [-8(7.75e-05)]_95_[+6(1.30e-05)]_370
HMPREF1120_07003                  1.52e-01  46_[+3(8.05e-05)]_390_\
    [+3(1.02e-06)]_236_[+5(6.94e-05)]_246
HMPREF1120_08674                  3.17e-01  673_[+8(3.87e-06)]_286
HMPREF1120_04734                  2.71e-02  762_[+10(9.49e-07)]_40_\
    [+4(4.43e-05)]_113_[+1(3.49e-06)]_17
HMPREF1120_00197                  7.28e-01  1000
HMPREF1120_01451                  6.60e-01  264_[+8(3.56e-05)]_274_\
    [-3(5.35e-05)]_400
HMPREF1120_01182                  1.18e-02  123_[-7(2.35e-05)]_18_\
    [-7(9.29e-05)]_170_[-10(9.33e-05)]_213_[+4(2.05e-05)]_25_[-1(3.49e-06)]_\
    283
```

```
HMPREF1120_03772              1.06e-02  152_[-7(4.69e-05)]_50_\
    [-10(2.01e-05)]_50_[+3(7.44e-05)]_10_[+1(1.05e-05)]_271_[+3(1.06e-05)]_327
HMPREF1120_04068              1.49e-01  115_[+7(1.65e-05)]_286_\
    [-8(4.89e-05)]_508
HMPREF1120_01282              2.03e-01  130_[+7(8.60e-05)]_178_\
    [-3(9.42e-05)]_15_[-3(8.71e-05)]_256_[+1(5.35e-05)]_50_[+7(2.13e-05)]_220
HMPREF1120_05091              7.23e-01  1000
HMPREF1120_05843              1.78e-03  88_[+7(5.24e-05)]_195_\
    [+8(4.14e-05)]_78_[-5(1.96e-08)]_508
HMPREF1120_05820              8.40e-01  1000
HMPREF1120_03646              3.36e-01  557_[+1(3.54e-05)]_400_\
    [+4(1.26e-05)]_14
HMPREF1120_04805              9.95e-07  206_[-5(8.84e-05)]_109_\
    [+10(4.93e-15)]_606
HMPREF1120_01777              5.97e-03  77_[+5(1.92e-05)]_48_\
    [+10(7.35e-05)]_26_[+7(2.56e-05)]_151_[-1(7.79e-05)]_132_[+1(2.84e-05)]_\
    272_[-9(1.54e-05)]_62_[+2(1.63e-05)]_44
HMPREF1120_06780              3.39e-01  302_[+5(3.81e-05)]_302_\
    [+4(5.52e-05)]_336
HMPREF1120_07526              2.19e-01  46_[-7(2.20e-05)]_495_\
    [+1(8.86e-05)]_6_[-4(6.32e-05)]_374
HMPREF1120_00198              2.92e-01  266_[+10(9.19e-05)]_64_\
    [-1(3.49e-06)]_166_[+5(9.71e-05)]_416
HMPREF1120_05027              3.15e-01  814_[-1(2.47e-05)]_177
HMPREF1120_04430              3.09e-01  419_[-3(1.06e-05)]_171_\
    [+2(6.15e-05)]_368
HMPREF1120_07017              4.89e-03  116_[-10(8.51e-05)]_205_\
    [+4(5.38e-06)]_32_[+4(1.92e-06)]_80_[+4(1.04e-08)]_250_[-2(2.50e-05)]_197
HMPREF1120_09162              4.83e-09  263_[+10(3.40e-15)]_195_\
    [+3(1.96e-05)]_294_[-8(1.64e-05)]_64_[-1(4.98e-05)]_74
HMPREF1120_08770              2.35e-03  413_[-7(3.50e-05)]_406_\
    [+9(5.77e-10)]_111
HMPREF1120_06046              6.37e-01  611_[+1(2.47e-05)]_146_\
    [+6(8.99e-05)]_184
HMPREF1120_05715              1.38e-03  24_[+5(6.27e-08)]_278_\
    [+6(1.69e-05)]_109_[+8(2.36e-05)]_22_[-7(6.42e-05)]_386
HMPREF1120_01343              4.36e-01  836_[-4(3.12e-05)]_144
HMPREF1120_01315              4.25e-02  53_[-6(8.05e-05)]_232_\
    [-5(9.79e-06)]_179_[+8(1.61e-05)]_27_[+3(3.45e-05)]_119_[-5(1.59e-05)]_126_\
    [+5(7.65e-05)]_32
HMPREF1120_03196              2.29e-01  634_[-7(2.09e-05)]_61_\
    [-3(6.86e-05)]_165_[+7(5.32e-05)]_19
HMPREF1120_03454              2.11e-02  170_[+1(4.98e-05)]_49_\
    [-3(2.38e-05)]_67_[-2(8.76e-05)]_447_[-4(6.41e-06)]_[-2(2.50e-05)]_175
HMPREF1120_00902              1.98e-01  13_[-9(1.36e-05)]_281_\
    [+1(6.99e-06)]_677
HMPREF1120_02198              9.20e-01  886_[+4(3.03e-05)]_94
HMPREF1120_05841              2.80e-03  84_[-8(5.93e-05)]_360_\
    [-1(6.99e-06)]_5_[-3(5.35e-05)]_40_[+4(6.10e-06)]_133_[+2(3.01e-05)]_24_\
    [-6(8.51e-05)]_162_[+2(3.59e-05)]_9
HMPREF1120_07391              1.99e-02  307_[-7(1.65e-05)]_76_\
    [+8(2.48e-05)]_65_[+9(1.19e-06)]_441
HMPREF1120_00954              1.22e-01  238_[-5(2.32e-05)]_268_\
    [+1(2.47e-05)]_69_[+1(8.86e-05)]_313_[-5(6.05e-06)]_14
HMPREF1120_08769              2.29e-01  871_[+10(2.01e-05)]_14_\
    [+6(5.02e-05)]_26
HMPREF1120_07879              7.41e-03  462_[-1(6.38e-05)]_282_\
    [-1(3.49e-06)]_9_[+3(4.91e-05)]_1_[+3(1.45e-05)]_186
HMPREF1120_05009              8.09e-01  282_[+1(9.56e-05)]_709
HMPREF1120_03199              5.40e-01  493_[-10(2.48e-05)]_252_\
    [+7(8.74e-05)]_166
HMPREF1120_00196              3.42e-02  156_[+8(4.58e-05)]_61_\
    [-3(1.76e-06)]_34_[+7(6.63e-05)]_637
HMPREF1120_06146              1.21e-01  128_[+3(2.16e-05)]_372_\
    [+2(8.22e-05)]_175_[-6(3.20e-05)]_233
HMPREF1120_02306              6.47e-01  254_[-6(3.01e-05)]_696
HMPREF1120_01981              5.42e-01  357_[+2(9.93e-05)]_135_\
    [+2(1.38e-05)]_466
HMPREF1120_04784              5.03e-01  378_[-1(6.05e-05)]_463_\
```

```
    [-3(1.06e-05)]_87_[+3(4.13e-05)]_21
HMPREF1120_05366                 9.33e-01  414_[+9(9.02e-05)]_566
HMPREF1120_06238                 8.94e-13  160_[+10(6.75e-05)]_227_\
    [+8(4.08e-20)]_168_[-1(6.05e-05)]_83_[-3(4.13e-05)]_252
HMPREF1120_02122                 3.15e-01  405_[-1(3.49e-06)]_258_\
    [-4(6.04e-05)]_121_[-1(3.49e-06)]_156_[+4(3.59e-05)]_2
HMPREF1120_06617                 1.26e-01  62_[+3(1.31e-05)]_117_\
    [-7(1.46e-05)]_71_[-7(8.22e-05)]_266_[+1(1.77e-05)]_354
HMPREF1120_06465                 7.41e-03  461_[+1(3.49e-06)]_387_\
    [+3(3.35e-06)]_32_[-6(8.28e-05)]_40
HMPREF1120_07460                 1.71e-01  132_[+6(1.75e-05)]_490_\
    [+10(5.00e-05)]_289
HMPREF1120_03504                 8.47e-02  287_[-8(6.85e-05)]_189_\
    [-6(6.90e-05)]_24_[-4(2.46e-05)]_251_[+3(4.50e-05)]_117
HMPREF1120_06251                 6.64e-01  112_[-8(6.39e-05)]_1_[+6(8.16e-05)]_\
    796
HMPREF1120_06104                 1.77e-01  91_[+3(8.71e-05)]_164_\
    [+5(1.01e-05)]_684
HMPREF1120_05260                 3.58e-02  278_[-1(8.86e-05)]_323_\
    [+5(1.98e-05)]_29_[-3(2.96e-06)]_27_[+7(2.27e-05)]_3_[+3(1.31e-05)]_199
HMPREF1120_02938                 2.20e-02  124_[+7(2.20e-05)]_127_\
    [-9(1.45e-05)]_158_[+1(3.49e-06)]_55_[-1(3.91e-05)]_448
HMPREF1120_06484                 2.05e-02  359_[+1(3.49e-06)]_26_\
    [+9(4.83e-05)]_586
HMPREF1120_06164                 3.80e-01  70_[+4(4.32e-05)]_414_\
    [+1(6.05e-05)]_169_[+6(5.33e-05)]_268
HMPREF1120_06689                 2.84e-01  1_[-8(6.85e-05)]_430_\
    [-10(3.78e-06)]_489
HMPREF1120_01406                 2.74e-01  283_[-3(3.78e-05)]_85_\
    [+3(3.45e-05)]_361_[+2(2.90e-05)]_38_[+7(8.35e-05)]_120
HMPREF1120_00109                 8.89e-02  146_[+7(3.50e-05)]_146_\
    [-5(9.49e-05)]_452_[-5(9.61e-07)]_126
HMPREF1120_05453                 1.04e-01  51_[+4(3.99e-05)]_222_\
    [+2(3.02e-06)]_260_[-9(8.66e-06)]_406
HMPREF1120_04014                 1.79e-02  134_[+8(2.91e-06)]_109_\
    [-10(9.05e-05)]_147_[-7(8.22e-05)]_207_[-9(6.43e-05)]_40_[-5(7.47e-05)]_\
    173
HMPREF1120_06399                 7.25e-01  458_[-9(1.00e-05)]_522
HMPREF1120_00404                 8.83e-02  288_[-3(3.45e-05)]_180_\
    [-9(2.78e-06)]_491
HMPREF1120_06148                 7.27e-03  325_[+9(2.56e-05)]_196_\
    [-4(2.46e-05)]_301_[-4(6.49e-09)]_118
HMPREF1120_02792                 1.51e-01  86_[-1(2.47e-05)]_251_\
    [+1(3.49e-06)]_645
HMPREF1120_08633                 9.74e-02  9_[-3(2.87e-05)]_368_[-2(8.48e-05)]_\
    69_[-5(1.72e-05)]_390_[-4(8.44e-05)]_62
HMPREF1120_02853                 8.81e-01  58_[+3(8.71e-05)]_921
HMPREF1120_01156                 5.19e-04  100_[+1(8.86e-05)]_109_\
    [+1(4.61e-05)]_104_[+1(3.91e-05)]_130_[-5(6.05e-06)]_236_[+2(1.73e-08)]_\
    [-3(8.71e-05)]_92_[-8(6.47e-05)]_79
HMPREF1120_01982                 6.21e-02  441_[+3(6.32e-05)]_127_\
    [-1(6.99e-06)]_296_[-2(6.56e-05)]_85
HMPREF1120_07880                 1.11e-03  116_[-8(5.32e-06)]_214_\
    [+1(7.44e-05)]_42_[-2(1.99e-05)]_155_[-7(7.97e-05)]_82_[-3(1.18e-05)]_1_\
    [-3(1.10e-07)]_227
HMPREF1120_06804                 7.29e-02  92_[-6(4.52e-05)]_63_[+1(3.54e-05)]_\
    240_[-8(9.22e-05)]_302_[+1(6.99e-06)]_59_[-10(4.14e-05)]_96
HMPREF1120_02225                 2.58e-02  8_[-3(2.16e-05)]_235_[-1(4.98e-05)]_\
    321_[-3(4.13e-05)]_88_[+3(1.35e-06)]_36_[-7(1.65e-05)]_190
HMPREF1120_01029                 9.17e-02  14_[-2(9.62e-05)]_39_[+7(6.42e-05)]_\
    8_[-6(2.51e-05)]_291_[+7(4.40e-05)]_91_[-9(3.43e-05)]_366
HMPREF1120_03745                 1.39e-01  141_[-8(8.15e-05)]_263_\
    [+9(6.43e-05)]_165_[-8(8.10e-05)]_129_[+3(2.87e-05)]_7_[+3(6.32e-05)]_7_\
    [-1(4.98e-05)]_135
HMPREF1120_03411                 2.61e-02  145_[+8(2.21e-05)]_81_\
    [+1(3.54e-05)]_53_[-9(6.58e-05)]_170_[+2(9.33e-05)]_371_[+1(7.79e-05)]_80
HMPREF1120_05790                 1.31e-03  207_[+1(3.49e-06)]_219_\
    [+3(6.86e-05)]_122_[+5(4.15e-06)]_108_[-5(8.03e-05)]_109_[+3(8.51e-06)]_53_\
    [-5(4.55e-05)]_11
```

```
HMPREF1120_07971                 3.04e-02  289_[+2(3.59e-05)]_100_\
    [+1(8.86e-05)]_114_[-7(1.51e-05)]_393_[+3(3.78e-06)]_3
HMPREF1120_02802                 3.12e-01  118_[+5(1.82e-05)]_297_\
    [+8(2.32e-05)]_504
HMPREF1120_01015                 6.94e-02  71_[-7(1.51e-05)]_264_\
    [+1(9.56e-05)]_248_[+10(3.87e-05)]_51_[-1(5.35e-05)]_259
HMPREF1120_07241                 5.80e-02  98_[+9(8.03e-05)]_165_\
    [+3(7.61e-06)]_358_[+1(3.49e-06)]_192_[-3(1.96e-05)]_1_[+3(2.87e-05)]_94
HMPREF1120_01137                 6.47e-01  685_[+6(6.43e-05)]_61_\
    [-7(5.94e-05)]_154
HMPREF1120_01523                 1.22e-02  182_[+6(3.48e-06)]_329_\
    [-10(4.63e-05)]_204_[+1(2.47e-05)]_148_[+3(9.49e-06)]_18
HMPREF1120_03195                 4.07e-01  147_[+2(6.78e-05)]_82_\
    [+3(8.51e-06)]_729
HMPREF1120_08439                 2.92e-01  141_[+5(5.84e-05)]_74_\
    [+1(3.91e-05)]_395_[-2(6.78e-05)]_320
HMPREF1120_04980                 1.15e-01  7_[-9(8.52e-05)]_270_[+1(2.84e-05)]_\
    500_[+6(8.99e-05)]_55_[+4(6.32e-05)]_69
HMPREF1120_08783                 1.78e-02  39_[-1(3.49e-06)]_128_\
    [+9(9.01e-06)]_221_[-3(2.87e-05)]_334_[-3(1.18e-05)]_36_[-1(3.54e-05)]_76_\
    [-2(6.78e-05)]_29_[+1(2.47e-05)]_27
HMPREF1120_07216                 4.87e-01  837_[+7(7.50e-05)]_113
HMPREF1120_03803                 5.44e-08  339_[-6(1.62e-05)]_16_\
    [+3(1.68e-11)]_36_[-10(7.90e-05)]_339_[-3(8.05e-05)]_37_[+1(3.49e-06)]_47_\
    [-9(9.05e-07)]_26
HMPREF1120_04197                 2.57e-01  394_[+9(3.23e-05)]_359_\
    [+6(3.72e-06)]_177
HMPREF1120_07677                 3.57e-01  82_[-3(1.77e-05)]_771_\
    [-6(6.34e-05)]_76
HMPREF1120_06028                 1.99e-02  922_[-3(3.51e-08)]_57
HMPREF1120_06922                 6.91e-02  333_[-5(5.70e-05)]_138_\
    [+4(8.44e-05)]_150_[+9(1.73e-05)]_88_[+4(3.40e-05)]_66_[+3(3.45e-05)]_104
HMPREF1120_06027                 5.19e-03  233_[+1(1.77e-05)]_221_\
    [-7(1.17e-05)]_14_[+3(3.51e-08)]_452
HMPREF1120_04736                 1.81e-01  181_[+9(9.53e-05)]_265_\
    [-7(4.77e-05)]_362_[+2(4.20e-06)]_101
HMPREF1120_08058                 9.80e-01  1000
HMPREF1120_03622                 9.05e-02  179_[-3(4.27e-06)]_61_\
    [+10(4.59e-06)]_48_[+10(2.35e-05)]_613
HMPREF1120_00523                 1.28e-02  146_[+9(7.06e-05)]_137_\
    [+1(6.99e-06)]_174_[+3(2.61e-06)]_185_[+3(2.59e-07)]_134_[-1(6.38e-05)]_\
    144
HMPREF1120_06308                 9.57e-03  452_[+2(6.46e-08)]_392_\
    [+5(4.86e-06)]_95
HMPREF1120_04947                 8.76e-06  219_[-4(1.76e-05)]_259_\
    [+3(6.80e-06)]_156_[+4(8.78e-12)]_243_[+10(3.03e-05)]_23
HMPREF1120_01818                 2.92e-01  187_[-3(6.86e-05)]_20_\
    [-1(6.99e-06)]_113_[-1(2.84e-05)]_641
HMPREF1120_01896                 9.87e-02  214_[-9(4.33e-05)]_6_[-7(5.00e-05)]_\
    41_[+4(6.45e-05)]_100_[+3(8.05e-05)]_190_[+3(2.61e-05)]_138_[+1(6.05e-05)]_\
    170
HMPREF1120_03417                 2.23e-02  62_[-3(2.38e-05)]_766_\
    [+1(3.49e-06)]_142
HMPREF1120_07182                 2.47e-03  429_[-6(8.16e-05)]_73_\
    [-1(6.99e-06)]_98_[-2(1.55e-08)]_320
HMPREF1120_08026                 7.34e-01  493_[+3(6.86e-05)]_486
HMPREF1120_04558                 5.19e-02  312_[+1(6.05e-05)]_31_\
    [+4(1.34e-05)]_58_[-10(9.94e-05)]_82_[-1(8.86e-05)]_7_[+2(9.04e-05)]_3_\
    [+7(5.50e-05)]_97_[-4(9.74e-05)]_242
HMPREF1120_04424                 2.82e-01  51_[+1(7.79e-05)]_631_\
    [+3(1.60e-05)]_288
HMPREF1120_00714                 6.05e-01  315_[-7(5.32e-05)]_635
HMPREF1120_06026                 3.45e-01  697_[-5(9.05e-05)]_263
HMPREF1120_05486                 2.09e-01  332_[+1(1.05e-05)]_57_\
    [+1(6.99e-06)]_547_[+3(1.45e-05)]_25
HMPREF1120_02756                 5.31e-02  522_[+1(3.49e-06)]_20_\
    [-2(9.93e-05)]_62_[-4(6.18e-05)]_171_[+2(2.50e-05)]_154
HMPREF1120_02623                 1.09e-04  188_[+3(1.96e-05)]_1_[-1(7.44e-05)]_\
    176_[+3(4.13e-05)]_11_[-3(6.80e-06)]_34_[-5(2.57e-09)]_83_[-3(2.61e-06)]_\
```

```
    58_[+3(8.84e-07)]_89_[+3(3.15e-05)]_95_[+3(1.35e-06)]_8_[+7(3.34e-05)]_11
HMPREF1120_08078                 2.76e-01  64_[+3(6.80e-06)]_12_[+7(9.15e-05)]_\
    349_[-7(9.57e-05)]_300_[-7(3.87e-05)]_35_[-10(5.62e-05)]_30
HMPREF1120_04804                 2.39e-01  124_[-10(4.07e-05)]_472_\
    [-2(4.89e-05)]_44_[+3(2.16e-05)]_279
HMPREF1120_06752                 4.77e-01  68_[-9(4.83e-05)]_148_\
    [-6(2.13e-05)]_118_[+3(9.42e-05)]_145_[+3(5.35e-05)]_409
HMPREF1120_04359                 4.03e-02  258_[-3(1.06e-05)]_124_\
    [+1(1.05e-05)]_58_[-2(4.57e-05)]_68_[+2(5.95e-05)]_420
HMPREF1120_05433                 3.11e-01  316_[-10(4.85e-05)]_363_\
    [+2(9.93e-05)]_157_[-6(3.89e-05)]_54
HMPREF1120_02391                 1.99e-01  396_[-7(3.87e-05)]_107_\
    [-1(3.49e-06)]_438
HMPREF1120_08476                 2.64e-02  21_[+10(1.26e-05)]_170_\
    [-5(9.95e-05)]_306_[-1(8.86e-05)]_258_[-3(1.45e-05)]_84_[-1(6.99e-06)]_21_\
    [-3(3.15e-05)]_1
HMPREF1120_05622                 2.39e-03  71_[-2(2.60e-05)]_31_[+1(3.49e-06)]_\
    311_[+5(4.11e-05)]_116_[-3(1.17e-06)]_333_[-2(6.78e-05)]_26
HMPREF1120_01983                 3.43e-02  147_[-6(4.26e-05)]_103_\
    [+7(3.39e-06)]_100_[-10(9.63e-05)]_361_[+4(9.35e-05)]_130
HMPREF1120_05846                 9.97e-01  1000
HMPREF1120_05848                 8.12e-01  645_[-1(8.86e-05)]_155_\
    [-1(3.21e-05)]_182
HMPREF1120_03154                 1.53e-01  609_[-8(4.25e-05)]_203_\
    [-7(6.03e-06)]_97
HMPREF1120_00901                 4.27e-01  185_[-7(2.56e-05)]_64_\
    [-8(7.78e-05)]_660
HMPREF1120_03453                 3.55e-01  7_[+5(9.95e-05)]_219_[+8(4.36e-05)]_\
    693
HMPREF1120_06719                 9.03e-01  1000
HMPREF1120_01453                 2.57e-01  105_[+8(6.55e-05)]_561_\
    [-1(2.47e-05)]_255_[+1(8.86e-05)]_20
HMPREF1120_01601                 1.86e-01  56_[+2(4.12e-05)]_536_\
    [+1(3.49e-06)]_378
HMPREF1120_07322                 2.65e-02  288_[-2(3.60e-07)]_159_\
    [+1(8.86e-05)]_15_[+1(8.86e-05)]_461_[+3(1.18e-05)]_17
--------------------------------------------------------------------------------

********************************************************************************


********************************************************************************
Stopped because requested number of motifs (10) found.
********************************************************************************

CPU: meme-server

********************************************************************************
```
